# Bringing New Methods to the Seed Proteomics Platform: Challenges and Perspectives

**DOI:** 10.3390/ijms21239162

**Published:** 2020-12-01

**Authors:** Galina Smolikova, Daria Gorbach, Elena Lukasheva, Gregory Mavropolo-Stolyarenko, Tatiana Bilova, Alena Soboleva, Alexander Tsarev, Ekaterina Romanovskaya, Ekaterina Podolskaya, Vladimir Zhukov, Igor Tikhonovich, Sergei Medvedev, Wolfgang Hoehenwarter, Andrej Frolov

**Affiliations:** 1 Department of Plant Physiology and Biochemistry, St. Petersburg State University; 199034 St. Petersburg, Russia; g.smolikova@spbu.ru (G.S.); bilova.tatiana@gmail.com (T.B.); s.medvedev@spbu.ru (S.M.); 2 Department of Biochemistry, St. Petersburg State University; 199178 St. Petersburg, Russia; daria.gorba4@yandex.ru (D.G.); elena_lukasheva@mail.ru (E.L.); gm2124@mail.ru (G.M.-S.); oriselle@ya.ru (A.S.); alexandretsarev@gmail.com (A.T.); rcatherine@mail.ru (E.R.); 3 Department of Bioorganic Chemistry, Leibniz Institute of Plant Biochemistry; 06120 Halle (Saale), Germany; 4 Institute of Analytical Instrumentation, Russian Academy of Science; 190103 St. Petersburg, Russia; ek.podolskaya@gmail.com; 5 Institute of Toxicology, Russian Federal Medical Agency; 192019 St. Petersburg, Russia; 6 All-Russia Research Institute for Agricultural Microbiology; 196608 St. Petersburg, Russia; vladimir.zhukoff@gmail.com (V.Z.); arriam2008@yandex.ru (I.T.); 7 Department of Genetics and Biotechnology, St. Petersburg State University; 199034 St. Petersburg, Russia; 8 Proteome Analytics Research Group, Leibniz Institute of Plant Biochemistry, 06120 Halle (Saale), Germany; wolfgang.hoehenwarter@ipb-halle.de

**Keywords:** data processing, gel-based proteomics, gel-free proteomics, glycation, glycosylation, phosphorylation, post-translational modifications, proteomics

## Abstract

For centuries, crop plants have represented the basis of the daily human diet. Among them, cereals and legumes, accumulating oils, proteins, and carbohydrates in their seeds, distinctly dominate modern agriculture, thus play an essential role in food industry and fuel production. Therefore, seeds of crop plants are intensively studied by food chemists, biologists, biochemists, and nutritional physiologists. Accordingly, seed development and germination as well as age- and stress-related alterations in seed vigor, longevity, nutritional value, and safety can be addressed by a broad panel of analytical, biochemical, and physiological methods. Currently, functional genomics is one of the most powerful tools, giving direct access to characteristic metabolic changes accompanying plant development, senescence, and response to biotic or abiotic stress. Among individual post-genomic methodological platforms, proteomics represents one of the most effective ones, giving access to cellular metabolism at the level of proteins. During the recent decades, multiple methodological advances were introduced in different branches of life science, although only some of them were established in seed proteomics so far. Therefore, here we discuss main methodological approaches already employed in seed proteomics, as well as those still waiting for implementation in this field of plant research, with a special emphasis on sample preparation, data acquisition, processing, and post-processing. Thereby, the overall goal of this review is to bring new methodologies emerging in different areas of proteomics research (clinical, food, ecological, microbial, and plant proteomics) to the broad society of seed biologists.

## 1. Introduction

Seeds represent the basis of the human diet and contribute daily to consumed foods [1]. Hence, the rapidly growing human population requires a secure, continuous supply with foods of appropriate quality and safety [2]. Because of this, modern agriculture aims at sustainable production of high-quality seeds. On the one hand, due to the ongoing climate changes, it can be a challenging task [3]. On the other hand, the improvement of crop plant productivity is desired [4,5]. Hence, the yields of crop biomass and tolerance to environmental stress need to be increased simultaneously. For this, understanding of the fundamental processes accompanying seed development, storage, and germination, is required.

During development, the seed passes through the stages of embryogenesis, filling, and late maturation [6]. Importantly, the last steps of seed maturation are accompanied by development of desiccation tolerance and onset of dormancy—events that affect the tolerance of seeds to storage conditions thus having a great impact on their quality [7]. In turn, environmental stressors, like drought, high salinity, and high light, directly affect maturation of seeds and might compromise their longevity, i.e., ability to maintain viability during storage [8,9]. To a large extent it is underlaid by stress-related metabolic adjustment, i.e., accumulation of osmoprotective amino acids and sugars in plant tissues [10]. In combination with oxidative stress, typically accompanying plant response to abiotic stressors, this might lead to an increased oxidative and glycoxidative modification of seed proteins, that might negatively affect quality of seeds [11]. An understanding of mechanisms underlying these processes is required to ensure sustainable production of seed crops.

Seed germination and seedling development are largely dependent on the metabolic status of the plant, especially availability of reserve substances [12,13,14,15,16]. The chemical nature of these biomolecules differs between plant species. For instance, in cereals, the major seed storage tissue is endosperm, where starch can account for up to 87% of the total dry seed weight [14,17]. In contrast, in legumes, the principle storage organs are cotyledons, which, for example, in soybean, can contain up to 40 and 20% (*w*/*w*) proteins and oils, respectively [17]. Therefore, due to high constitutive levels of protein biosynthesis, legume seeds represent an excellent platform for the large-scale production of transgenic proteins [16,18,19]. As legume seeds are widely used for human nutrition and animal feed [20], their development is of particular interest to plant biologists. The model plants most commonly used for this are pea (*Pisum sativum*), faba bean (*Vicia faba*), soybean (*Glycine max*), lotus (*Lotus japonicus*), and annual barrel medic (*Medicago truncatula*) [14].

In this context, knowledge of the seed proteome of legume crop plants, as well as its dynamics in response to environmental and biological stressors, may significantly impact the improvement of their quality and nutritional properties [21]. Therefore, such versatile functional genomics tool as proteomics is widely used in seed research [11,22,23,24,25,26,27,28]. Currently, it is routinely employed in comprehensive profiling of complex protein extracts and delivers valuable qualitative and quantitative information on protein dynamics in plant organisms. Implementation of cell fractionation techniques allows reasonable simplification of the protein sample matrix and provides better insight into the molecular organization of individual compartments, as well as macromolecular complexes like cytoskeleton, membranes, and cell wall [29,30,31,32,33]. More specifically, the global proteomic approach addresses alterations in multiple biological processes, occurring sequentially and in parallel at the tissue, cell or subcellular level [24,34,35]. Based on the acquired information, development of seeds and seedlings can be systematically characterized [36,37]. Because of this, the molecular basis of seed vigor and its alterations in response to developmental or environmental stimuli have been intensively studied over the past decade [15,28,38,39,40,41,42,43,44,45], although the key proteins associated with seed vigor are still poorly understood. One of the most possible reasons for this is the high complexity of seed proteome compared with highly abundant storage polypeptides and low-abundant regulatory and signaling proteins. For this reason, depletion of storage proteins [46] and enrichment of low-abundant post-translationally modified species [47] is desired.

Thus, here we address the proteomics techniques currently established in plant science and potentially applicable to seed research. Here we discuss methodological aspects, with a special emphasis on sample preparation, data acquisition, processing, and post-processing. We consider both the methods and techniques already successfully introduced in seed proteomics, as well as those prospectively applicable and promising in this field.

## 2. Seed Proteomics: General Methodology and Applications

The proteome is a complex system, representing a result of interconnected dynamic properties of individual proteins [48]. In agreement with this, proteomics aims at qualitative and/or quantitative characterization of the proteome, with the depth defined by the type and complexity of the specific research aim. Accordingly, so-called discovery proteomics is focused on maximizing sampling depth, annotation of individual proteins, expression patterns of their isoforms, as well as identity and precise localization of post-translational modifications (PTMs) [11,49]. Discovery proteomics is inherently hypothesis-generating, implementing relative or absolute quantification of individual proteins (protein dynamics), in response to internal or external factors [50]. Although to some extent, these tasks can be solved by other analytical techniques (e.g., immunochemical methods, like ELISA or Western blotting) [51], the most efficient way it can be achieved is by mass spectrometry coupled on-line with high-resolution separation techniques [52]. Thereby, protein identity can be assigned by tandem mass spectrometry (MS/MS), i.e., fragmentation of peptide ions, pre-selected in so-called full-MS survey scans (data-dependent acquisition, DDA) or acquired in parallel (data-independent acquisition, DIA) [53,54]. Further post-processing with versatile bioinformatic tools gives access to functions and intracellular localization of individual seed proteins, as well as their involvement in complex protein-protein interaction networks [55].

In general, proteomic analysis can rely either on top-down or bottom-up strategies [56,57]. In the first case, ions of individual proteins with molecular weight below 25 kDa are measured directly, including fragmentation and MS/MS acquisition of protein fragment ions [58,59], whereas the bottom-up strategy employs proteolytic digestion of protein mixtures prior to MS and MS/MS analysis of the resulting proteolytic peptide-specific ions [60]. Thereby, identification of proteins by bottom-up proteomics relies on sequence tags, comprising exact monoisotopic mass, charge, and MS/MS fragmentation patterns acquired for peptide ions [61]. As application of top-down proteomics is restricted by molecular weight and purity requirements for analyte proteins, as well as simply on the protein size and charge abundance [56], its use in seed proteomics is limited [62]. Thus, in the absolute majority of cases, seed proteomics relies on the bottom-up strategy [20,26,63,64].

Obviously, the high complexity of the eukaryotic proteome represents the greatest challenge in proteomics [65]. Therefore, powerful mass spectrometric techniques, used for proteome analysis, need to be complemented by high-resolution and high-throughput separation methods. The major challenge in seed proteomics is the high abundance of storage proteins, which strongly dominate the seed proteome and can be used as protein quality markers [66]. These proteins need to be depleted, before the low-abundance seed polypeptides can be accessed [63]. A variety of depletion techniques, applicable for seed storage proteins, were comprehensively reviewed by Miernyk recently [26]. This can be accomplished by extraction with aqueous (aq.) isopropanol-based solvents [67], or by precipitation in presence of aq. 0.01–0.1% (*w*/*v*) protamine sulfate [68]. The latter technique proved to be well-compatible with label-free [63] and tag-based [69] quantitative techniques. Alternatively, seed storage globulins (glycinin and ß-conglycenin) can be efficiently removed by 10 mmol/L CaCl_2_ [70]. Selective enrichment of minor seed proteins can also be achieved by implementation of the combinational peptide ligand libraries technology [71]. Finally, reduction of sample complexity can be achieved by centrifugation-based fractionation of total protein extracts, as it was done to obtain the lipid droplet-enriched protein fraction from tobacco seeds [72]. Recently, Du et al. proposed an alternative approach—absorption on a polyvinylidene fluoride (PVDF) membrane for isolation of lipid body-associated proteins from maize seeds [73].

**Figure 1:**
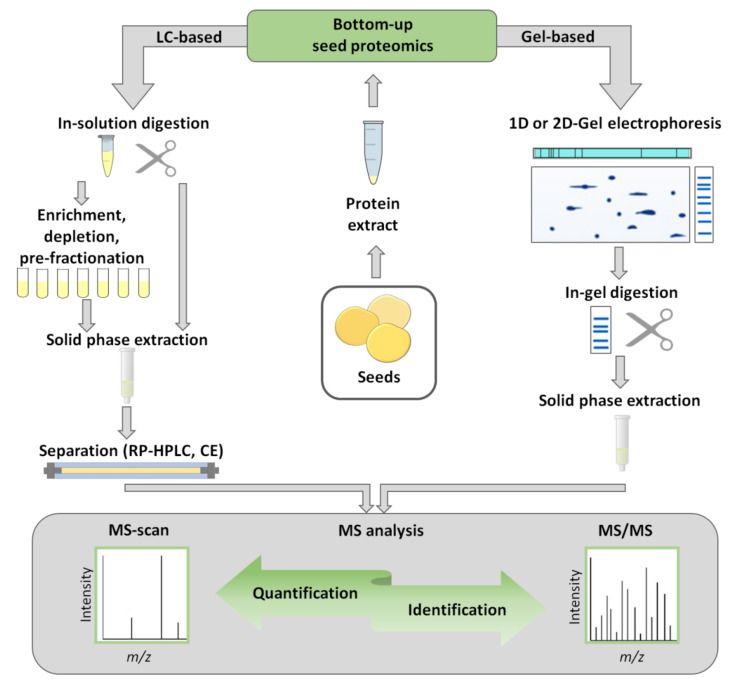
The overview of the experimental workflows for gel-based and gel-free proteomics.

In general, separation methods employed in proteomics can be attributed to either (i) gel-based or (ii) gel-free strategies [74], clearly different in their methodological setup: gel-based methods assume separation at the protein level, whereas the gel-free approach relies on limited enzymatic hydrolysis prior to separation (Figure 1).

Accordingly, the first group of methods relies on polyacrylamide gel electrophoresis in sodium dodecyl sulfate (SDS-PAGE) or two-dimensional gel electrophoresis (2D-GE), whereas the second one typically employs liquid chromatography (LC). Alternatively, gel-free electrophoretic techniques, such as free-flow or capillary electrophoresis can be used for protein or peptide separation (the methodology is comprehensively reviewed by Dawod et al. [75]), although in plant proteomics these methods are usually applied to analysis of membrane proteins, not to the seed proteome [76].

## 3. Gel-Based Bottom-Up Proteomics

Due to relatively low analytical resolution (i.e., number of bands reliably detectable in electropherograms) of SDS-PAGE, gel-based proteomics typically relies on 2D-GE [77,78], i.e., a two-step procedure, sequentially employing isoelectrofocusing (IEF) for separation of proteins by isoelectric point, followed by SDS-PAGE for separation by molecular weight (Figure 1). To establish pH gradients, required for IEF, protein samples are solubilized in aqueous buffers supplemented with carrier-ampholytes, chaotropic agents, and detergents [79]. Most often, 7–8 mol/L urea and 2 mol/L thiourea are used as chaotropic agents (i.e., compounds, disrupting the hydrogen bonding network of water) [80] whereas 3-(3-cholamidopropyl)dimethylammonio)-1-propanesulfonate (CHAPS) [81] and Triton X-100 are typically used as detergents (i.e., substances, disrupting intra- and inter-molecule non-polar interactions) [82].

All along with its good compatibility with highly-sensitive visualization and MS-based identification techniques, during the last decades 2D-GE became a powerful and versatile tool for bottom-up proteomics. It allows detection of thousands of proteins in one experiment [83]. Thereby, in both separation steps the resolution of 2D-GE can be adjusted to the specific needs of a proteomics experiment [84]. Thus, selection of a smaller pH range can give better insight into the fractions of basic, neutral or acidic proteins, with longer immobilized pH gradient (IPG) strips giving better spot resolution [85]. At the level of PAGE separation, better resolution can be achieved by increase of gel size. In contrast to gel-free techniques, 2D-GE gives access to the patterns of protein isoforms and relation of isoforms to specific post-translational modifications (PTMs)–phosphorylation, glycosylation, oxidation [86]. Another important feature of 2D-GE is the high reliability of protein quantification, its independence from matrix effects and the availability of orthogonal visualization techniques [87].

On the other hand, 2D-GE has several important limitations. First of all, reproducibility of the method is often compromised, that can be related to inhomogeneity of the gel or cathodic drift, i.e., progressive loss of basic proteins during prolonged application of electric field during electro-focusing [83]. Not less importantly, two-dimensional separation is highly dependent on sample preparation, and inconsistency between replicates at this step might result in high variations in electrophoretic mobility of individual proteins [87]. Finally, it is difficult to resolve the whole proteome in one experiment, as the proteins behind the pI gradient, applied during IEF, remain unseparated [88]. Low abundant and hydrophobic proteins are also often undersampled by 2D-GE.

### 3.1. Sample Preparation for Gel-Based Proteomics

Protein extraction procedures, conventionally applied in seed proteomics, are usually designed in agreement with this reconstitution scheme (Table A1). To achieve high efficiency of protein extraction, seed tissues are usually shock-frozen in liquid nitrogen and homogenized with a mortar and pistil or/and in a ball mill [26]. Among the variety of available techniques, phenol extraction and trichloroacetic acid (TCA)/acetone precipitation are the most effective methods to achieve comprehensive protein isolation [89]. For quantitative solubilization of seed proteins (especially membrane ones), detergents (e.g., SDS or Triton X-100) need to be added [90,91]. As was explicitly demonstrated, application of detergents is strongly mandatory for reconstitution of seed proteins [92,93]. Technically, TCA/acetone procedure is easier—it relies on direct precipitation of proteins from plant tissues. The efficiency of this approach was demonstrated in the analysis of germinating wheat seeds [94]. Interestingly, as a precipitation agent, acetone can be also used as it is (without TCA) or such extraction can be complemented with a MeOH/CHCl_3_/H_2_O procedure—this approach was shown to be advantageous in terms of proteome coverage in the study of quinoa seeds [95]. In contrast, phenol extraction is a two-step procedure, i.e., (i) solubilization of proteins with phenol and (ii) precipitation with methanol afterwards [55]. In comparison to other extraction methods, the TCA/acetone precipitation proved to be highly efficient in the proteome analysis of protein-rich seeds, as was demonstrated for soybean seed protein preparations [96]. This method was also applicable for wheat and rice seeds, which are characterized with a high content (90–95%) of starch and low protein (4–10%) and lipid (about 1%) content [97,98]. On the other hand, although the phenol-based methods might suffer from compromised recovery, this approach provides better purity, in comparison to acetone/TCA extraction. It also ensures reliable removal of anionic polysaccharides and nucleic acids, which might interfere with the 2D-GE procedure [99].

Importantly, while working with whole seeds, special attention needs to be paid to removal of secondary metabolites, especially phenolics, which are typically abundant in seed coats and might react with proteins, affecting electrophoretic separation [100]. This is especially important for the study of berries when it comes to the proteome of the secondary cell wall (seed coat), also rich in anthocyanins, tannins, terpenes [101]. Oxidative damage of proteins by phenolics can be suppressed by the addition of soluble or non-soluble polyvinylpyrollidone (PVP) to the extraction buffer [89] or (even better) by supplementation of PVP directly to plant samples prior to tissue grinding [102]. The effect of this procedure was demonstrated in a proteomics study, dealing with the effect of rhizobia on pea seed productivity [103]. Reducing agents, such as mercaptoethanol, dithiothreitol (DTT), and ascorbic acid are added as well [104], although the latter can promote oxidation and glycation of amino acid side chains in the presence of transition metals, as was shown with different in vitro glycation models [105,106,107]. It is important to note, that despite its excellent performance, phenol extraction suffers from low sample throughput and requires some experience in handling.

Most other protein extraction methods provide lower protein yields, incomplete protein recovery, and/or compromised purity. Easiest, ground plant tissues can be extracted directly with sample buffer (sodium or potassium phosphate with pH close to neutral) containing urea and usually (but not always) thiourea. This method proved to be applicable for extraction of legume seed proteins [95,96]. To achieve higher recovery of membrane proteins, sample buffer can be supplemented with CHAPS (e.g., for analysis of immature *Medicago truncatula* seeds) [82], Triton X-100 (as was applied in profiling of germinating soybean seeds) [108], Nonidet P40 (NP-40, e.g., used in the analysis of dormant and germinating *Arabidopsis thaliana* seeds) [109], or sodium dodecyl sulfate (SDS) in the presence of 20% (*v*/*v*) glycerol, successfully applied in the evaluation of persulfide metabolism in developing Arabidopsis seeds [110]. To remove lipids (which can reduce solubility of proteins and affect thereby IEF separation [111]), pre-cleaning with petroleum ether [112] or dichloromethane (DCM) [113] can be implemented in the protocol of isolation proteins from legume seeds. Resulting pre-cleaned extracts can be analyzed directly [114] or further purified, e.g., by precipitation with TCA/acetone, as was applied in the multi-omics study of rice seeds [115]. Alternatively, soluble protein fractions can be extracted by aqueous buffers (typically Tris-HCl or HEPES), further precipitated by acetone, and dried. This method gives access, in particular, to the soluble part of the proteome, which was successfully used to study the reserve mobilization of proteins [108].

It is important to note, that different seed matrices require different isolation protocols to achieve the best possible results. For example, Bose et al. showed that extraction of seed proteins in cereal species requires special considerations in respect of isolation buffer composition: for example for wheat the presence of a chaotropic agent (urea) is advantageous, whereas for rye a Tris-HCl buffer yields better proteome coverage [64]. Additionally, a step-wise extraction procedure to obtain water-soluble (albumins), salt-soluble (globulins), alcohol-soluble prolamins (ethanol extracted), and alcohol-insoluble prolamins (NaOH extracted)—so-called Osborn protocol [116], is efficient in the extraction of proteins from stark-rich seeds [117]. Chaotropic agents (urea, thiourea) and polyvinylpyrrolydone need to be added when cell wall-associated proteins are isolated [71].

### 3.2. Visualization of Electrophoretic Zones in Gel-Based Proteomics

In an electropherogram, separated proteins can be visualized as patterns of characteristic signals—so-called “spots”, visible with the eye or in fluorescence detection mode, respectively [56]. Thus, an appropriate visualization of separated spots becomes critical for successful interpretation of 2D-GE data. Usually, when only major proteins are targeted, corresponding spots are visualized with colloidal Coomassie Blue dye [118], which is completely compatible with MS and, according to our own validation experiments, provides a sensitivity of several dozen nanograms per electrophoretic zone [119]. This technique suits well to the detection of seed proteins and was proven to give reliable quantitative results [120]. When higher sensitivity is desired, various silver staining methods can be employed [121]. This technique is highly sensitive and provides reliable protein detection in the femtomol range [122]. Thereby, formaldehyde-free protocols allow reliable MS-based identification of separated proteins [123], that were successfully applied to detection of proteins in seeds and cold-pressed oils [124]. One must keep in mind, that this method suffers from low linearity [79]. Therefore, fluorescent dyes, like SYPRO™ Ruby [125] and Ruthenium red [126] stains, are advantageous, when quantification is desired. Indeed, such reagents provide sensitivity, close to silver staining, and a linear dynamic range of up to three orders of magnitude [127], being well applicable to gel-based separations of seed proteins [128]. Obviously, due to the high number of detected signals, highly-sensitive methods ultimately require higher resolution. In the best case scenario, 2D-GE separation allows distinction of up to 10,000 spots per experiment [129,130]. Remarkably, as 2D-GE analysis relies on sequential separation by isoelectric point and molecular weight, these two parameters can be easily derived from 2D-electropherograms, and provide, thereby, a further level of protein annotation, in addition to MS- and MS/MS analysis of individual spots [23]. These data can be efficiently applied to cross-validation of results, obtained by gel-free techniques in combination with gel-based/gel-free workflows, increasing, thereby, the overall proteome coverage and identification reliability [120]. It is important to note, that the number of detected spots does not directly correspond to protein identification rates. Indeed, on one hand, polypeptides with similar pI and Mr tend to co-migrate in both separation dimensions. On another, the proteins, represented by several isoforms usually appear as characteristic signal patterns, slightly different in one or both parameters. These signals can also be represented by truncated and post-translationally modified protein variants, which can be in special cases assigned by a combination of Western blotting and tandem mass spectrometry (MS/MS) [131]. Thus, 2D-GE gives valuable information about qualitative and quantitative isoform patterns [34], although the overall numbers of non-redundant proteins, identified in individual 2D-GE, is typically restricted to several hundred [11]. This limitation can be, however, overcome by the implementation of enrichment/depletion and pre-fractionation techniques, which allow detection of overall higher numbers of spots [68].

### 3.3. Identification of Individual Proteins by Mass Spectrometry

**Figure 2:**
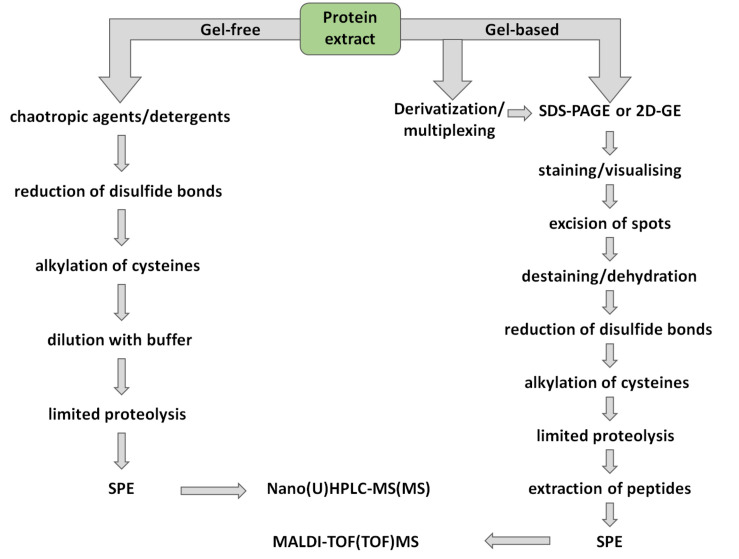
Overview of enzymatic digestion implemented in gel-based and gel-free experimental setup.

After excision of individual spots, the corresponding proteins can be subjected to limited proteolysis [20]. The digestion protocols typically rely on a well-established procedure, only slightly varying depending on specific experimental setup (Figure 2). This methodology is well-established in plant proteomics [132] and specifically in seed applications [133] as a modification of the procedures, developed earlier for analysis of animal tissues [134]. Thus, when Coomassie dye is used for gel staining, the workflow includes excision of spots, cutting them in small sections, and de-staining by repeated incubation in aqueous methanol- or acetonitrile-containing buffers (Table A2) [135]. After de-staining, gels are dried at room temperature and rehydrated with a protease solution [136]. The dried gel pieces, swollen in protease solution efficiently absorb enzyme, thereby facilitating proteolysis and increasing its efficiency. After completing the digestion, proteolytic peptides can be recovered by repeated dehydration and re-hydration of gel sections. For silver nitrate stained gels, no de-staining procedure prior to mass spectrometry is necessary [137]. However, only formaldehyde-free techniques can be applied, when proteolysis and MS-based characterization of hydrolysates are desired [122]. Importantly, while reduction of disulfide bonds and alkylation of resulted sulfhydryls can be done during sample preparation (Table A2), as can be exemplified by the proteomic characterization of lupin seeds [138]), this step is often implemented after excision of gel slices [31,139,140]. The main limitation of the conventional in-gel digestion workflow is its somewhat limited throughput. However, recently, this was successfully overcome with the introduction of the high-throughput in-gel digestion (HiT-Gel) technique, based on a 96-well plate format (Table A2) [141], which still needs to be established in seed proteomics.

Identification of proteins in tryptic hydrolyzates relies on mass spectrometric (MS) analysis of their components, i.e., proteolytic peptides. Thereby, for reliable identification and quantification of each individual protein, at least one peptide needs to be proteotypic, i.e., uniquely identifying a specific protein and consistently detectable during MS analysis [142]. Thus, mass spectrometric assays, based on such peptides, provide high sensitivity and specificity, being a good alternative to more time-consuming immunochemical techniques [143,144]. As each excised electrophoretic zone contains relatively low numbers of proteins (ideally only one), analysis of the resulting digests often relies on matrix-assisted laser desorption-ionization—time of flight (MALDI-TOF)- or TOF/TOF-MS without introduction of any chromatographic separation step. In the simplest case, MALDI-TOF-MS analysis employs so-called peptide mass fingerprinting, i.e., protein identification by the characteristic MS pattern of proteolytic peptides [145,146]. This approach suffers, however from poor sensitivity and selectivity [56]. Indeed, only highly-abundant proteins generate patterns of proteolytic peptides sufficient for their unambiguous identification. On the other hand, these abundant components suppress the signals of co-migrating peptide ions representing minor proteins, making their annotation challenging. To some extent, this limitation can be overcome by tandem mass spectrometry (MS/MS): MALDI-TOF/TOF instrumentation can be applied to obtain more specific and reliable results [140,147]. However, because of poor precision of precursor isolation (usually relying on time-based selection during separation in the first short field-free region with the precision of not better than ±5 *m/z*), for MALDI-TOF/TOF-MS instruments, identification by several such peptides might be advantageous. Indeed, as it was shown in 2D-GE-MALDI-TOF/TOF-MS experiments with mixtures of six proteins, MS/MS-based identification with three peptides seems to be more reliable [148], that would be in agreement with standards, conventionally accepted in liquid-chromatography-based animal [149] and plant [63] proteomics. In practice, this identification scheme can be approached by the implementation of reversed phase—high performance liquid chromatography (RP-HPLC) separation in off-line mode and/or automated acquisition protocols, relying on MS/MS analysis of multiple selected precursor ions—a strategy, well-applicable to plant proteomics [150]. In general, it needs to be concluded, that the low resolution of the precursor selection algorithm essentially restricts the discovery potential of MALDI-TOF/TOF-MS. Implementation of liquid chromatography (LC) separation prior to MALDI-TOF/TOF-MS analysis allows overcoming this limitation and provides improved identification rates for proteins, co-migrating in one electrophoretic zone (spot) [151]. It is important to note, that in some cases, for example in analysis of seed storage proteins (SSPs), this strategy might not be efficient enough for distinguishing closely related polypeptides. Indeed, such proteins have a high degree of homology in their sequences because they are encoded by paralogous genes. In such cases considering specific signal patterns at 2D-GE protein maps can be helpful for correct protein annotation.

One of the most important features of 2D-GE is the possibility for relative quantification of individual electrophoretic zones [152]. This information provides direct access to quantitative profiles of protein abundance (also often referred to as protein expression) and allows clear and straightforward statistical interpretation of data [153]. In this context, protein dynamics, i.e., alterations in protein expression profiles in time or in response to application of certain experimental conditions, can be characterized [23,140]. Thereby, UV or VIS optical density of stained proteins, or intensity of fluorescence signal, can be considered as quantitative parameters. The appropriate integration algorithms are usually implemented in automatized protocols and established software tools, providing convenient access to image acquisition, spot detection, matching, normalization, statistical analysis, and annotation, also often combined with robotized spot cutting and in-gel digestion. The most commonly applied tools rely on numerical approaches (PDQuest (Bio-Rad Laboratories), Delta2D (Decodon), and Melanie (GeneBio) and can be successfully complemented by home-made systems [154].

Unfortunately, although 2D-GE instrumentation is relatively cheap, easy to handle and adequately supported by efficient data analysis software, this method has several intrinsic limitations. The most critical of them is the relatively low inter-gel reproducibility, i.e., difficulties in spot detection and matching in parallel gels [155]. Indeed, each IPG strip and electrophoresis block represents an independent system with separation conditions, different from those of others. Although batch setups for casting and running gels are currently well-established, this inter-system error cannot be eliminated completely because of a local heterogeneity of casting solutions. This fact might also affect dispersion within treatment groups and significance of inter-group comparisons [156]. Fortunately, this essential limitation can be efficiently overcome and adequately corrected by sample multiplexing in terms of the difference gel electrophoresis (DIGE) approach [157]. For this, cyanine dyes (Cy3 and Cy5) can be added to alternating samples, whereas the third dye (Cy2) can be used for standardization [158]. Due to the presence of different fluorophores, each dye can be detected independently without essential cross-talk with limits of detection (LODs) in the higher pg range. Therefore, this method was successfully applied in seed proteomics [20,28].

Although the 2D-GE technique is being continuously improved, some limitations of the method have not yet been overcome. First, as most of the spots are composed of several proteins (typically present in unknown ratios), the reliability of quantitative profiles obtained by 2D-GE is often questionable. Because of this, additional quantitative Western blotting experiments for proteins of interest are desired [31]. Further, even when multiplexing with DIGE is performed, the method might still suffer from inter-replicate variability. This could be overcome by increasing the numbers of technical replicates, that would make experiments more laborious and dependent on personnel performance. Finally, in comparison to gel-free techniques, 2D-GE has lower analytical resolution and maximal achievable linear dynamic range (which for modern LC-MS-based techniques can exceed five orders of magnitude [159]). Whereas analytical resolution of gel-based methods can be essentially increased by sample pre-fractionation (either at the step of sample preparation or isoelectrofocusing), these methods are time-consuming. Therefore, gel-free techniques are being actively introduced into seed proteomics, though one must keep in mind, that due to principally different separation mechanisms, some data—molecular weight and isoelectric point, easily deliverable by 2D-GE, are not accessible by gel-free techniques. It makes 2D-GE-MS identifications quite reliable and useful for discrimination of false-positives generated by the gel-free approach.

## 4. Gel-Free Bottom-Up Proteomics

The gel-free approach assumes the application of enzymatic proteolysis prior to separation, i.e., the entire protein extract is digested, and the resulting mixture of proteolytic peptides is separated (Figure 1). The analysis typically employs nanoRP-HPLC with electrospray ionization (ESI)-MS coupled on-line [56] or with matrix-assisted laser desorption/ionization (MALDI)-MS coupled off-line [160]. Among these two techniques, LC-ESI-MS is more powerful and may result in identification of up to several hundred thousand peptides [11]. Thus, the LC-MS approach provides impressive analytical resolution and represents a powerful tool, delivering protein identification rates approximately ten-fold higher than conventional 2D-GE methods [161]. In plant science, specifically in seed research, gel-free bottom-up proteomics studies are accomplished as so-called shotgun experiments, assuming comprehensive characterization of complex samples in one LC-MS experiment [162].

Unfortunately, despite of its high analytical power, LC-MS-based proteomics has some limitations that need to be kept in mind when planning experiments and interpreting their results. Thus, in contrast to gel-based ones, this technique is highly sensitive to matrix effects, which are typically manifested as suppression of low-intensity peptide signals by highly abundant co-eluting species [163,164], that might compromise dynamic range of proteome analysis and precision of quantification. Therefore, the separation efficiency of the LC-MS system, prefacing ESI-MS is of critical importance for successful identification of low-abundant peptide quasi-molecular ions and quantification of corresponding proteins.

Another issue is the well-known incompatibility of the ESI mechanism with detergents (e.g., SDS and CHAPS) [165]. As desolvation of low molecular weight analyte ions relies on the solvent evaporation model [166], and detergents suppress evaporation of eluents in droplets, these compounds disrupt the transfer of analytes into the gas phase and dramatically affect the sensitivity of detection. Besides this, conventional detergents (like SDS, Triton X100, and CHAPS) are quantitatively retained in reversed phase chromatography (RPC) and broadly co-elute with proteolytic peptides, further suppressing their signals [167]. Although SDS can be removed by potassium dodecyl sulfate (KDS) precipitation, this procedure requires time-consuming optimization (Table A2) [168] and is therefore still not established for plant material. Thus, the mentioned detergents cannot be employed in sample preparation for LC-MS and isolation of proteins with phenol- and detergent-free aqueous buffers gives access only to the soluble part of the plant proteome [169]. In this case, pre-cleaning of protein extract might be necessary to remove low molecular weight metabolites, which may interfere with enzymatic proteolysis (e.g., by inhibition of proteases). Thereby, preparation of plant aqueous protein extracts requires more purification effort, in comparison, for example, with analogous isolations from mammalian tissues. Indeed, whereas for human blood plasma samples ultrafiltration is sufficient to remove interfering metabolites and salts [170], for plant extracts an additional pre-cleaning by size-exclusion chromatography on PD-10 columns is required [171]. Isolation of seed proteins by this method might be even more challenging: unfortunately, the major storage proteins tend to aggregate in the absence of detergents [93], that might result in the loss of associated low-abundant proteins as well. Therefore, this isolation strategy is hardly applicable in seed proteomics and is generally not recommended. Therefore, the introduction of SDS-PAGE as a sample preparation step can be a good alternative. In terms of this setup, the isolated protein mixture is reconstituted in SDS-sample buffer and separated by SDS-PAGE (Table A2). Afterwards, the whole lanes are excised and cut into several (at least ten) segments for in-gel digestion with protease. After pooling of individual digests, samples can be desalted and analyzed by LC-MS. This protocol proved to be well-applicable to protein-rich legume seeds [108]. However, both of the described strategies have two essential disadvantages: (i) they require time-consuming sample preparation, and (ii) might suffer from the incomplete recovery of the seed proteome. Fortunately, recovery of in-gel digestion can be potentially increased by replacing bis-acrylamide with its disulfide analog—bis-acrylcistamine (BAC) [172]. Treatment of such gels with tris(2-carboxyethyl)phosphin (TCEP) might provide quantitative extraction of proteolytic peptides after digestion. Alternatively, digestion recovery in experiments with seed proteins can be improved when detergent-free buffers containing urea or guanidine chloride are applied [95]. However, despite of this improvement, in the absence of detergents, supplementation of extraction buffers with chaotropic agents does not provide the highest possible proteome coverage.

Importantly, the number of identified proteins can be affected by intrinsic limitations of data-dependent acquisition (DDA) experiments, which are most widely used in LC-MS proteomics [173]. Thus, state of the art quadrupole-orbital trap (Q-Orbitrap), Orbitrap-based tribrids, and quadrupole-time of flight (QqTOF) mass spectrometers are able to perform DDA experiments that comprise up to 20 dependent MS/MS scans, acquired for the most intense signals detected in a survey full-MS scan within a cycle of 0.5–1 s [174,175]. However, despite this high acquisition speed (scan rate up to 20 Hz), the number of peptides that can be sequenced in an MS/MS scan is still limited and usually essentially lower than the number of multi-charged features detected at the MS level. It is especially important in seed proteomics, as seed proteome is strongly dominated by a few major storage proteins, which not only suppress identification of minor polypeptides [63,69] but also involved in the formation of amyloid structures [176]. To some extent, these problems can be addressed by introduction of pre-fractionation steps and method-specific exclusion lists [177]. However, from today’s perspective, data-independent acquisition (DIA)-based techniques, ideally in combination with DDA seem to be the best choice [178], especially when post-translational modifications are addressed [179].

Another important issue is the relatively low inter-run reproducibility of spray performance. Because of this, intra- and inter-batch precision of label-free quantification is typically low. Therefore, analytical strategies need to employ inter- and intra-batch normalization [180]. In this context, techniques relying on internal normalization and multiplexing, like metabolic [181], chemical [182,183], and stable isotope/^18^O [184] labeling are advantageous when precise quantification is desired. Although these methods have already become common in plant proteomics, they are still only applied to green parts (mostly leaf) of the most well-characterized model plants, like *Arabidopsis*. Thus, their use in seed research is still quite limited and uncommon. Finally, probably the most important factors limiting the application of LC-MS-based methods are the high costs of instrumentation and limited availability of well-trained personnel. Due to this and the above-mentioned advantages, 2D-GE remains the main “working horse” in seed proteomics.

Fortunately, current developments in analytical science provide adequate solutions to all (probably, besides the last one mentioned) limitations of the gel-free approach. Typically, these developments were initially introduced in cell, blood plasma, or mammalian tissue proteomics, and so far only a few of these methodological solutions are transferred to plant and seed research. Thus, the most of these improvements were implemented quite recently and mostly in the fields, not related to plant and, especially, seed research. Therefore, with this review, we would like to bring this summarized and critically discussed information to the seed proteomics society with the hope to push their implementation in plant proteomics. In this context, we are convinced that many of these recently proposed method improvements might allow versatile shotgun LC-MS analyses of the total seed proteome in future.

As the first improvement, highly efficient separation can be achieved by nano-scaled ultra-high performance liquid chromatography (UHPLC) [56]. Elution of individual analytes in well-defined chromatographic zones minimizes matrix effects and attenuates suppression of low-intense signals by highly-abundant species [185]. The quantitative reconstitution of membrane proteins without any deleterious effects on the sensitivity of peptide detection is another essential achievement. Thus, at the end of the last decade, Mann and co-workers proposed the so-called filter-aided sample preparation (FASP) method, assuming digestion of proteins in the presence of SDS on membrane filters, and exchanging it by urea afterwards (Table A2) [186]. This technique was successfully transferred to plant and, specifically, to seed biology: it proved to be efficient for digestion of grape leaf [187], cucumber seed proteins [188], and delivered superior protein identification rates in comparison to sample pre-cleaning by SDS-PAGE. Recently, Bose et al. comprehensively compared the performance of FASP and different extraction buffers with seeds of four cereal species [64] and demonstrated `broad applicability of this technique in seed biology.

An alternative approach relies on immobilization of protein in gel by supplementing with acrylamide/bisacrylamide mixture after its complete solubilization in the appropriate detergent solution (so-called tube-gel digestion protocol) [189]. Immobilization in gel results in unfolding of hydrophobic (e.g., membrane) proteins and promotes their effective digestion. A similar protocol, known as gel-aided sample preparation (GASP), employing immobilization of proteins in the gel, is also applicable to intact cells [190]. These methods are well-suited for the identification of membrane and membrane-bound protein complexes, and GASP protocol was recently applied to the analysis of *Nicotiana benthamiana* leaf proteins [191]. Both FASP and GASP yield up to five-fold higher intensities of peptide signals in comparison to in-solution digestion in absence of detergents. Interestingly, the application of aqueous solutions containing high proportions of acetonitrile results in superior digestion yields when small protein amounts need to be hydrolyzed [192]. Surprisingly, the number of identified peptides was much lower when methanol was used [29]. Recently, the GASP technique was successfully up-scaled to a 96-well format—so-called HiT Gel (High Throughput in Gel Digestion) protocol, successfully applied to *Arabidopsis thaliana* protein extracts [141]. However, this technique has not yet been transferred to seed proteomics.

Further improvement of proteome coverage could be achieved by the application of commercially available detergents, forming insoluble precipitates under low pH values [193]. One such compound, sodium deoxycholate (SDC), is well-known from blood plasma proteomics [170]. Recently, SDC was successfully applied to solubilization and digestion of total barley leaf protein preparation [194] and isolates from germinating maize seeds [195]. Despite of its wide application, precipitation of SDC upon digestion is not always quantitative. The same is the case for sodium laurate [196].

An alternative solubilization/digestion strategy might rely on degradable acid-labile detergents. For example, zwitterionic PPS Silent Surfactant [197] and anionic Protease-MAX [198] surfactants are a good alternative for SDS in digestion buffers. To date, these detergents have only been used rarely in plant proteomics. Thus, Protease-MAX was recently successfully used for the characterization of *Solanum tuberosum* leaf tissue [199], whereas the former detergent, to the best of our knowledge, was not applied to analysis of plant tissues so far. Both these chemicals have not been used yet in seed research. It is important to note that PPS Silent Surfactant and Protease-MAX reagents yield hydrophobic cleavage products, which form films on the surface of aqueous phases and contaminate samples [200]. This ultimately requires additional intensive sample cleaning after proteolysis. The same is known for the anionic acid-labile detergent RapiGest SF Surfactant [201], which is currently most often applied for digestion of human plasma and tissues [202]. Recently, this reagent was successfully employed in the digestion of barley seed protein isolates, resulting in the annotation of 226 polypeptides in shotgun data-independent experiments [203]. Its application to quinoa seeds gave access to new lysine-rich seed storage globulins [162].

Another option available from the company Progenta is anionic, cationic, and zwitterionic acid-labile surfactants I and II (AALS I/II, CALS I/II, and ZALSI/II), among which AALS II, CALS II, and ZALSII have higher protein solubilization potential, i.e., can be applied to hydrophobic (e.g., membrane) proteins [204]. Recently, in a systematic comparison of different degradable surfactants, AALS II (structurally mimicking SDS) showed the best performance in terms of solubilization efficiency and sequence coverage of digested and analyzed standard protein samples [205]. This detergent was successfully optimized for the digestion of protein isolates obtained from seeds of different species [26,55,63]. Importantly, cleavage of this detergent yields relatively hydrophilic products, which can be easily removed from seed protein hydrolysates by solid-phase extraction (SPE) on the reversed-phase [26]. This compound has tunable surfactant properties, i.e., adjustable critical micelle concentration (CMC) [200]. Therefore, recently we selected this surfactant for profiling the total *Arabidopsis* leaf proteome [177,206]. Later, we successfully addressed proteome changes in oilseed rape (*Brassica napus*) seeds during germination [26] and mature pea seeds [55,63].

Remarkably, when we combined application of AALS II with long chromatographic gradients of 1.5–3 h (which allow higher numbers of cycles of DDA experiments per acquisition run), confident identification of thousands of proteins could be achieved not only in *Arabidopsis* leaf tissues [177] but also in pea seed embryos [63]. The effect of the under-sampling phenomenon, known to accompany DDA experiments [173], can be further attenuated by sample enrichment, depletion, or pre-fractionation. For example, removal of legume reserve seed proteins by their precipitation in presence of protamine sulfate or by organic solvents represents a well-established depletion procedure, which provides a deeper insight into the seed proteome [68]. For specific enrichment of seed glyco- and phosphoproteome, adequate protocols were successfully established [207,208]. Other fractionation techniques, like pre-fractionation by hydrophilic interaction liquid chromatography (HILIC) [177], gas-phase fractionation (GPF) at the level of mass analyzer [209], or selective enrichment of glycated proteolytic peptides by boronic acid chromatography (BAC) [171,210] are already established in plant biology and can be easily transferred to the specific field of seed biology. In general, fractionation, enrichment, and depletion procedures dramatically reduce the complexity of peptide mixtures analyzed in individual fractions and thus ensure the complete detection of low-abundant proteins. After combining the results obtained in all experiments, substantial improvement of proteome coverage could be observed.

**Figure 3:**
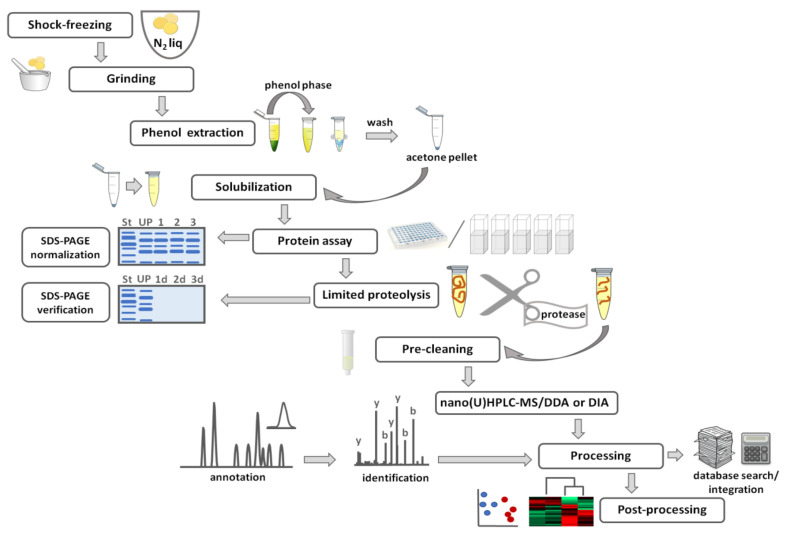
Detailed workflow for LC-based proteomics: protein isolation, sample preparation, and analysis.

To achieve the highest possible protein identification rates and precision of label-free quantification, we optimized our proteomics workflow for the highest protein recovery and reproducibility (Figure 3). Thus, in terms of our approach, after shock-freezing in liquid nitrogen, seeds or their parts (e.g., embryos or cotyledons) are ground in a ball mill, and 50–200 mg (depending on species) of frozen powder are taken for phenol extraction [26,89]. After drying, protein isolates are quantitatively reconstituted in the shotgun buffer, containing chaotropic agents (urea and thiourea) and degradable detergent (Progenta Anionic Acid-Labile Surfactant, AALSII) [206]. If protein isolation is performed carefully, and no interphase (typically containing nucleic acids and polysaccharides) is co-precipitated with the target protein fraction, seed protein isolates can be reconstituted completely. If it is not the case, after short centrifugation, protein contents are determined in supernatants. For this, we are using a two-step procedure, relying on protein determination by the 2D-Quant kit (which is based on specific binding of copper to protein [211]) with subsequent cross-validation of results by SDS-PAGE with Coomassie staining according to a well-established protocol [212]. The application of these two orthogonal approaches ensures reliable normalization of protein amounts taken for enzymatic digestion. According to our experience, twice repeated incubation of solubilized proteins with trypsin was sufficient for complete proteolysis of seed samples [56]. The completeness of hydrolysis was confirmed by SDS-PAGE, as was earlier established for human samples [107] and successfully transferred to plant, and specifically seed samples. Having this in hand, AALS can be destroyed, and the digests can be pre-cleaned with RP-SPE either in a cartridge or in a stage-tip format [59,153].

The pre-cleaned samples can be analyzed by nano (U)HPLC-ESI-MS employing either data-independent acquisition (DIA) or data-dependent acquisition (DDA) modes (Figure 3). In QqTOF-MS instruments, DIA relies on the simultaneous fragmentation of multiple peptide ions within a wide range of *m/z*. In its simplest form, this *m/z* range covers the whole measurement mass range (MS^E^ technology, employed by Waters instruments) [213]. This approach proved to be efficient in discovering seed proteins [112,214]. Alternatively, the full mass range can be split into 10–30 segments or windows of 20–50 *m/z* each wherein all ions are concomitantly fragmented—the so-called sequential window acquisition of all theoretical fragment ion spectra (SWATH) MS technology [215]. The strategy for analysis of SWATH data is based on peptide-centric scoring, which relies on querying chromatographic and mass spectrometric coordinates of the proteins and peptides of interest in the form of so-called peptide query parameters (PQPs) [216]. The SWATH-based DIA approach allows essential coverage of seed proteome and identification of more than 2000 individual proteins in one experiment [217]. In contrast, the DDA scan strategy relies on selective fragmentation of a defined number of the highest intensity signals detected in survey full-MS scans [173]. To increase analytical reproducibility, this approach can be additionally mapped to extracted ion chromatogram (XIC) information [218]. Identification of individual peptide sequences relies on tandem mass spectrometry (MS/MS, Figure 3) whereas quantitative information can be derived from spectrum counting (PSM counting), peak heights, and, in the most precise way, from the integration of extracted ion chromatograms [219]. During the recent decade, this approach remains a working horse of gel-free proteomics [55,220,221].

Unfortunately, both DIA and DDA have advantages and disadvantages, which need to be considered in each specific case when developing an analytical strategy. Thus, DIA delivers rich MS/MS information, although the correct annotation of fragments is critical for this technique. To some extent, targeted extraction of MS/MS spectra might simplify this task [216]. On the other hand, the major challenge of DDA experiments is analytical under-sampling [173]. Indeed, only a limited number of peptide ions can be fragmented in each DDA method cycle, whereas the major part of the proteome remains unidentified. To minimize this limitation, LC-MS analysis can be pre-faced with an additional chromatographic dimension, or implementation of prolonged separation gradients is required [177]. Thereby, the selection of mass spectrometric instrumentation in each case is generally governed by the overall goal of the specific research.

In this context, high resolution and high mass accuracy are critical for successful annotation of sequence tags in discovery proteomics. In the simplest case, these requirements are fulfilled only at the MS level. The technical solution for this is the linear ion trap (LIT)-Orbitrap-MS technology, which employs low-frequency orbital traps and long scan times in FT mode. This assumes long MS scans with high (up to 120,000) resolution and mass accuracy about 1–3 ppm, whereas the survey MS/MS scans are acquired with unit resolution [222]. In contrast quadrupole-orbital trap, (Q-Orbitrap) instruments (Q-Exactive and Exploris™ instruments from Thermo Fisher Scientific), orbital trap-based tribrids (Fusion, Lumos und Eclipse mass spectrometers from the same company), and QqTOF mass analyzers rely on high resolution and high mass accuracy both in MS and MS/MS mode [223]. Therefore, for DDA experiments, these instruments represent the best option and give access to higher identification rates in comparison to LIT-Orbitrap-MS [173,224]. Thus, they are widely and successfully applied in seed proteome research, yielding the most comprehensive sequence coverage and giving access to the most representative seed protein datasets. 

On the other hand, for targeted proteomics, triple quadrupole (QqQ) mass spectrometers, operating in selected reaction monitoring (SRM) mode, provide the highest sensitivity in the sub-ng/mL range [225]. As QqTOF-MS, from the hardware side, can be considered an extension of QqQ-MS, these instruments are also well suited for analysis in SRM mode [226]. A scan strategy called parallel reaction monitoring (PRM) extends the targeted SRM approach to the Q-Orbitrap mainly because of its two linear quadrupoles configured before the Orbitrap mass analyzer by analogy to the triple quadrupole configuration. It acquires MS/MS spectra of precursor ions isolated in the second quadrupole mass filter as opposed to monitoring individual transitions as it can be accomplished in the selected/multiple reaction monitoring (SRM/MRM) mode, delivering higher specificity with comparable sensitivity [227]. Additionally, it circumvents many of the difficulties associated with SRM development, such as optimization of collision energy and dwell times. That approach has also been demonstrated on the LTQ-Orbitrap with a limit of quantification of 100 amol in a complex plant leaf extract [175].

Although relatively few (in comparison to gel-based) gel-free seed proteomics studies have been published so far, LC-MS based techniques essentially increased the power of plant proteomics in general [57] and the depth of seed proteome characterization in particular [46,63]. Indeed, the first proteomic maps were based on two-dimensional gel electrophoresis (2D-GE) and mass spectrometric (MS) identification of visualized electrophoretic zones (spots) and contained hundreds of proteins [34]. The majority of the identified polypeptides were storage proteins, which strongly dominate the seed proteome and can serve as seed protein quality markers [66]. Removal of these highly abundant storage proteins by isopropanol- [67] or by protamine sulfate-containing solutions [68] could increase coverage of the seed proteome, however, the number of identified proteins never exceeded several hundred. Implementation of liquid chromatography (LC)-MS-based strategies provided a deeper insight into the seed proteome [20,35,64]. Growing numbers of gel-free proteomics studies extend our knowledge providing more detailed information about the seed proteome (abundant and non-abundant proteins, PTMs) in a diversity of plants: barley, rape, wheat, rye, oats, soybeans, pea, etc [26,63,64,203,228]. Thereby depletion of storage proteins and enrichment of low-abundant post-translationally modified species is desired. Indeed, the most representative LC-MS-based study to date of Min et al. performed with soybean seeds, identified 1626 non-redundant proteins [54]. Finally, this year Mergner et al. reported a comprehensive identification of Arabidopsis sees proteins: the authors reported more than ten thousand seed proteins, as a part of the most recent proteomics atlas [229]. This number significantly exceeds the outcomes of gel-based proteomics. Recently we reported the most complete, to the best of our knowledge, legume seed proteome map, comprising about 2000 non-redundant proteins. We compared seed proteomes of yellow- and green-seeded pea cultivars in a comprehensive case study. The analysis revealed a total of 1938 and 1989 non-redundant proteins, respectively [63].

It is important to note that, despite its high efficiency, the gel-free approach has several bottle necks. First of all, it requires expensive instrumentation, highly-competent personnel, and implementation of powerful bioinformatic workflows. Another serious problem is the high complexity of plant matrices, which might result in strong matrix effects, compromising the accuracy of quantification. Therefore, although the linear quantitative dynamic range of gel-based techniques is at least two orders of magnitude lower in comparison to state of the art LC-MS methods, in many cases, 2D-GE might provide more reliable quantification. Therefore, complementation of the gel-free proteomics strategy with the gel-based techniques is always beneficial and gives the best results [140]. Indeed, this complementation may include several important aspects and essentially impact on data reliability due to cross-validation of results. First, simultaneous considering data sets obtained with 2D-GE and nano-LC-MS results in higher protein identification rates and more comprehensive proteome coverage [33,57,230]. Importantly, in contrast to LC-MS, 2D-GE delivers important information on protein molecular weights and isoelectric points [231]. This feature allows direct verification of shotgun proteomics results. Additionally, this method gives information on patterns of isoforms with their post-translational modifications, which can be easily visualized by specific staining [77].

## 5. Post-Translational Modifications

Despite the importance of data on changes in protein abundance, this information is insufficient for a complete understanding of regulatory events behind seed maturation and germination [11]. Indeed, enzymatic post-translational modifications (PTMs), such as phosphorylation, acetylation, and glycosylation, have a great impact on intracellular signal transduction pathways [232]. Accordingly, knowledge of patterns of regulatory enzymatic PTMs facilitates understanding of biochemical and physiological alterations accompanying seed development, maturation, germination, and ageing [233]. Therefore, a comprehensive analysis of specific modification sites in seed proteins represents an important direction of actual research.

Among post-translational covalent PTMs, reversible protein phosphorylation stands out as the most extensively studied one and essentially contributes to the regulatory network, switching multiple critical cellular functions between the active and inactive state [234]. In this context, knowledge about the changes in the phosphorylation status of individual seed proteins during maturation, germination, and early seedling development might be a good starting point to study their contribution into underlying regulatory events, giving a deep insight into metabolic control of corresponding functions [47]. It is important to note that the phosphorylation state of each individual protein depends on activities of multiple enzymes—kinases and phosphatases, as well as phosphate-binding proteins, which catalyze phosphorylation and dephosphorylation, respectively, at one or several sites [235]. In the last decade, analysis of phosphorylation sites became a powerful tool to address regulatory aspects of seed development and germination [236]. Recently, Meyer et al. reported more than one thousand phosphorylated sites in seed proteins of *Arabidopsis thaliana*, soybean, and rapeseed, and pinpointed RNA biosynthesis and metabolism as the most affected gene ontology categories [47].

In the simplest and most straightforward case, the phosphorylation status of proteins can be derived from 2D-GE experiments, using specific phosphostaining. Indeed, phosphorylation typically affects protein pI values and results, thereby, in the appearance of specific signals for individual phospho isoforms in 2D-GE electropherograms [237]. This approach proved to be the method of choice for the characterization of phosphorylated proteins among seed storage polypeptides—cruciferins, napins, cupins, and vicilins [47,238,239,240,241]. Thereby, phosphorylated isoforms can be quantified by chemical dephosphorylation with hydrogen fluoride-pyridine (HF-P) [242,243,244], i.e., phosphorylation levels can be assessed by the difference in spot intensities, observed in gels, run before and after application of HF-P [245]. Most often, detection or cross-validation of such protein phosphorylation patterns relies on Pro-Q diamond phosphoprotein stain (Pro-Q-DPS), which is known to be a rapid and convenient tool for in-gel detection, mapping, and quantification of polypeptides [246,247]. For example, this method was successfully applied to the characterization of differential phosphorylation patterns accompanying grain development in two different Chinese bread wheat cultivars [248]. Further, this staining protocol was employed in phosphoproteome analysis of germinating seeds and early grown seedlings from *Quercus ilex* L [128]. Implementation of this protocol in parallel to a standard protein quantification procedure (e.g., Coomassie blue, silver, or Sypro Rubi staining) might give direct access to the proteins, whose abundance is regulated via phosphorylation [246]. Moreover, as was shown by Han et al. in their study of rice embryo proteome dynamics during seed germination [249], an extension of this approach on enzyme activity assays allows direct consideration of such regulatory events in the context of accompanying metabolic alterations. Despite its ease in handling and good performance, gel phosphoprotein stains have an important limitation: this method does not allow reliable assessment of phosphorylation status at specific residues when several phosphosites are present in the sequence. Spot excision followed with proteolysis, and MS/MS analysis typically gives access to protein identification, but not localization of phosphorylation sites. In such cases, site-specific antibodies or/and gel-free MS/MS-based techniques [250] need to be applied. Despite of this, the potential of the phosphostaining techniques should not be underestimated. For example, this approach is useful for fast screening of phosphoproteomes isolated from different species or tissues [251].

In contrast to the gel-based phosphostaining strategy, quantification of individual phosphorylation sites gives access to the dynamics of phosphorylation at individual protein residues in plant proteins. In this way, activation or inhibition of specific kinases and/or phosphorylases can be directly addressed either on a relative or absolute basis [252]. Quantitative assessment of these alterations in enzyme activities typically relies on corresponding proteolytic peptides obtained after enzymatic digestion of cellular proteins and enriched by immobilized metal affinity chromatography (IMAC) or metal oxide affinity chromatography (MOAC) [222]. The latter technique is currently recognized as the “working horse” of phosphoproteomics, and in the absolute majority of cases relies on titanium dioxide (TiO_2_) affinity chromatography [253], although the stationary phase can be represented by zirconia (salts of Zr^4+^) [254], aluminum hydroxide [255] and iron oxide [222] as well. Recently, Ni-functionalized particles [256] and iron (III) stearate Langmuir-Blodgett films [222] were proposed as affinity materials for IMAC, also these techniques are still to be implemented in plant and seed biology. Pre-fractionation of complex protein digests might be another approach, giving access to enhanced phosphopeptide detection. Thus, implementation of strong cation exchange chromatography (SCX), electrostatic repulsion hydrophilic interaction chromatography (ERLIC), and solution isoelectric focusing (sIEF) resulted in enhancement of phosphosite identification [257]. However, despite of their high analytical potential, these methods still need to be optimized for seed research. Regardless of their origin, the obtained eluates or fractions are typically purified by solid-phase extraction and analyzed with nano(U)HPLC-MS and MS/MS [258]. Thereby, these experiments typically rely on label-free techniques, i.e., direct analysis of individual samples without multiplication [259]. However, due to the intrinsic limitation of ESI, all currently available label-free quantification (LFQ) techniques typically suffer from compromised precision and high inter-sample variability [219]. Although appropriate standardization and normalization procedures can improve data quality, this still remains a bottleneck of LFQ analysis [180].

To overcome this limitation, powerful chromatographic techniques can be efficiently complemented with ^15^N labeling or derivatization-based techniques—tandem mass tags (TMT) or isobaric tags for relative and absolute quantification (iTRAQ) [260]. The first strategy represents an in vivo labeling technique, often also referred to as *Arabidopsis* metabolic labeling [261], functionally analogous to SILAC (stable isotope labeling of amino acids in cell culture). SILAC is commonly used in animal and human cell biology and gives a unique opportunity for fine dissection of regulatory pathways by simultaneous measurement of samples of various biological scenarios and deriving differences in phosphorylation directly from the mass spectra [235]. *Arabidopsis* metabolic labeling, relying on aqueous [262] or solid [263] media allows doing the same at the level of plant organism [264], that is hardly possible even for basic model animals. Although in vivo metabolic labeling was applied to *Arabidopsis* seedlings [265], this method still was not used in seed research. To a large extent, it can be explained by the high costs of ^15^N-labeled salts and the long time required to obtain seeds, especially for valuable crop plants, like legumes or cereals. However, despite these high costs in vivo labeling of seed phosphoproteome might be a promising direction of new studies.

Techniques based on chemical tags, like iTRAQ and TMT, rely on post-isolation derivatization with multiplexing differentially isotopically labeled agents, subsequent purification with cation exchange chromatography, and nanoRP-(U)HPLC [266]. The quantitative analysis relies on tag-specific signals in the low-*m/z* range of tandem mass spectra, acquired with combined samples [267]. The iTRAQ approach proved to be efficient in analysis of plant proteome [268] and was successfully introduced in seed research. Thus, Zhang et.al. analyzed the regulatory pathways behind dormancy and germination of grassbur twin seeds and proved the regulation of ribosome synthesis and carbohydrate metabolism during seed germination via the phosphoinositide 3-kinase (PI3K) pathway [269]. Moothoo-Padayachie et al. used iTRAQ to explain the mechanisms behind the viability loss upon desiccation of recalcitrant *T. dregeana* seeds [270]. Interestingly, despite its convenience and reliability, TMT still was not used in seed phosphoproteomics.

Another important PTM is glycosylation, which patterns in plants differ significantly from mammals [271]. Besides its known role in the regulation of cell functions and compartmentalization, this modification is believed to impact seed allergenicity, attracting the special attention of food scientists due to a concept of carbohydrate cross-reactive determinants (CCDs) [272]. Seed storage proteins are known to be the most abundant polypeptide fraction, demonstrating a high degree of glycosylation [237]. As glycosylation affects protein molecular weights and isoelectric points, characteristic patterns of differentially glycosylated isoforms can be efficiently characterized by 2D-GE [79]. Individual electrophoretic zones (spots) related to glycosylation can be identified by comparison of electropherograms acquired before and after deglycosylation with protein-N-glycosidase (PNGase), as was shown by de La Fuente et al. for phaseolin patterns in common bean (*Phaseolus vulgaris* L.) [273]. The relative levels of each glycosylated isoform can be quantified according to the data processing strategy described by Bernal et al. for mapping of patatin isoforms [245] or using similar processing pipelines. Alternatively, glycosylated proteins can be stained directly in-gel. This can be accomplished by the application of a fluorescent hydrazide Pro-Q Emerald 300 dye, available as a Glycoprotein Gel and Blot Stain Kit [274]. The glycoprotein bands or spots can be visualized by fluorescence detection after UV transillumination at 300 nm. Another glycoprotein quantification approach relies on the electrophoretic transfer of the proteins, separated by 2D-GE, to a polyvinylidene difluoride (PVDF)-membrane with subsequent visualization with a concanavalin A (Con A)/horseradish peroxidase (HRP) method [275], as was done by Weiss et al. for fractionated proteome of barley seeds [276]. The interaction of the lectin Con A with glycoproteins can be also employed for affinity chromatography, typically accomplished with agarose-immobilized Con A as a stationary phase, packed in tubes, columns, or grafted on more unconventional solid surfaces like colloidal gold and affinity membranes [277]. This technique can be combined with further analytical methods to probe specific glycoprotein interactomes or enzymatic activities. For example, Ostrowski et al. incubated pea seed glycoproteins with [^14^C]-indole-3-acetic acid ([^14^C]-IAA) in the presence of 1-*O*-indole-3-acetyl-β-*D*-glucose (IAGlc) synthase showed that this enzyme impacts on modification of glycosylated proteins with IAA [278]. To address the composition of protein-bound glycans, the oligosaccharide moieties can be cleaved from the polypeptide chains by PNGase, chemically derivatized with a fluorescent tag, and analyzed by hydrophilic interaction liquid chromatography (HILIC) with fluorescent detection, whereas the structure of the glycan moiety can be assessed by MALDI-TOF/TOF-MS [279].

Several other enzymatic PTMs are in the focus of seed biology research due to their involvement in regulation of key physiological processes accompanying seed maturation and germination. Thus, acetylation and succinylation are the PTMs, mainly involved in regulation of metabolism [280]. Recently, the Miernyk’s group established a two-level immunoaffinity enrichment procedure for acetylated proteins and peptides for the study of the soybean seed acetylated proteome in a combination of storage protein depletion and linear ion trap (LIT)-Orbitrap-MS [281]. The combination of these techniques allowed identification of 245 acetylated proteins in developing soybean seeds. Later, He et al. applied specific immunoaffinity enrichment of seed protein tryptic digests followed with high resolution (HR)-nanoLC-MS and MS/MS for comprehensive profiling of acetylated and succinylated rice seed proteome that indicated the involvement of these modifications in multiple functions and strong physiological cross-talk between these two PTMs [282]. Another important modification in seed proteins is lipidation (most often—myristoylation, palmitoylation, and prenylation), which is often highly relevant for the interaction of dehydrins with membranes [283,284]. These modifications were detected in the dehydrin/abscisic acid (ABA)-responsive protein of *Fagus silvaticus* seeds by the combination of computational and molecular biology methods [285]. Methylation of K and R residues is widely spread in the plant kingdom [286], although it is clearly under-characterized in seeds. To summarize, among enzymatic modifications, phosphorylation and glycosylation are relatively well studied, whereas the data on acetylation of seed proteins are currently being actively acquired. In contrast, other enzyme-dependent modifications are still to be completely characterized in seed proteome.

## 6. Data Processing and Post-Processing

Large datasets generated in high-throughput proteomic experiments require powerful computational platforms, which enable reliable annotation of MS and MS/MS signal patterns to sequences of specific proteolytic peptides, give access to precise quantitative assignments, and support further statistical analysis to retrieve biological information. Here we summarize some major recent achievements, facilitating efficient processing and post-processing of proteomics data.

In MS-based proteomics, identification of peptide sequences typically relies on so-called sequence tags (see above) [287]. In the simplest form, a sequence tag might contain only patterns of peptide signals (peptide mass fingerprinting, PMF) [288]. Extension of the approach to MS/MS data (tandem mass spectrometric fragment patterns) provides direct access to sequence information [289]. Moreover, due to the unique character of fragment patterns, tandem mass spectrometry allows analysis of complex protein mixtures [290]. However, interpretation of MS/MS spectra in terms of peptide structure remains a challenging task. Typically, it relies on comparison of MS signals and MS/MS fragment patterns with computed data obtained by in silico digestion of defined proteins and subsequent theoretical fragmentation of matched signals, that is typically done by search machines [291]. Unfortunately, in most cases, MS/MS spectra suffer from poor quality, i.e., from incomplete fragment ion series and presence of signals not related to backbone fragmentation, i.e., other than b-, y- or immonium ions [292]. Moreover, insufficient separation prior to MS might result in overlap of several fragmentation patterns and makes straightforward interpretation difficult and possibly ambiguous.

The first successful approach for high-throughput and reliable identification of peptides relied on a partial unique sequence of at least 2–4 residues derived from characteristic mass increments in MS/MS spectra [293]. The next generation of search engines skipped this intermediate step of identification (tag sequence). Thereby, in one group of engines, having the so-called ’SEQUEST’ architecture (PepSearch, SEQUEST, Crux, etc.), matching of theoretical and measured MS/MS spectra relies on cross-correlation analysis [294]. The engines, representing another group (Mascot, OLAV, Andromeda, etc.), estimate the probability of matches between the experimentally measured and calculated fragment *m/z* values occurring by chance [295,296].

In general, the error tolerance of the measured *m/z* and the size of the sequence search space are defined by the resolution of computational searches. The values of parent and fragment ion mass tolerance are defined by mass analyzer type and contribute directly to scoring matches [297]. Since search engines perform multiple comparisons on large datasets with defined mass tolerance, this might lead to some amount of false-positive identifications occurring by chance. Therefore, the actual percentage of such false identifications should be corrected by the false discovery rate (FDR) procedures. For this, tandem mass spectra are searched against a decoy database, which represents a false sequence dataset, typically representing a reversed sequence set [298,299] or database, generated by permutation [300,301,302]. The size of the search sequence space is partially defined by database size. Therefore, the most reliable peptide/protein identification can be achieved when the database size is small (e.g., top-down proteomics). Importantly, the size of the search space denotes not only protein sequences in a primary database but also all considered sequence post-translational modifications (PTMs) [303]. PTMs can be typically recognized by characteristic mass increments, observed in tandem mass spectra [304]. This feature is exploited by the implementation of PTMs in search algorithms [305,306,307]. Thereby, PTMs can be defined as static (i.e., ultimately present at specifically defined amino acid residues, e.g., carbamidomethyl, formed at all cysteine residues after treatment of proteins with iodoacetamide prior to digestion), or variable (optionally occurring at specific residues, e.g., methionine sulfoxide, often formed during sample preparation). Submission of variable modifications results in significantly increased effective search space and time, as the search engine tests all possible combinations of unmodified and modified residues [297,303].

Another popular method of MS/MS-based peptide identification is *de novo* sequencing. It is designed to extract maximal useful information from experimental spectra translating into proper peptide sequences [308,309]. Since the *de novo* sequencing does not require a sequence database, in broad perspective this technique might be the method of choice to identify proteins which are not present in databases, for example, mutation-modified proteins or proteins of poorly or/and unstudied organisms, whose genomes are not sequenced.

**Figure 4:**
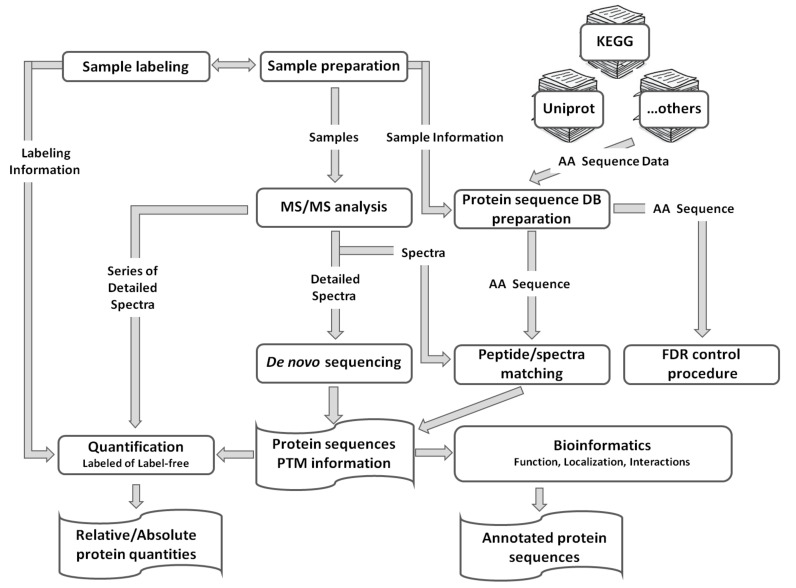
Detailed workflow for LC-based proteomics: protein isolation, sample preparation, and analysis.

Originally, due to large differences in ionization efficiencies of individual peptides, quantitative perspectives of MS-based proteomics seemed limited. Indeed, high variability in charge state, peptide length, amino acid composition, and PTM patterns result in great differences in ion intensities of peptide signals in spectra, even if they represent the same protein. Thus, for accurate quantitation by intensities of quasi-molecular ions, comparisons between different samples can only be done for the same peptide mass-to-charge ratios (*m/z*) acquired under the same conditions in LC-MS experiments [310]. The strategies of quantitative comparison are usually categorized as label-based (most often—stable-isotope-labeling techniques) and label-free approaches [311]. Label-based techniques rely on the fact that stable ^13^C, ^18^O and/or ^15^N-labeled peptides differ from their non-labeled counterparts only in mass but demonstrate the same chromatographic behavior and ionization efficiency [312]. Label-free quantification, in turn, can rely on feature-based quantification, or spectral counting (SC). Thereby, individual signals of peptide ions are treated as features, which are selected during peak peaking and matched during feature map alignment. The SC quantitation methods count the number of MS/MS spectra that are assigned to the same protein, ignoring intensities of corresponding peptide ions [313]. Label-free methods are characterized by a high analytical resolution and dynamic range. Label free methods are supported by numerous software tools, which make a quantitative comparison of proteins across many samples and biological scenarios facile even for a non-expert (Table A3): they include SuperHirn [314], MaxQuant [315], Progenesis [316], OpenMS [317], Proteome Discoverer [318] and the Trans-Proteomic Pipeline [319]. They implement various protein quantification indices based on both spectral counting and ion signal intensities such as emPAI [320], APEX [321], mSCI [322], TOPn [323], and MS stats [324]. For users working with standard workflows and not developing their own algorithms, Progenesis and MaxQuant, which are suitable for fast data analysis, might represent the best solution. If more flexibility and development of task-specific algorithms are required, open-source packages such as Viper [325], mzMine2v [326], or OpenMS [317] represent the best solution. Large proteomics labs and core facilities might appreciate the modularity and automation provided by pipeline tools (Figure 4) [313].

Information on protein identities and their relative or absolute abundance is important, but not sufficient for comprehensive understanding of complex interactions within a biological system. To build a generalized image of multiple simultaneous processes occurring in biological systems, bioinformatic tools are required. These tools allow the assignment of an identified protein to a specific subcellular compartment, molecular function, or biological process as well as establishing its protein-protein interactions (PPI) as a network. The information about protein sub-cellular localization might be found in a database of experimentally derived annotations, available at resource SWISS-PROT [327]. However, even the experimental data collections of well-studied organisms (e.g Arabidopsis or yeast) are not nearly complete [328,329]. To overcome this, in silico prediction approaches (e.g., Plant-mPLoc, BUSCA, LOCALIZER, etc.) relying on specific protein sequence features have been developed [330,331,332,333,334,335]. For example, these algorithms are able to predict the localization of proteins to membranes by the characteristic positions of hydrophobic and hydrophilic residues in their sequence [336]. The information on protein functions can be retrieved from protein sequence databases such as UniProt [337], KEGG [338,339,340]—especially through KEGG-based tools such as BlastKOALA and GhostKOALA—and plant-specific functional database tools such as MapMan [341]. The two first databases organize functional information in Gene Ontology (GO) tags with the following increasing hierarchy order: enzymatic reaction, metabolic pathway, functional process, biological process [342]. GO is also used by PANTHER web server that combines genomes, gene function classifications, pathways, and statistical analysis tools to analyze large-scale genome-wide experimental data [343]. This functional hierarchy scheme, however, might differ from database to database. For example, in MapMan top hierarchical level comprises 34 functional bins [344]. In general, biological function is indirectly associated with the protein sequence, although similar sequences tend to share functionality [345].

Finally, the analysis of PPIs provides essential information for understanding cellular processes. For example, this information can be obtained by structure-based simulation and statistical/machine learning prediction approaches [346,347,348]. Thereby, the first group of methods relies on docking and molecular dynamics methods. This type of calculations requires high computational capacity and rich protein structural information and cannot, therefore, be applied to the size of protein sets usually obtained in proteomics studies. Another group of methods relies on protein molecular descriptors (sequence, domain occurrence, co-expression, etc.) usually retrieved from databases. Information on protein interactions (both experimental and predicted) can be obtained from multiple resources, like STRING (PPIs prediction), BioGRID (PPIs and protein-chemical interactions), Reactome/Plant Reactome (PPIs along with metabolic pathways), etc. (Table A3)

## 7. Future Perspectives

Although seed proteomics represents a well-established field, some of its aspects still need to be elaborated. One of such emerging techniques, only minimally represented in seed research, is peptidomics [349].

Plant peptidomics represents a new rapidly developing field, which became a subject of particular interest after the publication of *A. thaliana* peptidome at the end of the last decade [350]. Plant peptides is a general term for polypeptides of less than 10 kDa which are involved in signaling, regulatory, and defense functions, or produced due to the activity of intracellular proteases [351]. Whereas bioactive peptides, involved in antimicrobial, antifungal, antiviral [352,353], signaling [354,355,356], and plant-microbial interactions [357] are relatively well-addressed in literature, seed peptidome is much less characterized. Seed storage proteins, representing the main seed polypeptide fraction, are considered as powerhouses of bioactive peptides [358,359,360]. Thereby, most often, these peptides comprise the sequences of disordered regions in corresponding proteins [361]. Their liberation can be achieved by treatment of the whole seed material or seed protein isolates with different proteases, specificity of which mostly define the biological activity of resulted mixtures [362]. Different variations of this procedure resulted in the characterization of multiple seed-derived antimicrobial peptides (isolated from the seeds of cycad, guava, barnyard grass, legume *Vicia faba*, and cabbage), recently reviewed by Chai et al. [363]. Analogously, antioxidant dipeptides were isolated from perilla (*Perilla frutescens* L) [364], whereas carrot [365] and chia (*Salvia hispanica* L) seed peptides showed inhibited collagenase, hyaluronidase, tyrosinase, and elastase, demonstrating thereby clear anti-ageing effect [366]. In general, discovery and primary analysis of such peptides relied on the bottom-up proteomics setup. Accordingly, several tools conventionally applied to proteomics data can be used here as well: Mascot database [295], KEGG database, BLAST (Basic Local Alignment Search Tool), etc. [367]. On the other hand, some solutions, developed for untargeted metabolomics and routinely used in non-plant peptidomics can be applied as well: XCMS [368], Agilent Mass Profiler Professional [369] and PepEx, which are useful for site visualization and peptide mapping [370]. Peptide structure predictors based on X-ray crystallography and NMR are also applicable in plant peptidomics—Pepstr [371], PEP-FOLD [372], and PepLook [373]. Plant-specific databases, such as The Plant Proteome Database for *A. thaliana*, the Rice Proteome Database, and Promex can be applied for annotation of bioactive peptides in enzymatic hydrolyzates [374]. More specific tools include the ERA database for cyclic peptide sequences [375] and AMPA searching software for antimicrobial proteins [376].

Redox proteomics represents another rapidly developing field of plant proteome research targeting plant response to oxidative stress—the state when production of reactive molecular species, RMS (mostly reactive oxygen and nitrogen species—ROS and RNS, respectively) is overwhelmed with their detoxification with plant antioxidant systems [377,378,379,380]. The overall goals of redox proteomics are (i) structural characterization of non-enzymatic PTMs, originating from RMS, and (ii) probing the physiological role of the regulatory pathways behind these modifications. To date, ROS-derived modifications are the best characterized PTMs. Thereby, along with multiple other protein residues, cysteine represents the most redox-sensitive residue: it readily forms methionine sulfoxide and cysteinyl sulfenic acid (−SOH), which can be further involved in disulfide formation or oxidation to yield sulfinic (−SO_2_H) and sulfonic acids (−SO_3_H) [381]. Sulfenylation, i.e., formation of sulfenic acid is a reversible reaction underlying so-called redox signaling, which appears to involved hundreds, if not thousands site-specifically sulfenylated proteins [382]. Proteomics approaches to characterize the sulfenylation patterns rapidly developed during the current decade. Whereas the earlier approaches relied on a two-step labeling procedure, which was limited to in vitro experiments [383], implementation of direct labeling techniques, specifically targeting sulphenic acid residues with nucleophilic reagents (e.g., DYn-2) gave access to in vivo studies [384]. A step forward was the implementation of compartment-specific genetically encoded probes—e.g., yeast ACTIVATOR PROTEIN 1; YAP1)-based construct YAP-1C, which contains a redox-active residue C598 amenable of formation of mixed disulfides with in vivo sulfenylated cysteinyl residues [385]. Recently, a highly-reactive diazene (1-(pent-4-yn-1-yl)-1H-benzo[c][1,2]thiazin-4(3H)-one 2,2-dioxide)-based probe was introduced [386] and brought a break-through in analysis of sulfenylated proteome [382].

The above mentioned powerful techniques are still waiting for implementation in seed proteomics, although seed development and germination are well-addressed by non-labeling methods of redox proteomics [387]. Thereby, thiol metabolism is addressed with such gel-based proteomics approaches as diagonal electrophoresis [388]. In this context, the main direction of the further research would be the implementation of redox-labeling techniques in the study of seed maturation and seed germination. Another important aspect would be addressing seed quality and tolerance to prolonged storage. On the one hand, accumulation of antinutritive modifications of reserve proteins can be addressed [11]. On the other, as mitochondria are the marker of seed ageing [389], the state of the art redox proteomics can be employed in the study of the mechanisms behind.

As was mentioned above, characterization of plant sulfenylation patterns (which can be defined as sulfenilome) is still needs to be done. Obviously, for this, implementation of labeling techniques combined with LC-MS-based techniques in plant redox proteomics is strongly mandatory. Without any doubt, this will give access to regulatory mechanisms, based on redox signaling.

It is important to note that those structural and functional manifestations of oxidative stress in plant proteome are much more diverse than it usually thought. The corresponding changes in plant protein structure and function need to be considered in the context of the physiological and metabolic response of the plant to oxidative stress. For example, it is well-known that seed maturation is accompanied by accumulation of reserve molecules [390], to a great extent represented by carbohydrates and lipids [391]. Accumulation of fatty acids and carbohydrates at the early steps of seed maturation and their mobilization during germination may affect the patterns on non-enzymatic modifications of seed proteins, which are a highly abundant component of, for example, legume seeds. Indeed in seeds, oxidative stress, accompanying both their maturation and ageing [392,393,394,395], develops at the background of high contents of reducing sugars (generated by de-polymerization of reserve polysaccharides) and unsaturated fatty acids (mostly formed during mobilization of reserve lipids) [396,397,398,399,400]. These conditions might result in oxidative degradation of carbohydrates and lipids/fatty acids via metal-catalyzed monosaccharide autoxidation and lipid peroxidation, respectively, yielding in both cases reactive carbonyl compounds (RCCs) [401,402]. These intermediates are readily involved in glyco- and lipoxidation of proteins resulting in the formation of advanced glycoxidation and lipoxidation end products (AGEs and ALEs, respectively), which are potentially pro-inflammatory in mammals and impact on ageing and metabolic diseases [403,404]. Hence, this phenomenon needs to be taken into account when seeds are stored for long periods of time [405], or when the processes, accompanying storage, are simulated in models of accelerated seed ageing [406]. As this human physiology aspect in plant science remains mostly unstudied, these aspects need to be addressed in much more detail in next future. For this, a coordinated effect of plant biochemists, nutritional scientists and human physiologists is required.

Although glyco- and lipoxidation in plants has been addressed by biochemical methods [399,407], a comprehensive analysis of non-enzymatic PTMs in ageing seeds still needs to be done. Most efficiently, such a profiling of glyco- and lipoxidative modifications might rely on LC-MS-based approach with a special focus on in-depth analysis of low-abundant species using enrichment/depletion, pre-fractionation, and result-based exclusion of non-modified peptides from fragmentation [171,177]. Alternatively, modified peptides can be selectively detected by highly sensitive and specific precursor ion scanning and further identified by targeted MS/MS experiments [408,409]. Such experiments might rely on MS/MS analyses with model oxidized, glycated, as well as glyco- and lipoxidized peptides [408,409,410,411,412,413], which can be obtained by solid-phase peptide synthesis (SPPS) with high yield and purity [414,415,416]. Thereby, formation and degradation of early and advanced glycation products can be dissected by glycation models based on synthetic peptides. On the one hand, such an approach may give access to the pathways of AGE formation [106,171,417]. On the other hand, it can deliver information about carbonyl compounds involved in glycation [105]. Based on this approach, highly sensitive and reliable LC-MS/MS-based quantification techniques can be developed [418,419], although their application to seed research has still not been accomplished. An alternative approach relies on exhaustive enzymatic hydrolysis followed with MS/MS analysis of resulted amino acid adducts by QqQ-MS using the stable isotope dilution [420] and standard addition [421] approach. This strategy was successfully applied to the assessment of glycation and oxidation in plants [422]. Recently, we optimized this methodology for the analysis of seed protein adducts [93,423]. However, it is just a beginning of a big work, which is still to be done.

Although glycation and lipoxidation are well-known markers of ageing in mammals [424], this was only recently confirmed for plants. Indeed, AGEs were shown to impact ageing not only in plant leaves [425] but also in such specialized structures, as legume root nodules [212], where multiple regulatory proteins and transcription factors were involved in age-dependent modifications. It raises the question about a possible regulatory role of this type of modification in plants. Keeping in mind a pronounced pro-inflammatory effect of glyco- and lipoxidized proteins on organisms of heterotrophic mammalian consumers of seed-derived food, one can conclude that this aspect is highly relevant for food chemistry as well [403]. Taking all this into account, patterns of AGEs, and ALEs in seed proteins of crop plants need to be comprehensively characterized.

## 8. Conclusions

The seed proteome represents a highly-complex system, only a part of which has been sufficiently described to date. The patterns of PTMs in seed polypeptides are also just partly characterized. Indeed, only phosphorylation and glycosylation were comprehensively addressed so far, whereas the sites of other enzymatic and non-enzymatic modifications remain mostly unknown. Obviously, the depth of its characterization needs to be increased for a better understanding of the processes accompanying seed development and germination. For this, simultaneous consideration of the datasets acquired by LC-MS and 2D-GE is necessary. This would not only increase the proteome coverage but also might allow cross-validation of the obtained data and increase the overall result reliability. On the other hand, due to their potential involvement in cellular regulatory networks, non-enzymatic modifications (first of all, oxidation and glycation) and mechanisms, underlying in vivo physiological effects, need to be addressed. To some extent, the depth of proteome analysis can be increased by the implementation of additional enrichment/depletion or pre-fractionation steps in established workflows. However, for further increase the analytical power of seed proteomics, its methodology needs to be complemented with approaches and techniques currently employed in other fields of proteomics research, like, medical, ecological, food proteomics. We hope that our contribution will help seed biologists to increase the methodological power of seed proteomics and to make a step forward to understanding the fundamental processes of seed development and germination.

## Abbreviations

2D-GE	Two-dimensional gel electrophoresis
AALS I/II	Anionic acid labile surfactant I/II
ABA	Abscisic acid
AGEs	Advanced glycoxidation end products
ALEs	Advanced lipoxidation end products
BAC	Boronic acid chromatography
CALS I/II	Cationic acid labile surfactant I/II
CHAPS	3-(3-cholamidopropyl)dimethylammonio)-1-propanesulfonate
CMC	Critical micelle concentration
DCM	Dichloromethane
DDA	Data-dependent acquisition
DIA	Data-independent acquisition
DIGE	Difference gel electrophoresis
DTT	Dithiothreitol
ESI	Electrospray ionization
FASP	Filter-aided sample preparation
FDR	False discovery rate
GASP	Gel-aided sample preparation
GPF	Gas-phase fractionation
HILIC	Hydrophilic interaction liquid chromatography
HiT-Gel	High-throughput in-gel digestion technique
IEF	Isoelectrofocusing
IPG	Immobilized pH gradient
KDS	Potassium dodecyl sulfate
LC	Liquid chromatography
LIT	Linear ion trap
LODs	Limits of detection
MALDI	Matrix-assisted laser desorption-ionization
MRM	Multiple reaction monitoring
MS	Mass spectrometry
MS/MS	Tandem mass spectrometry
PMF	Peptide mass fingerprinting
PPI	Protein-protein interactions
PQPs	Peptide query parameters
PRM	Parallel reaction monitoring
PTMs	Post-translational modifications
PVP	Polyvinylpyrollidone
RCCs	Reactive carbonyl compounds
RPC	Reversed phase chromatography
SDC	Sodium deoxycholate
SDS	Sodium dodecyl sulfate
SDS-PAGE	Polyacrylamide gel electrophoresis in sodium dodecyl sulfate
SPE	Solid phase extraction
SPPS	Solid phase peptide synthesis
SRM	Selected reaction monitoring
TCA	Trichloroacetic acid
TOF	Time of flight
UHPLC	Ultra-high performance liquid chromatography
XIC	Extracted ion chromatogram
ZALSI/II	Zwitterionic acid labile surfactant I/II

## Appendix A

**Table A1:** Overview of protein extraction techniques.

#	Extraction Technique	Extraction Buffer	Chaotropic Agents	Detergents	Reducing and Chelating Additives	Further Additives	Precipitation (Vprecipitant: Vextract)	Isolate Cleaning	Reconstitution	Ref
1	Phenol extraction	0.5 mol/L Tris-HCl (pH 7.5)	none	none	2% (*v*/*v*) ME, 50 mmol/L EDTA	1–15% (*w*/*v*) PVPP, PIC	0.1 mol/L AmAc/MeOH (5:1)	MeOH (3×), acetone (3×)	SDS-PAGE SB, IEF buffer, SB for LC-MS	[89,177]
2	TCA/acetone extraction	10% (*w*/*v*) TCA in acetone	none	none	2% (*v*/*v*) ME	1–15% (*w*/*v*) PVPP, PIC	precipitation at the extraction step	acetone (3×)	SDS-PAGE SB, IEF buffer	[89,94,426]
3	Extraction with urea/thiourea buffer	14 mmol/L Tris-HCl	7 mmol/L urea, 2 mmol/L thiourea	2% (*v*/*v*) Triton X-100, 58 mmol/L CHAPS	none	PIC, 18 mmol/L ampholytes	none	none	solubilization at the extraction step	[82]
4	Acetone precipitation	20 mmol/L Tris-HCl (pH 7.5)	none	1% (*v*/*v*) Triton X-100	10 mmol/L EGTA, 1 mmol/L DTT	1 mmol/L PMSF, 250 mmol/L sucrose	precipitation at the extraction step	acetone (3×)	SDS-PAGE SB	[108]
5	Extraction with SDS-Tris buffer	125 mmol/L Tris-HCl	none	4% (*w*/*v*) SDS	2% (*v*/*v*) ME	20% (*v*/*v*) glycerol	none	none	solubilization at the extraction step	[110]
6	Extraction HEPES buffer/delipidation (DCM)	50 mmol/L HEPES buffer	none	none	1 mmol/L EDTA	1 mmol/L PMSF, 0.1 mmol/L nDHGA	acetone (1:5)	none	SDS-PAGE SB, IEF buffer	[113]
7	Extraction with urea/thiourea buffer	6 mmol/L Tris-HCl,4.2 mmol/L Trizma R	7 mmol/L urea, 2 mmol/L thiourea	4% (*w*/*v*) CHAPS	3% (*w*/*v*) DTT	PIC, DNAse I, RNAse A	none	none	solubilization at the extraction step	[427]
8	MeOH/CHCl3 precipitation, delipidation (PE)	50 mmol/L Tris-HCl (pH 8.8)	none	1% (*w*/*v*) SDS	0.07% (*v*/*v*) ME, 1.5 mmol/L KCl	PIC, delipidation (PE)	MeOH/CHCl_3_/ddH_2_O (4:1:3)	SPE	8 mol/L urea in 50 mmol/L ABC	[95]
9	TCA/acetone precipitation, delipidation (PE)	50 mmol/L Tris-HCl (pH 8.8)	none	1% (*w*/*v*) SDS	0.07% (*v*/*v*) ME, 1.5 mmol/L KCl	PIC, delipidation (PE)	acetone (1:4)	SPE	8 mol/L urea in 50 mmol/L ABC	[95]
10	Acetone precipitation, delipidation (PE)	50 mmol/L Tris-HCl (pH 8.8)	none	1% (*w*/*v*) SDS	0.07% (*v*/*v*) ME, 1.5 mmol/L KCl	PIC, delipidation (PE)	acetone/10% (*w*/*v*) TCA (1:4)	SPE	8 mol/L urea in 50 mmol/L ABC	[95]
11	Urea solubilization buffer	8 mol/L urea, 2% (*w*/*v*) ampholyte (pH 3–10)	8 mol/L urea	4% (*w*/*v*) CHAPS	none	none	none	2D cleanup kit (GE Healthcare)	solubilization at the extraction step	[96]
12	Thiourea/urea solubilization buffer delipidation (hexane)	5 mol/L urea, 2 mol/L thiourea, 0.8% (*w*/*v*) ampholytes (pH 3–10)	5 mol/L urea, 2 mol/L thiourea	4% (*w*/*v*) CHAPS	65 mmol/L DTT	delipidation (hexane)	none	none	solubilization at the extraction step	[96]
13	Phenol extraction	0.1 mol/L Tris–HCl (pH 8.8)	none	none	10 mmol/L EDTA, 0.4% (*v*/*v*) ME	none	AmAc/MeOH (5:1)	0.1mol/L AmAc/MeOH (2×) acetone (2×) MeOH (1×)	8 mol/L urea, 2 mol/L thiourea, 2% (*w*/*v*) CHAPS, 2% (*v*/*v*) Triton X-100, 50 mmol/L DTT, 0.5% (*w*/*v*) ampholytes (pH 3–10)	[96]
14	Modified TCA/acetone precipitation/Urea solubilization extraction	10% (*w*/*v*) TCA in acetone	none	none	0.07% (*v*/*v*) ME	none	precipitation at the extraction step	acetone (2–3×)	9 mol/L urea, 1% (*w*/*v*) CHAPS, 1% (*w*/*v*) ampholytes pH (3–10), 1% (*w*/*v*) DTT	[96]
15	Phenol extraction	0.5 mol/L Tris-HCl, (pH 7.5)	none	none	2% (*v*/*v*) ME, 50 mmol/L EDTA	10% (*w*/*v*) PVPP, 1 mmol/L PMSF	0.1 mol/L AmAc/MeOH (2x)	none	IEF buffer, SDS-PAGE SB	[426]
16	Tris/TCA extraction	100 mmol/L Tris, TCA in acetone (pH 8.5)	none	none	5 mmol/L DTT, 1 mmol/L EDTA, 0.07% (*v*/*v*) ME	1 mmol/L PMSF	Precipitation at the extraction step	0.07% (*v*/*v*) ME in acetone	IEF buffer, SDS-PAGE SB	[426]
17	Tris-base extraction	40 mmol/L Tris	5 mol/L urea, 2 mol/L thiourea	2% (*w*/*v*) CHAPS	2% (*v*/*v*) ME	5% (*w*/*v*) PVP	Precipitation at the extraction step	0.07% (*v*/*v*) ME in acetone	IEF buffer, SDS-PAGE SB	[426]
18	TCA/acetone extraction	10% (*v*/*v*) TCA in acetone	none	none	20 mmol/L DTT	none	Precipitation at the extraction step	Acetone or 20 mmol/L DTT in acetone, or 10% ddH2O in acetone or 20 mmol/L DTT, 10% ddH2O in acetone	IEF SB	[100]
19	Phenol extraction	50 mmol/L Tris-HCl (pH 7.5)	none	none	5 mmol/L EDTA, 5 mmol/L DTT	1% (*w*/*v*) PVPP, 1 mmol/L PMSF	Acetone: supernatant (5:1)	none	Urea buffer (50 mmol/L HEPES (pH 7.8), 8 mol/L urea) SB for LC-MS	[103]
20	TCA/acetone extraction	10% (*v*/*v*) TCA in acetone	none	none	10 mmol/L DTT	10% (*w*/*v*) PVPP, 55 mmol/L iodoacetamide, 0.5 mol/L TEAB	Precipitation at the extraction step	Acetone (3×)	TEAB buffer, IEF SB, SB for LC-MS	[102]
21	TCA/acetone and methanol washes and phenol extraction	Phenol (pH 8.0): SDS (1:1)	none	none	none	none	0.1 mol/L AmAc/MeOH	10% (*v*/*v*) TCA/acetone 0.1 MAmAc 80% MeOH 80% acetone	SDS-PAGE SB, IEF buffer	[111]
22	Tris–HC l/ TCA/acetone extraction	0.1 mol/L Tris–HCl (pH 6.8), 10% (*v*/*v*) TCA/acetone	none	1% (*w*/*v*) SDS	0.1 mol/L DTT	none	10% (*v*/*v*) TCA/acetone	10% (*v*/*v*) TCA/acetone aqueous 10% TCA (2x) dH2O (1x) acetone (1x)	SDS-PAGE SB	[111]

ABC, ammonium bicarbonate buffer; AmAc, ammonium acetate; CHAPS, 3-[(3-cholamidopropyl)dimethylammonio]-1-propanesulfonate hydrate; DCM, dichloromethane; DTT, dithiothreitol; EDTA, ethylenediaminetetraacetic acid; EGTA, ethylene-bis(oxyethylenenitrilo)tetraacetic acid; HEPES, 4-(2-hydroxyethyl)piperazine-1-ethanesulfonic acid; IEF, isoelectric focusing; LC-MS, liquid chromatography-mass spectrometry; ME, ß-mercaptoethanol; nDHGA, nordihydroguaiaretic acid; PE, petroleum ether; PIC, protein inhibitor cocktail; PMSF, phenylmethylsulfonyl fluoride; PVPP, polyvinylpolypyrrolidone; SB, sample buffer; SDS, sodium dodecyl sulfate; SDS-PAGE, sodium dodecyl sulfate polyacrylamide gel electrophoresis; SPE, solid phase extraction; TCA, trichloroacetic acid; TEAB, triethylammonium bicarbonate; Tris, tris(hydroxymethyl)aminomethane.

**Table A2:** Digestion strategies used in bottom-up proteomics.

#	Object/Tissue	Methodology						
		Protein Isolation	Detergent/Chaotropic Agent	Reduction/Alkylation	Protease	Chromatographic System	MS	Ref
**Plant Objects**								
1	*Brassica napus* L., seeds	detergent extraction, phenol extraction	none (in-gel digest)	DTT/IA	trypsin	none	MALDI-TOF/TOF-MS	[428]
2	*Lupinus luteus* L, Seeds	delipidation with hexane, acetone precipitation	none (in-gel digest)	DTT/IA	trypsin	RP C18, L-column water-ACN grad., 0.1% (*v*/*v*) FA	ESI-IT-MS	[138]
3	*Cicer arietinum* L., plasma membrane aerial parts	chloroform/methanol (5:4) extraction	none (in-gel digest)	DTT/IA	trypsin	RP C18, water-ACN grad., 0.1% (*v*/*v*) FA	ESI-LTQ-Orbitrap-MS	[31]
			4% (*w*/*v*) SDS/none	DTT/IA	trypsin			
4	*Glycine max* L., seeds	delipidation with hexane, extraction with SDS-PAGE SB	none (in-gel digest)	DTT/IA	trypsin	none	MALDI-TOF/TOF-MS	[139]
				DTT/none		RP, BEH130C18, water-ACN grad. 0.1% (*v*/*v*) FA	ESI-QqTOF-MS	
5	*Glycine max* L.,seeds	2 steps extraction: protamine sulfate precipitationTCA/acetone precipitation	none (in-gel digest)	DTT/IA	trypsin	none	MALDI-TOF/TOF-MS	[140,186]
			4% (*w*/*v*) SDS/ 8 mol/L urea	DTT/IA	trypsin	RP, C18. water-ACN grad. 0.1% (*v*/*v*) FA	ESI-Q-Orbitrap-MS	
6	*Arabidopsis thaliana*, aerial parts	1) detergent extraction 2) aq. buffer extraction	none (in-gel digest)	DTT/IA	trypsin	RP, C18 PepMap water-ACN grad. 0.1% (*v*/*v*) FA	ESI-Q-Orbitrap-MS	[141]
7	*A. thaliana*, leaves	phenol extraction	0.5% (*w*/*v*) AALS/ 7 mol/L urea	TCEP/IA	trypsin	RP, water-ACN grad. 0.1% (*v*/*v*) FA	ESI-LIT-Orbitrap ESI-QqTOF-MS	[169]
8	*G. max*, seeds	1) detergent extractionacetone precipitation	none (in-gel digest)	none	trypsin	RP, C18 PepMap column water-ACN grad. 0.1% (*v*/*v*) FA	ESI-LIT-MS	[108]
9	*Chenopodium quinoa* W., seeds	1) detergent extraction 2) methanol/chloroform or acetone precipitation	none/8 mol/L urea	DTT/IA	trypsin	RP, Acclaim-C18 column water-ACN grad. 0.1% (*v*/*v*) FA	ESI-LIT-Orbitrap-MS	[95]
10	*Solanum esculentum* L. roots,	detergent extraction for isolation of cell microsomal fractions by centrifugation	1) methanol ** 2) 0.2% PPS SilentSurfactant/none 3) 0.2% RGS/none 4) none/6 mol/L GdnCl	TCEP/IA	trypsin	RP, BEH C18 water-ACN grad. 0.1% (*v*/*v*) FA	ESI-QqTOF-MS	[29]
11	*Vitisriparia*, leaves	1) detergent extraction 2) methanol-chloroform extraction	none (in-gel digest)	DTT/IA	trypsin	RP, Magic C18AQ resin water-ACN grad. 0.1% (*v*/*v*) FA	ESI-LIT-Orbitrap-MS	[187]
			50% TFE **	DTT/IA	Lys-C, trypsin			
12	*Cucumis sativus* L., seeds	TCA/acetone (1:9 *w*/*v*) precipitation	none/8 mol/L urea	DTT/IA	trypsin	RP, C18. water-ACN grad. 0.1% (*v*/*v*) FA	ESI-Q-Orbitrap-MS	[188]
13	*Hordeum vulgare* L., leaves	detergent extraction	1) none/8 mol/L urea 2) 2% (*w*/*v*) SDS/8 mol/L urea 3) 1% SDC/none 4) 2% SDC/none	DTT/IA	trypsin	RP, Reprosilpur 120 C18 water-ACN grad. 0.1% (*v*/*v*) FA	ESI-Q-Orbitrap-MS	[194]
14	*Zea mays* L., seeds	1) detergent extraction 2) TCA/acetone (1:9*w*/*v*) extraction	none (in-geldigest)	DTT/IA	trypsin	RP, Eksigent C8-CL-120 column water-ACN grad. 0.1% (*v*/*v*) FA	ESI-QqQ-MS	[429]
15	*Solanum tuberosum* L. leaves,	2 steps: 1) detergent extraction 2) co-immunoprecipitation	0.1% ProteaseMAX™ surfactant/none	TCEP/MMTS	trypsin	RP, Reprosil C18-AQ, water-ACN grad. 0.1% (*v*/*v*) FA	ESI-Q-LIT-Orbitrap-MS	[430]
16	*H. vulgare* caryopses,	10% TCA, 0.07% (*w*/*v*) β-mercaptoethanol in acetone	0.1% RGS/none	DTT/IA	trypsin	RP, C18 water-ACN grad. 0.1% (*v*/*v*) FA	ESI-QqTOF-MS	[203]
17	*B. napus*, seedling	1) aq. buffer extraction 2) phenol extraction	0.02% (*w*/*v*) AALS/8 mol/L urea, 2 mol/L thiourea	TCEP/IA	trypsin	RP, Acclaim PepMap 100 C18 column water-ACN grad. 0.1% (*v*/*v*) FA	ESI-Q-LIT—Orbitrap-MS	[26]
**Non-plant objects**								
18	Myoglobin, bacteriorhodopsin, BSA *	none	1) 0.1–1.0% RGS/none 2) 1.0% SDC/none 3) 0.1–1.0% SL/none	none	trypsin	none	MALDI-TOF/TOF-MS	[196]
	Rat, liver *	isolation of cell membranes by centrifugation in the gradient of sucrose	1) 1.0% SDS/none 2) 1.0% SL/none 3) 1.0% RGS/none 4) 1.0% SDC/none	DTT/IA	trypsin	RP, C18 PepMap column water-ACN grad. 0.1% (*v*/*v*) FA	ESI-IT-MS	
19	Rat, liver *	membrane isolation, centrifugation in sucrose gradient	1) 1%(*w*/*v*) SDS/none 2) 1%(*w*/*v*)RGS/none 3) none/8mol/L urea 4) 60% (*v*/*v*) methanol **	DTT/IA	trypsin	RP, C18PepMap column water-ACN grad. 0.1% (*v*/*v*) FA	ESI-IT-MS	[168]
20	BSA, ubiquitin, myoglobin, PC3 cells *	cell lysis, isolation of cell membranes by centrifugation	none	DTT/IA	trypsin	RP, C18. water-ACN grad. 0.1% (*v*/*v*) FA	ESI-QqTOF-MS	[189]
21	*Rhodopseudomona spalustris **	acid extraction and sucrose density fractionation	0, 60 or 80% acetonitrile ** in 50 mmol Tris-HCl, 10 mmol/L CaCl2	DTT/none	trypsin	RP, Vydac C18 water-ACN grad. 0.1% (*v*/*v*) FA	ESI-IT-MS ESI-FT-ICR-MS	[192]
	Mixture of protein standards *	none	none/6 mol/LGdnHCl					

* not established in seeds or generally in plants, shown as a method to be potentially employed in seed proteomics, ** non detergent or chaotropic reagent used for solubilization; AALS, anionic acid labile surfactant; ACN, acetonitrile; BSA, bovine serum albumin; DTT, dithiothreitol; ESI, electrospray ionization; FA, formic acid; FT-ICR, Fourier transform-ion cyclotron resonance; GdnHCl, guanidinium chloride; IA, iodoacetamide; IT, ion trap; LIT—linear ion trap; MALDI-TOF, matrix assisted laser desorption/ionization—time of flight; MMTS, methyl methanethiosulfonate; MS, mass spectrometry; nano-scaled liquid chromatography; Q, quadrupole mass analyzer; QqTOF, quadrupole-time of flight; RGS, RapiGest SF; RP, reversed phase; SDC, sodium deoxycholate; SDS, sodium dodecyl sulfate; SL, sodium laurate; TCA, trichloroacetic acid; TCEP, *tris*-(2-carboxyethyl)-phosphine; TFE, trifluoroethanol.

**Table A3:** Software tools for processing and post-processing for proteomics data.

#	Tool	Version	Supported Platform	GUI/CMD	Open Source	Input Formats	Quantification Technique	Ref
1	MaxQuant	v1.6.3.3	Windows, Linux	+/+	+	AB SCIEX (*.wiff), mzXML, Thermo (*.raw), Agilent & Bruker Daltinics (*.d), Uimf (*.uimf)	LFQ/label-based	[431]
2	Peaks	v2.0	Windows, Linux	+/+	−	CID/CAD/HCD/ETD/ECD/EThCD	LFQ/label-based	[432]
3	OpenMS/TOPP	v2.0	Windows, Linux, Mac OS	+/+	+	mzML, mzXML, mzData	LFQ/label-based	[433]
4	Progenesis QI	v2.3	Windows			Agilent & Bruker Daltinics (*.d), AB SCIEX (*.wiff), mzML, mzXML, Thermo& Agilent (*.raw)	LFQ/label-based	[434]
5	Proteome discoverer	v2.2	Windows	+/−	−	mzXML, mzDATA, mzML, MSF	LFQ/label-based	[435]
6	Census	v2.3	Windows, Linux, Mac OS	+/−	−	mzXML	LFQ/label-based possibility to self-define mass tags	[436]
7	SILVER	V3.0	Windows	+/−	+	mzXML, *.raw	SILAC	[437]
8	msInspect	v3.1	Windows, Linux, Mac OS	+/+	+	mzXML	LFQ/label-based	[313,438]
9	mzMine2	v2.6	Windows, Linux, Mac OS	+/−	+	mzML, mzXML, mzData, NetCDF, RAW (Thermo)	LFQ	[326]
10	MassChroQ	v2.2.12	Windows, Linux	−/+	+	mzXML, mzML	LFQ/label-based	[439]
11	Skyline	v4.1	Windows, Linux	+/+	+	.sky, .skyd, mzML, mzXML, major vendor formats	LFQ	[440]
12	DIA-Umpire	v2.0	Windows, Linux	−/+	+	mzXML	ICAT, ^18^O	[441]
13	Viper	v3.49	Windows, Linux	+/−	+	PEK, .CSV (Decon2LS), .mzXML,.mzData	ICAT, ^18^O	[325]
14	OpenSWATH	v2.2	Windows, Linux, Mac OS	−/+	+	mzML, mzXML, TraML	LFQ/label-based	[215]
15	TPP	v5.1.0	Windows, Linux, Mac OS	+/+	+	mzXML, .RAW (Thermo), wiff, baf (Brucker), pepXML	LFQ	[319,442]
16	moFF	v2.0	Windows, Linux, Mac OS	+/+	+	Thermo (.raw), mzML	LFQ	[443]
17	Mascot Distiller	v2.7	Windows	+/+	−	mzML, mzXML, mzData, major vendors	LFQ/label-based	[444]
18	Corra	v3.1	Linux			mzML, pepXML	LFQ/label-based	[313,445]
19	FlashLFQ	v0.1.61	Windows	−/+	+	MzML, raw	LFQ	[446]
20	Thermo Scientific ProSightPC/ProSightPD	v4.0/v2.0	Windows	+/−	−	Thermo (*.raw), .PUF, UniProt XML, FASTA, UniProKB	LFQ/abel-based	[447]
21	MassHunter	v10.0	Windows	+/−	−	Agilent (d)	LFQ/label-based	[448]
22	Mercator4.0	v2.0	Web tool, Windows, Linux	+/−	+	FASTA	On-line functional annotation tool sequences	[449]
23	BlastKOALA	-	Web tool	−/−	−	FASTA	Automatic annotation server for genome and metagenome sequences	[341]
24	WoLF PSORT	-	Web tool	−/−	−	FASTA	Prediction of sub-cellular localization	[331]
25	BUSCA (Bologna Unified Subcellular Component Annotator)	-	Web tool	−/−	−	FASTA	Prediction of sub-cellular localization	[331,333]
26	eggNOG-mapper	v2	Web tool	−/−	+	FASTA	Functional annotation of large sets of sequences *	[450]
27	PANTHER	v.14.0	Web server	−/−	−	FASTA, gene ID(.txt)	Large-scale genome-wide experimental data **	[343]
28	STRING	v11.0	Web server	−/−	−	protein name (.txt), gene ID (.txt)	Protein-protein association networks	[451]

* based on fast orthology assignments using pre-computed eggNOG v5.0 clusters and phylogenies, ** system that combines genomes, gene function classifications, pathways and statistical analysis tools;^18^O, ^18^O labeling by ^18^O-enriched water; CMD, command line; GUI, graphical user interface; ICAT, isotope-coded affinity tag; LFQ, label free quantification; SILAC, stable isotope labeling by/with amino acids in cell culture.

## References

[1] FAO (2018). Seeds Toolkit—Module 5: Seed Marketing.

[2] FAO (2013). Statistical Book. Part 3. Feeding the World.

[3] FAO, IFAD, UNICEF, WFP, WHO (2018). The State of Food Security and Nutrition in the World 2019. Building Climate Resilience for Food Security and Nutrition.

[4] Bradford K.J., Dahal P., Van Asbrouck J., Kunusoth K., Bello P., Thompson J., Wu F. (2018). The dry chain: Reducing postharvest losses and improving food safety in humid climates. Trends Food Sci. Technol..

[5] Miller J.K., Herman E.M., Jahn M., Bradford K.J. (2010). Strategic research, education and policy goals for seed science and crop improvement. Plant Sci..

[6] Bewley J.D., Bradford K.J., Hilhorst H.W.M., Nonogaki H. (2013). Seeds: Physiology of Development, Germination and Dormancy.

[7] Leprince O., Pellizzaro A., Berriri S., Buitink J. (2017). Late seed maturation: Drying without dying. J. Exp. Bot..

[8] Finch-Savage W.E., Bassel G.W. (2016). Seed vigour and crop establishment: Extending performance beyond adaptation. J. Exp. Bot..

[9] Marques A., Buijs G., Ligterink W., Hilhorst H. (2018). Evolutionary ecophysiology of seed desiccation sensitivity. Funct. Plant Biol..

[10] Szarka A., Tomasskovics B., Bánhegyi G. (2012). The Ascorbate-glutathione-*α*-tocopherol. Triad in Abiotic Stress Response. Int. J. Mol. Sci..

[11] Frolov A., Mamontova T., Ihling C., Lukasheva E., Bankin M., Chantseva V., Vikhnina M., Soboleva A., Shumilina J., Mavropolo-Stolyarenko G. (2018). Mining seed proteome: From protein dynamics to modification profiles. Biol. Commun..

[12] Aguirre M., Kiegle E., Leo G., Ezquer I. (2018). Carbohydrate reserves and seed development: An overview. Plant Reprod..

[13] Baud S. (2018). Seeds as oil factories. Plant Reprod..

[14] Gallardo K., Thompson R., Burstin J. (2008). Reserve accumulation in legume seeds. Comptes Rendus Biol..

[15] Wang W.-Q.Q., Liu S.-J.J., Song S.-Q.Q., Møller I.M. (2015). Proteomics of seed development, desiccation tolerance, germination and vigor. Plant Physiol. Biochem..

[16] Miernyk J.A., Hajduch M. (2011). Seed proteomics. J. Proteomics.

[17] Copeland L.O., McDonald M.B. (1999). The chemistry of seeds. Princ. Seed Sci. Technol..

[18] Robić G., Farinas C.S., Rech E.L., Miranda E.A. (2010). Transgenic soybean seed as protein expression system: Aqueous extraction of recombinant *β*-glucuronidase. Appl. Biochem. Biotechnol..

[19] Schmidt M.A., Herman E.M. (2008). Proteome rebalancing in soybean seeds can be exploited to enhance foreign protein accumulation. Plant Biotechnol. J..

[20] Rathi D., Gayen D., Gayali S., Chakraborty S., Chakraborty N. (2016). Legume proteomics: Progress, prospects, and challenges. Proteomics.

[21] Shewry P.R., Casey R., Shewry P.R., Casey R. (1999). Seed Proteins. Seed Proteins.

[22] Gallardo K., Job C., Groot S.P.C., Puype M., Demol H., Vandekerckhove J., Job D. (2001). Proteomic analysis of arabidopsis seed germination and priming. Plant Physiol..

[23] Gallardo K., Le Signor C., Vandekerckhove J., Thompson R.D., Burstin J. (2003). Proteomics of medicago truncatula seed development establishes the time frame of diverse metabolic processes related to reserve accumulation. Plant Physiol..

[24] Catusse J., Job C., Job D. (2008). Transcriptome- and proteome-wide analyses of seed germination. Comptes Rendus Biol..

[25] Rajjou L., Lovigny Y., Groot S.P.C., Belghazi M., Job C., Job D. (2008). Proteome-wide characterization of seed aging in Arabidopsis: A comparison between artificial and natural aging protocols. Plant Physiol..

[26] Frolov A., Didio A., Ihling C., Chantzeva V., Grishina T., Hoehenwarter W., Sinz A., Smolikova G., Bilova T., Medvedev S. (2018). The effect of simulated microgravity on the *Brassica napus* seedling proteome. Funct. Plant Biol..

[27] Vanderschuren H., Lentz E., Zainuddin I., Gruissem W. (2013). Proteomics of model and crop plant species: Status, current limitations and strategic advances for crop improvement. J. Proteomics.

[28] Miernyk J.A. (2014). Seed proteomics. Plant Proteomics. Methods in Molecular Biology (Methods and Protocols).

[29] Mbeunkui F., Goshe M.B. (2011). Investigation of solubilization and digestion methods for microsomal membrane proteome analysis using data-independent LC-MSE. Proteomics.

[30] Balmer Y., Vensel W.H., DuPont F.M., Buchanan B.B., Hurkman W.J. (2006). Proteome of amyloplasts isolated from developing wheat endosperm presents evidence of broad metabolic capability. J. Exp. Bot..

[31] Barua P., Subba P., Lande N.V., Mangalaparthi K.K., Prasad T.S.K., Chakraborty S., Chakraborty N. (2016). Gel-based and gel-free search for plasma membrane proteins in chickpea (*Cicer arietinum* L.) augments the comprehensive data sets of membrane protein repertoire. J. Proteomics.

[32] Yadeta K.A., Elmore J.M., Coaker G. (2013). Advancements in the analysis of the *Arabidopsis* plasma membrane proteome. Front Plant Sci.

[33] Komatsu S., Wang X., Yin X., Nanjo Y., Ohyanagi H., Sakata K. (2017). Integration of gel-based and gel-free proteomic data for functional analysis of proteins through Soybean Proteome Database. J. Proteomics.

[34] Bourgeois M., Jacquin F., Savois V., Sommerer N., Labas V., Henry C., Burstin J. (2009). Dissecting the proteome of pea mature seeds reveals the phenotypic plasticity of seed protein composition. Proteomics.

[35] Komatsu S., Hashiguchi A. (2018). Subcellular proteomics: Application to elucidation of flooding-response mechanisms in soybean. Proteomes.

[36] Wang W.Q., Møller I.M., Song S.Q. (2012). Proteomic analysis of embryonic axis of *Pisum sativum* seeds during germination and identification of proteins associated with loss of desiccation tolerance. J. Proteomics.

[37] Hajduch M., Casteel J.E., Hurrelmeyer K.E., Song Z., Agrawal G.K., Thelen J.J. (2006). Proteomic analysis of seed filling in *Brassica napus* developmental characterization of metabolic isozymes using high-resolution. Plant Physiol..

[38] Zhang H., Wang W.Q., Liu S.J., Møller I.M., Song S.Q. (2015). Proteome analysis of poplar seed vigor. PLoS ONE.

[39] Ventura L., Donà M., Macovei A., Carbonera D., Buttafava A., Mondoni A., Rossi G., Balestrazzi A. (2012). Understanding the molecular pathways associated with seed vigor. Plant Physiol. Biochem..

[40] Catusse J., Meinhard J., Job C., Strub J.M., Fischer U., Pestsova E., Westhoff P., Van Dorsselaer A., Job D. (2011). Proteomics reveals potential biomarkers of seed vigor in sugarbeet. Proteomics.

[41] Rajjou L., Duval M., Gallardo K., Catusse J., Bally J., Job C., Job D. (2012). Seed germination and vigor. Annu. Rev. Plant Biol..

[42] Hatzig S.V., Frisch M., Breuer F., Nesi N., Ducournau S., Wagner M.-H., Leckband G., Abbadi A., Snowdon R.J. (2015). Genome-wide association mapping unravels the genetic control of seed germination and vigor in Brassica napus. Front. Plant Sci..

[43] Xin X., Lin X.-H., Zhou Y.-C., Chen X.-L., Liu X., Lu X.-X. (2011). Proteome analysis of maize seeds: The effect of artificial ageing. Physiol. Plant..

[44] Smolikova G.N., Shavarda A.L., Alekseichuk I.V., Chantseva V.V., Medvedev S.S. (2016). The metabolomic approach to the assessment of cultivar specificity of *Brassica napus* L. seeds. Russ. J. Genet. Appl. Res..

[45] Narula K., Sinha A., Haider T., Chakraborty N., Chakraborty S., Salekdeh G.H. (2016). Seed Proteomics: An overview. Agricultural Proteomics Volume 1.

[46] Min C.W., Gupta R., Kim S.W., Lee S.E., Kim Y.C., Bae D.W., Han W.Y., Lee B.W., Ko J.M., Agrawal G.K. (2015). Comparative biochemical and proteomic analyses of soybean seed cultivars differing in protein and oil content. J. Agric. Food Chem..

[47] Meyer L.J., Gao J., Xu D., Thelen J.J. (2012). Phosphoproteomic analysis of seed maturation in arabidopsis, rapeseed, and soybean. Plant Physiol..

[48] Larance M., Lamond A.I. (2015). Multidimensional proteomics for cell biology. Nat. Rev. Mol. Cell Biol..

[49] Plomion C., Meddour H., Kohler A., Jacob D., Bastien C., Dreyer E., De Daruvar A., Guehl J., Schmitter J., Martin F. (2006). Mapping the proteome of poplar and application to the discovery of drought-stress responsive proteins. Proteomics.

[50] Bantscheff M., Schirle M. (2007). Quantitative mass spectrometry in proteomics: A critical review. Anal. Bioanal. Chem..

[51] Jimenez-lopez J.C., Foley R.C., Brear E., Clarke V.C., Lima-cabello E., Florido J.F., Singh K.B., Alché J.D., Smith P.M.C. (2018). Characterization of narrow-leaf lupin (*Lupinus angustifolius* L.) recombinant major allergen IgE-binding proteins and the natural β -conglutin counterparts in sweet lupin seed species. Food Chem..

[52] Luthria D.L., Maria John K.M., Marupaka R., Natarajan S. (2018). Recent update on methodologies for extraction and analysis of soybean seed proteins. J. Sci. Food Agric..

[53] Alvarez S., Roy Choudhury S., Sivagnanam K., Hicks L.M., Pandey S. (2015). Quantitative proteomics analysis of *Camelina sativa* seeds overexpressing the AGG3 gene to identify the proteomic basis of increased yield and stress tolerance. J. Proteome Res..

[54] Hart-Smith G., Reis R.S., Waterhouse P.M., Wilkins M.R. (2017). Improved quantitative plant proteomics via the combination of targeted and untargeted data acquisition. Front. Plant Sci..

[55] Mamontova T., Afonin A.M., Ihling C., Soboleva A., Lukasheva E., Sulima A.S., Shtark O.Y., Akhtemova G.A., Povydysh M.N., Sinz A. (2019). Profiling of seed proteome in pea (*Pisum sativum* L.) lines characterized with high and low responsivity to combined inoculation with nodule bacteria and arbuscular mycorrhizal fungi. Molecules.

[56] Soboleva A., Schmidt R., Vikhnina M., Grishina T., Frolov A. (2017). Maillard Proteomics: Opening new pages. Int. J. Mol. Sci..

[57] Jorrín-Novo J.V., Pascual J., Sánchez-Lucas R., Romero-Rodríguez M.C., Rodríguez-Ortega M.J., Lenz C., Valledor L. (2015). Fourteen years of plant proteomics reflected in proteomics: Moving from model species and 2DE-based approaches to orphan species and gel-free platforms. Proteomics.

[58] Catherman A.D., Skinner O.S., Kelleher N.L. (2014). Top down proteomics: Facts and perspectives. Biochem. Biophys. Res. Commun..

[59] Chmelik J., Zidkova J., Rehulka P., Petry-Podgorska I., Bobalova J. (2009). Influence of different proteomic protocols on degree of high-coverage identification of nonspecific lipid transfer protein 1 modified during malting. Electrophoresis.

[60] Gillet L.C., Leitner A., Aebersold R. (2016). Mass spectrometry applied to bottom-up proteomics: Entering the high-throughput era for hypothesis testing. Annu. Rev. Anal. Chem..

[61] Mørtz E., O’Connor P.B., Roepstorff P., Kelleher N.L., Wood T.D., McLafferty F.W., Mann M. (1996). Sequence tag identification of intact proteins by matching tanden mass spectral data against sequence data bases. Proc. Natl. Acad. Sci. USA.

[62] Hummel M., Wigger T., Brockmeyer J. (2015). Characterization of mustard 2S albumin allergens by bottom-up, middle-down and top-down proteomics: A consensus set of isoforms of Sin a 1. J. Proteome Res..

[63] Mamontova T., Lukasheva E., Mavropolo-Stolyarenko G., Proksch C., Bilova T., Kim A., Babakov V., Grishina T., Hoehenwarter W., Medvedev S. (2018). Proteome map of pea (*Pisum sativum* L.) embryos containing different amounts of residual chlorophylls. Int. J. Mol. Sci..

[64] Bose U., Broadbent J.A., Byrne K., Hasan S., Howitt C.A., Colgrave M.L. (2019). Optimisation of protein extraction for in-depth profiling of the cereal grain proteome. J. Proteomics.

[65] Nilsen T.W., Graveley B.R. (2010). Expansion of the eukaryotic proteome by alternative splicing. Nature.

[66] Bourgeois M., Jacquin F., Cassecuelle F., Savois V., Belghazi M., Aubert G., Quillien L., Huart M., Marget P., Burstin J. (2011). A PQL (protein quantity loci) analysis of mature pea seed proteins identifies loci determining seed protein composition. Proteomics.

[67] Natarajan S.S., Krishnan H.B., Lakshman S., Garrett W.M. (2009). An efficient extraction method to enhance analysis of low abundant proteins from soybean seed. Anal. Biochem..

[68] Kim Y.J.Y.C., Wang Y., Gupta R., Kim S.T.S.W., Min C.W., Kim Y.J.Y.C., Park K.H., Agrawal G.K., Rakwal R., Choung M.-G.G. (2015). Protamine sulfate precipitation method depletes abundant plant seed-storage proteins: A case study on legume plants. Proteomics.

[69] Min C.W., Park J., Bae J.W., Agrawal G.K., Rakwal R., Kim Y., Yang P., Kim S.T., Gupta R. (2020). In-Depth Investigation of low-abundance proteins in matured and filling stages seeds of glycine max employing a combination of protamine sulfate precipitation and TMT-Based quantitative proteomic analysis. Cells.

[70] Krishnan H.B., Oehrle N.W., Natarajan S.S. (2009). A rapid and simple procedure for the depletion of abundant storage proteins from legume seeds to advance proteome analysis: A case study using *Glycine max*. Proteomics.

[71] Righetti P.G., Boschetti E. (2020). Low-abundance plant protein enrichment with peptide libraries to enlarge proteome coverage and related applications. Plant Sci..

[72] Kretzschmar F.K., Doner N.M., Krawczyk H.E., Scholz P., Schmitt K., Valerius O., Braus G.H., Mullen R.T., Ischebeck T. (2020). Identification of low-abundance lipid droplet proteins in seeds and seedlings. Plant Physiol..

[73] Du C., Liu A., Niu L., Cao D., Liu H., Wu X., Wang W. (2019). Proteomic identification of lipid-bodies-associated proteins in maize seeds. Acta Physiol. Plant..

[74] Tan B.C., Lim Y.S., Lau S. (2017). Proteomics in commercial crops: An overview. J. Proteomics.

[75] Dawod M., Arvin N.E., Kennedy R.T. (2017). Recent advances in protein analysis by capillary and microchip electrophoresis. Analyst.

[76] Kota U., Goshe M.B. (2011). Advances in qualitative and quantitative plant membrane proteomics. Phytochemistry.

[77] Rogowska-Wrzesinska A., Le Bihan M.-C., Thaysen-Andersen M., Roepstorff P. (2013). 2D gels still have a niche in proteomics. J. Proteomics.

[78] Ostergaard O., Finnie C., Laugesen S., Roepstorff P., Svennson B. (2004). Proteome analysis of barley seeds: Identification of major proteins from two-dimensional gels (pI 4-7). Proteomics.

[79] Rabilloud T., Lelong C. (2011). Two-dimensional gel electrophoresis in proteomics: A tutorial. J. Proteomics.

[80] Xu X., Fan R., Zheng R., Li C., Yu D. (2011). Proteomic analysis of seed germination under salt stress in soybeans. J. Zhejiang Univ. Sci. B.

[81] Rabilloud T., Chevallet M., Luche S., Lelong C. (2010). Two-dimensional gel electrophoresis in proteomics: Past, present and future. J. Proteomics.

[82] Gallardo K., Kurt C., Thompson R., Ochatt S. (2006). *In vitro* culture of immature *M. truncatula* grains under conditions permitting embryo development comparable to that observed *in vivo*. Plant Sci..

[83] Magdeldin S., Enany S., Yoshida Y., Xu B., Zhang Y., Zureena Z., Lokamani I., Yaoita E., Yamamoto T. (2014). Basics and recent advances of two dimensional- polyacrylamide gel electrophoresis. Clin. Proteomics.

[84] Rune M. (2006). Mass Spectrometry Data Analysis in Proteomics.

[85] Friedman D.B., Hoving S., Westermeier R. (2009). Chapter 30 isoelectric focusing and two-dimensional gel electrophoresis. Methods in Enzymology.

[86] Lee P.Y., Saraygord-Afshari N., Low T.Y. (2020). The evolution of two-dimensional gel electrophoresis—From proteomics to emerging alternative applications. J. Chromatogr. A.

[87] Jorrin-Novo J.V., Komatsu S., Sanchez-Lucas R., Rodríguez de Francisco L.E. (2019). Gel electrophoresis-based plant proteomics: Past, present, and future. Happy 10th anniversary Journal of Proteomics!. J. Proteomics.

[88] Meleady P. (2018). Two-Dimensional gel electrophoresis and 2D-DIGE. Difference Gel Electrophoresis. Methods in Molecular Biology.

[89] Isaacson T., Damasceno C.M.B., Saravanan R.S., He Y., Catalá C., Saladié M., Rose J.K.C. (2006). Sample extraction techniques for enhanced proteomic analysis of plant tissues. Nat. Protoc..

[90] Wang W., Vignani R., Scali M., Cresti M. (2006). A universal and rapid protocol for protein extraction from recalcitrant plant tissues for proteomic analysis. Electrophoresis.

[91] Wang X., Li X., Deng X., Han H., Shi W., Li Y. (2007). A protein extraction method compatible with proteomic analysis for the euhalophyte Salicornia europaea. Electrophoresis.

[92] Soboleva A., Vikhnina M., Grishina T., Frolov A. (2017). Probing protein glycation by chromatography and mass spectrometry: Analysis of glycation adducts. Int. J. Mol. Sci..

[93] Antonova K., Vikhnina M., Soboleva A., Mehmood T., Heymich M., Leonova T., Bankin M., Lukasheva E., Gensberger-Reigl S., Medvedev S. (2019). Analysis of chemically labile glycation adducts in seed proteins: Case study of methylglyoxal-derived hydroimidazolone 1 (MG-H1). Int. J. Mol. Sci..

[94] Damerval C., de Dominique V., Zivy M., Thiellement H. (1986). Technical improvements in two-dimensional electrophoresis increase the level of genetic variation detected in wheat-seedling proteins. Electrophoresis.

[95] Capriotti A.L., Cavaliere C., Piovesana S., Stampachiacchiere S., Ventura S., Zenezini Chiozzi R., Laganà A. (2015). Characterization of quinoa seed proteome combining different protein precipitation techniques: Improvement of knowledge of nonmodel plant proteomics. J. Sep. Sci..

[96] Natarajan S., Xu C., Caperna T.J., Garrett W.M. (2005). Comparison of protein solubilization methods suitable for proteomic analysis of soybean seed proteins. Anal. Biochem..

[97] Fu Y., Zhang H., Mandal S.N., Wang C., Chen C., Ji W. (2016). Quantitative proteomics reveals the central changes of wheat in response to powdery mildew. J. Proteomics.

[98] Baslam M., Kaneko K., Mitsui T., Jorrin-Novo J.V., Valledor L., Castillejo M.A., Rey M.-D. (2020). iTRAQ-based proteomic analysis of rice grains. Plant Proteomics: Methods and Protocols, Methods in Molecular Biology.

[99] Görg A., Weiss W. (2000). Two-dimensional electrophoresis with immobilized pH gradients. Proteome Research: Two-Dimensional Gel Electrophoresis and Identification Methods.

[100] Vâlcu C.-M., Schlink K. (2006). Reduction of proteins during sample preparation and two-dimensional gel electrophoresis of woody plant samples. Proteomics.

[101] Negri A.S., Prinsi B., Scienza A., Morgutti S., Cocucci M., Espen L. (2008). Analysis of grape berry cell wall proteome: A comparative evaluation of extraction methods. J. Plant Physiol..

[102] Dong Y., Wang Q., Zhang L., Du C., Xiong W. (2015). Dynamic Proteomic characteristics and network integration revealing key proteins for two kernel tissue developments in popcorn. PLoS ONE.

[103] Ranjbar Sistani N., Kaul H., Desalegn G., Wienkoop S. (2017). Rhizobium impacts on seed productivity, quality, and protection of *Pisum sativum* upon disease stress caused by didymella pinodes: Phenotypic, proteomic, and metabolomic traits. Front. Plant Sci..

[104] Cremer F., van de Walle C. (1985). Method for extraction of proteins from green plant tissues for two-dimensional polyacrylamide gel electrophoresis. Anal. Biochem..

[105] Milkovska-Stamenova S., Schmidt R., Frolov A., Birkemeyer C. (2015). GC-MS method for the quantitation of carbohydrate intermediates in glycation systems. J. Agric. Food Chem..

[106] Frolov A., Schmidt R., Spiller S., Greifenhagen U., Hoffmann R. (2014). Arginine-derived advanced glycation end products generated in peptide–glucose mixtures during boiling. J. Agric. Food Chem..

[107] Greifenhagen U., Frolov A., Blüher M., Hoffmann R. (2016). Plasma Proteins modified by advanced glycation end products (AGEs) reveal site-specific susceptibilities to glycemic control in patients with type 2 diabetes. J. Biol. Chem..

[108] Han C., Yin X., He D., Yang P. (2013). Analysis of Proteome profile in germinating soybean seed, and its comparison with rice showing the styles of reserves mobilization in different crops. PLoS ONE.

[109] Nishimura N., Tsuchiya W., Moresco J.J., Hayashi Y., Satoh K., Kaiwa N., Irisa T., Kinoshita T., Schroeder J.I., Yates J.R. (2018). Control of seed dormancy and germination by DOG1-AHG1 PP2C phosphatase complex *via* binding to heme. Nat. Commun..

[110] Lorenz C., Brandt S., Borisjuk L., Rolletschek H., Heinzel N., Tohge T., Fernie A.R., Braun H.-P., Hildebrandt T.M. (2018). The role of persulfide metabolism during *Arabidopsis* seed development under light and dark conditions. Front. Plant Sci..

[111] Wang W., Vignani R., Scali M., Sensi E., Tiberi P., Cresti M. (2004). Removal of lipid contaminants by organic solvents from oilseed protein extract prior to electrophoresis. Anal. Biochem..

[112] Murad A.M., Rech E.L. (2012). NanoUPLC-MSE proteomic data assessment of soybean seeds using the Uniprot database. BMC Biotechnol..

[113] Satour P., Youssef C., Châtelain E., Vu B.L., Teulat B., Job C., Job D., Montrichard F. (2018). Patterns of protein carbonylation during *Medicago truncatula* seed maturation. Plant. Cell Environ..

[114] Tang H., Ming Z., Liu R., Xiong T., Grevelding C.G., Dong H., Jiang M. (2013). Development of Adult worms and granulomatous pathology are collectively regulated by *t*- and *b*-cells in mice infected with *Schistosoma japonicum*. PLoS ONE.

[115] Rakwal R., Hayashi G., Shibato J., Deepak S.A., Gundimeda S., Simha U., Padmanaban A., Gupta R., Han S., Kim S.T. (2018). Progress toward rice seed OMICS in Low-level gamma radiation environment in iitate Village, Fukushima. J. Hered..

[116] Pinheiro C., Sergeant K., Machado C.M., Renaut J., Ricardo C.P. (2013). Two Traditional maize inbred lines of contrasting technological abilities are discriminated by the seed flour proteome. J. Proteome Res..

[117] Niu L., Ding H., Zhang J., Wang W. (2019). Proteomic analysis of starch biosynthesis in maize seeds. Starch - Stärke.

[118] Neuhoff V., Arold N., Taube D., Ehrhardt W. (1988). Dependence of Particle and fiber properties. Electrophoresis.

[119] Frolov A., Blüher M., Hoffmann R. (2014). Glycation sites of human plasma proteins are affected to different extents by hyperglycemic conditions in type 2 diabetes mellitus. Anal. Bioanal. Chem..

[120] Romero-Rodríguez M.C., Jorrín-Novo J.V., Castillejo M.A. (2019). Toward characterizing germination and early growth in the non-orthodox forest tree species Quercus ilex through complementary gel and gel-free proteomic analysis of embryo and seedlings. J. Proteomics.

[121] Chevalier F. (2010). Standard dyes for total protein staining in gel-based proteomic analysis. Materials.

[122] Mortz E., Krogh T.N., Vorum H., Görg A. (2001). Improved silver staining protocols for high sensitivity protein identification using matrix-assisted laser desorption/ionization-time of flight analysis. Proteomics.

[123] Chevallet M., Luche S., Strub J., Van Dorsselaer A., Rabilloud T. (2008). Sweet silver: A formaldehyde-free silver staining using aldoses as developing agents, with enhanced compatibility with mass spectrometry. Proteomics.

[124] Puumalainen T.J., Puustinen A., Poikonen S., Turjanmaa K., Palosuo T., Vaali K. (2015). Proteomic identification of allergenic seed proteins, napin and cruciferin, from cold-pressed rapeseed oils. Food Chem..

[125] Berggren K., Chernokalskaya E., Steinberg T.H., Kemper C., Lopez M.F., Diwu Z., Haugland R.P., Patton W.F. (2000). Background-free, high sensitivity staining of proteins in one- and two-dimensional sodium dodecyl sulfate-polyacrylamide gels using a luminescent ruthenium complex. Electrophoresis.

[126] Rabilloud T., Strub J., Luche S., Van Dorsselaer A., Lunardi J. (2001). A comparison between Sypro Ruby and ruthenium II tris (bathophenanthroline disulfonate) as. Proteomics.

[127] Zhou G., Li H., DeCamp D., Chen S., Shu H., Gong Y., Flaig M., Gillespie J.W., Hu N., Taylor P.R. (2002). 2D Differential In-gel electrophoresis for the identification of esophageal scans cell cancer-specific protein markers. Mol. Cell. Proteomics.

[128] Romero-Rodriguez M., Abril N., Sánchez-Lucas R., Jorrin Novo J. (2015). V Multiplex staining of 2-DE gels for an initial phosphoproteome analysis of germinating seeds and early grown seedlings from a non-orthodox specie: *Quercus ilex* L. subsp. ballota [Desf.] Samp. Front. Plant Sci..

[129] Klose J., Kobalz U. (1995). Two-dimensional electrophoresis of proteins: An updated protocol and implications for a functional analysis of the genome. Electrophoresis.

[130] Gygi S.P., Corthals G.L., Zhang Y., Rochon Y., Aebersold R. (2000). Evaluation of two-dimensional gel electrophoresis-based proteome analysis technology. Proc. Natl. Acad. Sci. USA.

[131] Chassaigne H., Trégoat V., Nørgaard J.V., Maleki S.J., van Hengel A.J. (2009). Resolution and identification of major peanut allergens using a combination of fluorescence two-dimensional differential gel electrophoresis, Western blotting and Q-TOF mass spectrometry. J. Proteomics.

[132] Jorrin-Novo J.V., Jorrin-Novo J.V., Komatsu S., Weckwerth W., Wienkoop S. (2014). Plant proteomics methods and protocols. Plant Proteomics.

[133] Scippa G.S., Rocco M., Ialicicco M., Trupiano D., Viscosi V., Di Michele M., Arena S., Chiatante D., Scaloni A. (2010). The proteome of lentil *(Lens culinaris* Medik.) seeds: Discriminating between landraces. Electrophoresis.

[134] Talamo F., D’Ambrosio C., Arena S., Del Vecchio P., Ledda L., Zehender G., Ferrara L., Scaloni A. (2003). Proteins from bovine tissues and biological fluids: Defining a reference electrophoresis map for liver, kidney, muscle, plasma and red blood cells. Proteomics.

[135] Schönberg A., Rödiger A., Mehwald W., Galonska J., Christ G., Helm S., Thieme D., Majovsky P., Hoehenwarter W., Baginsky S. (2017). Identification of STN7/STN8 kinase targets reveals connections between electron transport, metabolism and gene expression. Plant J..

[136] Rosenfeld J., Capdevielle J., Guillemot J.C., Ferrara P. (1992). In-gel digestion of proteins for internal analysis after one- or two-dimensional gel electrophoresis. Anal. Biochem..

[137] Blum H., Beier H., Gross H.J. (1987). Improved silver staining of plant proteins, RNA and DNA in polyacrylamide gels. Electrophoresis.

[138] Ogura T., Ogihara J., Sunairi M., Takeishi H., Aizawa T., Olivos-Trujillo M.R., Maureira-Butler I.J., Salvo-Garrido H.E. (2014). Proteomic characterization of seeds from yellow lupin (*Lupinus luteus* L.). Proteomics.

[139] Gomes L.S., Senna R., Sandim V., Silva-Neto M.A.C., Perales J.E.A., Zingali R.B., Soares M.R., Fialho E. (2014). Four conventional soybean [*Glycine max* (L.) Merrill] seeds exhibit different protein profiles as revealed by proteomic analysis. J. Agric. Food Chem..

[140] Min C.W., Lee S.H., Cheon Y.E., Han W.Y., Ko J.M., Kang H.W., Kim Y.C., Agrawal G.K., Rakwal R., Gupta R. (2017). In-depth proteomic analysis of *Glycine max* seeds during controlled deterioration treatment reveals a shift in seed metabolism. J. Proteomics.

[141] Swart C., Martínez-Jaime S., Gorka M., Zander K., Graf A. (2018). Hit-gel: Streamlining *in-gel* protein digestion for high-throughput proteomics experiments. Sci. Rep..

[142] Mallick P., Schirle M., Chen S.S., Flory M.R., Lee H., Martin D., Ranish J., Raught B., Schmitt R., Werner T. (2007). Computational prediction of proteotypic peptides for quantitative proteomics. Nat. Biotechnol..

[143] Vandemoortele G., Staes A., Gonnelli G., Samyn N., De Sutter D., Vandermarliere E., Timmerman E., Gevaert K., Martens L., Eyckerman S. (2016). An extra dimension in protein tagging by quantifying universal proteotypic peptides using targeted proteomics. Sci. Rep..

[144] Keerthikumar S., Mathivanan S. (2017). Proteotypic Peptides and Their Applications. Methods in Molecular Biology.

[145] Bhattacharya A., Dubitzky W., Wolkenhauer O., Cho K.-H., Yokota H. (2013). Proteotypic Peptides. Encyclopedia of Systems Biology.

[146] Clauser K.R., Baker P., Burlingame A.L. (1999). Role of accurate mass measurement (10 ppm ) in protein identification strategies employing MS or MS/MS and database searching. Anal. Chem..

[147] Çakir B., Gülseren İ. (2019). Identification of novel proteins from black cumin seed meals based on 2D gel electrophoresis and MALDI-TOF/TOF-MS analysis. Plant Foods Hum. Nutr..

[148] Wu W.W., Wang G., Baek S.J., Shen R.-F. (2006). Comparative study of three proteomic quantitative methods, DIGE, cICAT, and iTRAQ, using 2D gel- or LC−MALDI TOF/TOF. J. Proteome Res..

[149] Wu W., Dai R.-T., Bendixen E. (2019). Comparing SRM and SWATH methods for quantitation of bovine muscle proteomes. J. Agric. Food Chem..

[150] Que Y., Xu L., Lin J., Ruan M., Zhang M., Chen R. (2011). Differential protein expression in sugarcane during sugarcane- sporisorium scitamineum interaction revealed by 2-DE and MALDI-TOF-TOF/MS. Comp. Funct. Genomics.

[151] Vadivel A.K.A. (2015). Gel-based proteomics in plants: Time to move on from the tradition. Front. Plant Sci..

[152] Maaß S., Becher D. (2016). Methods and applications of absolute protein quantification in microbial systems. J. Proteomics.

[153] Nedenskov Jensen K., Jessen F., Jørgensen B.M. (2008). Multivariate Data analysis of two-dimensional gel electrophoresis protein patterns from few samples. J. Proteome Res..

[154] Marengo E., Robotti E., Antonucci F., Cecconi D., Campostrini N., Righetti P.G. (2005). Numerical approaches for quantitative analysis of two-dimensional maps: A review of commercial software and home-made systems. Proteome Sci..

[155] Voss T., Haberl P. (2000). Observations on the reproducibility and matching efficiency of two-dimensional electrophoresis gels: Consequences for comprehensive data analysis. Electrophoresis.

[156] Arentz G., Weiland F., Oehler M.K., Hoffmann P. (2015). State of the art of 2D DIGE. Proteomics Clin. Appl..

[157] Beckett P. (2012). The Basics of 2D DIGE. Difference Gel Electrophoresis (DIGE). Methods in Molecular Biology (Methods and Protocols).

[158] Morgan M.E., Minden J.S. (1997). Difference gel electrophoresis: A single gel method for detecting changes in protein extracts. Electrophoresis.

[159] TripleTOF® 6600 System. https://sciex.com/products/mass-spectrometers/qtof-systems/tripletof-systems/tripletof-6600-system.

[160] Krokhin O.V., Ens W., Standing K.G. (2005). MALDI QqTOF MS Combined with off-line HPLC for Characterization of protein primary structure and post-translational modifications. J. Biomol. Tech..

[161] Köcher T., Pichler P., Swart R., Mechtler K. (2012). Analysis of protein mixtures from whole-cell extracts by single-run nanoLC-MS/MS using ultralong gradients. Nat. Protoc..

[162] Burrieza H.P., Rizzo A.J., Moura Vale E., Silveira V., Maldonado S. (2019). Shotgun proteomic analysis of quinoa seeds reveals novel lysine-rich seed storage globulins. Food Chem..

[163] Frolov A., Henning A., Böttcher C., Tissier A., Strack D., Bo C., Tissier A., Strack D., Böttcher C., Tissier A. (2013). An UPLC-MS/MS Method for the simultaneous identification and quantitation of cell wall phenolics in *Brassica napus* seeds. J. Agric. Food Chem..

[164] Matthiesen R., Bunkenborg J. (2020). Introduction to mass spectrometry-based proteomics. Methods in Molecular Biology.

[165] Vissers J.P.C., Chervet J.P., Salzmann J.P. (1996). Sodium dodecyl sulphate removal from tryptic digest samples for on-line capillary liquid. J. Mass Spectrom..

[166] Cole R.B. (2012). Electrospray and MALDI Mass Spectrometry: Fundamentals, Instrumentation, Practicalities, and Biological Applications.

[167] Chen E.I., Cociorva D., Norris J.L., Yates J.R. (2007). Optimization of mass spectrometry-compatible surfactants for shotgun proteomics. J. Proteome Res..

[168] Lin Y., Jiang H., Yan Y., Peng B., Chen J., Lin H., Liu Z. (2012). Shotgun analysis of membrane proteomes by an improved SDS-assisted sample preparation method coupled with liquid chromatography–tandem mass spectrometry. J. Chromatogr. B.

[169] Bilova T., Greifenhagen U., Paudel G., Lukasheva E., Brauch D., Osmolovskaya N., Tarakhovskaya E., Balcke G.U., Tissier A., Vogt T. (2016). Glycation of plant proteins under environmental stress—Methodological approaches, potential mechanisms and biological role. Abiotic and Biotic Stress in Plants—Recent Advances and Future Perspectives.

[170] Greifenhagen U., Frolov A., Blüher M., Hoffmann R. (2016). Site-specific analysis of advanced glycation end products in plasma proteins of type 2 diabetes mellitus patients. Anal. Bioanal. Chem..

[171] Bilova T., Lukasheva E., Brauch D., Greifenhagen U., Paudel G., Tarakhovskaya E., Frolova N., Mittasch J., Balcke G.U.G.U., Tissier A. (2016). A Snapshot of the plant glycated proteome: Structural, functional and mechanistic aspect. J. Biol. Chem..

[172] Takemori N., Takemori A., Ishizaki J., Hasegawa H. (2014). Enzymatic protein digestion using a dissolvable polyacrylamide gel and its application to mass spectrometry-based proteomics. J. Chromatogr. B.

[173] Kalli A., Smith T., Sweredoski M.J., Hess S. (2013). Evaluation and optimization of mass spectrometric settings during data-dependent acquisition mode: Focus on LTQ-orbitrap mass analyzers. J. Proteome Res..

[174] Robinson A.E., Binek A., Venkatraman V., Searle B.C., Holewinski R.J., Rosenberger G., Parker S.J., Basisty N., Xie X., Lund P.J. (2020). Lysine and arginine protein post-translational modifications by enhanced dia libraries: Quantification in murine liver disease. J. Proteome Res..

[175] Majovsky P., Naumann C., Lee C., Lassowskat I., Trujillo M., Dissmeyer N., Hoehenwarter W. (2014). Targeted Proteomics analysis of protein degradation in plant signaling on an LTQ-orbitrap mass spectrometer. J. Proteome Res..

[176] Antonets K.S., Belousov M.V., Sulatskaya A.I., Belousova M.E., Kosolapova A.O., Sulatsky M.I., Andreeva E.A., Zykin P.A., Malovichko Y.V., Shtark O.Y. (2020). Accumulation of storage proteins in plant seeds is mediated by amyloid formation. PLoS Biol..

[177] Paudel G., Bilova T., Schmidt R., Greifenhagen U., Berger R., Tarakhovskaya E., Stöckhardt S., Balcke G.U., Humbeck K., Brandt W. (2016). Osmotic stress is accompanied by protein glycation in *Arabidopsis thaliana*. J. Exp. Bot..

[178] Hart-Smith G. (2020). Combining Targeted and untargeted data acquisition to enhance quantitative plant proteomics experiments. Plant Proteomics. Methods in Molecular Biology.

[179] Juarez-Escobar J., Elizalde-Contreras J.M., Loyola-Vargas V.M., Ruiz-May E. (2020). A Phosphoproteomic analysis pipeline for peels of tropical fruits. Plant Proteomics. Methods in Molecular Biology.

[180] Wilm M. (2009). Quantitative proteomics in biological research. Proteomics.

[181] Ćeranić A., Doppler M., Büschl C., Parich A., Xu K., Koutnik A., Bürstmayr H., Lemmens M., Schuhmacher R. (2020). Preparation of uniformly labelled 13C- and 15N-plants using customised growth chambers. Plant Methods.

[182] Liu W., Li L., Zhang Z., Dong M., Jin W. (2020). iTRAQ-based quantitative proteomic analysis of transgenic and non-transgenic maize seeds. J. Food Compos. Anal..

[183] Chen L., Wang Z., Li M., Ma X., Tian E., Sun A., Yin Y. (2018). Analysis of the natural dehydration mechanism during middle and late stages of wheat seeds development by some physiological traits and iTRAQ-based proteomic. J. Cereal Sci..

[184] Nelson C.J., Hegeman A.D., Harms A.C., Sussman M.R. (2006). A Quantitative analysis of arabidopsis plasma membrane using trypsin-catalyzed 18 O labeling. Mol. Cell. Proteomics.

[185] Picotti P., Bodenmiller B., Aebersold R. (2013). Proteomics meets the scientific method. Nat. Methods.

[186] Wiśniewski J.R., Zougman A., Nagaraj N., Mann M. (2009). Universal sample preparation method for proteome analysis. Nat. Methods.

[187] George I.S., Fennell A.Y., Haynes P.A. (2015). Protein identification and quantification from riverbank grape, *Vitis riparia*: Comparing SDS-PAGE and FASP-GPF techniques for shotgun proteomic analysis. Proteomics.

[188] Zhang N., Zhang H.-J., Sun Q.-Q., Cao Y.-Y., Li X., Zhao B., Wu P., Guo Y.-D. (2017). Proteomic analysis reveals a role of melatonin in promoting cucumber seed germination under high salinity by regulating energy production. Sci. Rep..

[189] Lu X., Zhu H. (2005). Tube-gel digestion. Mol. Cell. Proteomics.

[190] Fischer R., Kessler B.M. (2015). Gel-aided sample preparation (GASP)—A simplified method for gel-assisted proteomic sample generation from protein extracts and intact cells. Proteomics.

[191] Muñoz-Talavera A., Gómez-Lim M.Á., Salazar-Olivo L.A., Reinders J., Lim K., Escobedo-Moratilla A., López-Calleja A.C., Islas-Carbajal M.C., Rincón-Sánchez A.R. (2019). Expression of the biologically active insulin analog SCI-57 in *Nicotiana benthamiana*. Front. Pharmacol..

[192] Strader M.B., Tabb D.L., Hervey W.J., Pan C., Hurst G.B. (2006). Efficient and specific trypsin digestion of microgram to nanogram quantities of proteins in organic−aqueous solvent systems. Anal. Chem..

[193] Zhang Y., Fonslow B.R., Shan B., Baek M.-C., Yates J.R. (2013). Protein Analysis by shotgun/bottom-up proteomics. Chem. Rev..

[194] Wang W.-Q., Jensen O.N., Møller I.M., Hebelstrup K.H., Rogowska-Wrzesinska A. (2018). Evaluation of sample preparation methods for mass spectrometry-based proteomic analysis of barley leaves. Plant Methods.

[195] Kołodziejczyk I., Dzitko K., Szewczyk R., Posmyk M.M. (2016). Exogenous melatonin expediently modifies proteome of maize (*Zea mays* L.) embryo during seed germination. Acta Physiol. Plant..

[196] Lin Y., Huo L., Liu Z., Li J., Liu Y., He Q., Wang X., Liang S. (2013). Sodium laurate, a novel protease- and mass spectrometry-compatible detergent for mass spectrometry-based membrane proteomics. PLoS ONE.

[197] Norris J.L., Porter N.A., Caprioli R.M. (2003). Mass Spectrometry of intracellular and membrane proteins using cleavable detergents. Anal. Chem..

[198] Zakowick H., Schagat T., Yoder D., Niles A.L. (2008). Measuring cell health and viability sequentially by same-well multiplexing using the GloMax^®^-Multi Detection System. Promega Notes.

[199] (2015). Technical Bulletin: ProteaseMAX(TM) Surfactant, Trypsin Enhancer.

[200] Li M., Powell M.J., Razunguzwa T.T., O’doherty G.A. (2010). A general approach to anionic acid-labile surfactants with tunable properties. J. Org. Chem..

[201] Ross A.R.S., Lee P.J., Smith D.L., Langridge J.I., Whetton A.D., Gaskell S.J. (2002). Identification of proteins from two-dimensional polyacrylamide gels using a novel acid-labile surfactant. Proteomics.

[202] Jagadeeshaprasad M.G., Batkulwar K.B., Meshram N.N., Tiwari S., Korwar A.M., Unnikrishnan A.G., Kulkarni M.J. (2016). Targeted quantification of N-1-(carboxymethyl) valine and N-1-(carboxyethyl) valine peptides of *β*-hemoglobin for better diagnostics in diabetes. Clin. Proteomics.

[203] Kaspar-Schoenefeld S., Merx K., Jozefowicz A.M., Hartmann A., Seiffert U., Weschke W., Matros A., Mock H. (2016). Label-free proteome profiling reveals developmental-dependent patterns in young barley grains. J. Proteomics.

[204] Increase Analytical Accuracy LC/MS: Solvents, Blends, Standards, Surfactants; ThermoFisher Scientific, USA. https://beta-static.fishersci.com/content/dam/fishersci/en_US/documents/programs/scientific/brochures-and-catalogs/guides/lcms-solvents-guide.pdf.

[205] Waas M., Bhattacharya S., Chuppa S., Wu X., Jensen D.R.R., Omasits U., Wollscheid B., Volkman B.F., Noon K.R., Gundry R.L. (2014). Combine and conquer: Surfactants, solvents, and chaotropes for robust mass spectrometry based analyses of membrane proteins. Anal. Chem..

[206] Frolov A., Bilova T., Paudel G., Berger R., Balcke G.U.G.U., Birkemeyer C., Wessjohann L.A.L.A. (2017). Early responses of mature *Arabidopsis thaliana* plants to reduced water potential in the agar-based polyethylene glycol infusion drought model. J. Plant Physiol..

[207] Hogrebe A., von Stechow L., Bekker-Jensen D.B., Weinert B.T., Kelstrup C.D., Olsen J. (2018). V Benchmarking common quantification strategies for large-scale phosphoproteomics. Nat. Commun..

[208] Dam S., Thaysen-Andersen M., Stenkjær E., Lorentzen A., Roepstorff P., Packer N.H., Stougaard J. (2013). Combined N-glycome and N-glycoproteome analysis of the *Lotus japonicus* seed globulin fraction shows conservation of protein structure and glycosylation in legumes. J. Proteome Res..

[209] Canterbury J.D., Merrihew G.E., Maccoss M.J., Goodlett D.R., Shaffer S.A. (2014). Comparison of Data acquisition strategies on quadrupole ion trap instrumentation for shotgun proteomics. J. Am. Soc. Mass Spectrom..

[210] Frolov A., Hoffmann R. (2008). Analysis of Amadori peptides enriched by boronic acid affinity chromatography. Ann. N. Y. Acad. Sci..

[211] Manual 2D Quant Kit. https://www.gelifesciences.com/gehcls_images/GELS/RelatedContent/Files/1314729545976/litdoc28954714AE_20110830215136.pdf.

[212] Matamoros M.A., Kim A., Peñuelas M., Ihling C., Griesser E., Hoffmann R., Fedorova M., Frolov A., Becana M. (2018). Protein carbonylation and glycation in legume nodules. Plant Physiol..

[213] Plumb R.S., Johnson K.A., Rainville P., Smith B.W., Wilson I.D., Castro-Perez J.M., Nicholson J.K. (2006). UPLC/MSE; a new approach for generating molecular fragment information for biomarker structure elucidation. Rapid Commun. Mass Spectrom..

[214] Uvackova L., Skultety L., Bekesova S., McClain S., Hajduch M. (2013). The MSE-proteomic analysis of gliadins and glutenins in wheat grain identifies and quantifies proteins associated with celiac disease and baker’s asthma. J. Proteomics.

[215] Ludwig C., Gillet L., Rosenberger G., Amon S., Collins B.C., Aebersold R. (2018). Data-independent acquisition-based SWATH-MS for quantitative proteomics: A tutorial. Mol. Syst. Biol..

[216] Gillet L.C., Navarro P., Tate S., Ro H., Selevsek N., Reiter L., Bonner R., Aebersold R., Röst H., Selevsek N. (2012). Targeted data extraction of the MS / MS spectra generated by data-independent acquisition: A new concept for consistent and accurate proteome analysis. Mol. Cell. Proteomics.

[217] Zhu F.-Y., Chen M.-X., Su Y.-W., Xu X., Ye N.-H., Cao Y.-Y., Lin S., Liu T.-Y., Li H.-X., Wang G.-Q. (2016). SWATH-MS Quantitative analysis of proteins in the rice inferior and superior spikelets during grain filling. Front. Plant Sci..

[218] Bateman N.W., Goulding S.P., Shulman N.J., Gadok A.K., Szumlinski K.K., MacCoss M.J., Wu C.C. (2014). Maximizing Peptide identification events in proteomic workflows using data-dependent acquisition (DDA). Mol. Cell. Proteomics.

[219] Zhu W., Smith J.W., Huang C.-M. (2010). Mass spectrometry-based label-free quantitative proteomics. J. Biomed. Biotechnol..

[220] Nogueira F.C.S., Palmisano G., Soares E.L., Shah M., Soares A.A., Roepstorff P., Campos F.A.P., Domont G.B. (2012). Proteomic profile of the nucellus of castor bean (*Ricinus communis* L.) seeds during development. J. Proteomics.

[221] Fercha A., Capriotti A.L., Caruso G., Cavaliere C., Samperi R., Stampachiacchiere S., Laganà A. (2014). Comparative analysis of metabolic proteome variation in ascorbate-primed and unprimed wheat seeds during germination under salt stress. J. Proteomics.

[222] Gladilovich V., Greifenhagen U., Sukhodolov N., Selyutin A., Singer D., Thieme D., Majovsky P., Shirkin A., Hoehenwarter W., Bonitenko E. (2016). Immobilized metal affinity chromatography on collapsed Langmuir-Blodgett iron(III) stearate films and iron(III) oxide nanoparticles for bottom-up phosphoproteomics. J. Chromatogr. A.

[223] Kelstrup C.D., Bekker-jensen D.B., Arrey T.N., Hogrebe A., Harder A., Olsen J. (2018). V Performance evaluation of the Q exactive HF - X for shotgun proteomics. J. Proteome Res..

[224] Scheltema R.A., Hauschild J., Lange O., Hornburg D., Denisov E., Damoc E., Kuehn A., Makarov A., Mann M., Exactive Q. (2014). The Q exactive HF, a benchtop mass spectrometer with a pre-filter, high-performance quadrupole and an ultra-high-field orbitrap. Mol Cell Proteomics.

[225] Shi T., Su D., Liu T., Tang K., Camp D.G., Qian W.-J., Smith R.D. (2012). Advancing the sensitivity of selected reaction monitoring-based targeted quantitative proteomics. Proteomics.

[226] Nakamura K., Hirayama-Kurogi M., Ito S., Kuno T., Yoneyama T., Obuchi W., Terasaki T., Ohtsuki S. (2016). Large-scale multiplex absolute protein quantification of drug-metabolizing enzymes and transporters in human intestine, liver, and kidney microsomes by SWATH-MS: Comparison with MRM/SRM and HR-MRM/PRM. Proteomics.

[227] Peterson A.C., Russell J.D., Bailey D.J., Westphall M.S., Coon J.J. (2012). Parallel reaction monitoring for high resolution and high mass accuracy quantitative, targeted proteomics. Mol. Cell. Proteomics.

[228] Zargar S.M., Mahajan R., Nazir M., Nagar P., Kim S.T., Rai V., Masi A., Ahmad S.M., Shah R.A., Ganai N.A. (2017). Common bean proteomics: Present status and future strategies. J. Proteomics.

[229] Mergner J., Frejno M., List M., Papacek M., Chen X., Chaudhary A., Samaras P., Richter S., Shikata H., Messerer M. (2020). Mass-spectrometry-based draft of the *Arabidopsis* proteome. Nature.

[230] Rodríguez de Francisco L., Romero-Rodríguez M.C., Navarro-Cerrillo R.M., Miniño V., Perdomo O., Jorrín-Novo J.V. (2016). Characterization of the orthodox *Pinus occidentalis* seed and pollen proteomes by using complementary gel-based and gel-free approaches. J. Proteomics.

[231] Nguyen T.-P., Cueff G., Hegedus D.D., Rajjou L., Bentsink L. (2015). A role for seed storage proteins in *Arabidopsis* seed longevity. J. Exp. Bot..

[232] Li X., Wang W., Chen J. (2015). From pathways to networks: Connecting dots by establishing protein-protein interaction networks in signaling pathways using affinity purification and mass spectrometry. Proteomics.

[233] He D., Yang P. (2013). Proteomics of rice seed germination. Front. Plant Sci..

[234] Derouiche A., Cousin C., Mijakovic I. (2012). Protein phosphorylation from the perspective of systems biology. Curr. Opin. Biotechnol..

[235] Von Stechow L., Francavilla C., Olsen J.V. (2015). Recent findings and technological advances in phosphoproteomics for cells and tissues. Expert Rev. Proteomics.

[236] Yin X., Wang X., Komatsu S. (2018). Phosphoproteomics: Protein phosphorylation in regulation of seed germination and plant growth. Curr. Protein Pept. Sci..

[237] Mouzo D., Bernal J., López-Pedrouso M., Franco D., Zapata C. (2018). Advances in the biology of seed and vegetative storage proteins based on two-dimensional electrophoresis coupled to mass spectrometry. Molecules.

[238] Agrawal G.K., Thelen J.J. (2006). Large scale identification and quantitative profiling of phosphoproteins expressed during seed filling in oilseed rape. Mol. Cell. Proteomics.

[239] López-Pedrouso M., Alonso J., Zapata C. (2014). Evidence for phosphorylation of the major seed storage protein of the common bean and its phosphorylation-dependent degradation during germination. Plant Mol. Biol..

[240] Irar S., Oliveira E., Pagès M., Goday A. (2006). Towards the identification of late-embryogenic-abundant phosphoproteome in *Arabidopsis* by 2-DE and MS. Proteomics.

[241] Wan L., Ross A.R.S., Yang J., Hegedus D.D., Kermode A.R. (2007). Phosphorylation of the 12 S globulin cruciferin in wild-type and abi1-1 mutant A*rabidopsis thaliana* (thale cress) seeds. Biochem. J..

[242] Kuyama H., Toda C., Watanabe M., Tanaka K., Nishimura O. (2003). An efficient chemical method for dephosphorylation of phosphopeptides. Rapid Commun. Mass Spectrom..

[243] Kita K., Okumura N., Takao T., Watanabe M., Matsubara T., Nishimura O., Nagai K. (2006). Evidence for phosphorylation of rat liver glucose-regulated protein 58, GRP58/ERp57/ER-60, induced by fasting and leptin. FEBS Lett..

[244] Woo E.M., Fenyo D., Kwok B.H., Funabiki H., Chait B.T. (2008). Efficient identification of phosphorylation by mass spectrometric phosphopeptide fingerprinting. Anal. Chem..

[245] Bernal J., López-Pedrouso M., Franco D., Bravo S., García L., Zapata C. (2017). Identification and mapping of phosphorylated isoforms of the major storage protein of potato based on two- dimensional electrophoresis. Advances in Seed Biology.

[246] Sinha A., Haider T., Narula K., Ghosh S., Chakraborty N., Chakraborty S. (2020). Integrated seed proteome and phosphoproteome analyses reveal interplay of nutrient dynamics, carbon–nitrogen partitioning, and oxidative signaling in chickpea. Proteomics.

[247] Han C., Yang P. (2016). Two dimensional gel electrophoresis-based plant phosphoproteomics. Phospho-Proteomics. Methods in Molecular Biology.

[248] Guo G., Lv D., Yan X., Subburaj S., Ge P., Li X., Hu Y., Yan Y. (2012). Proteome characterization of developing grains in bread wheat cultivars (*Triticum aestivum* L.). BMC Plant Biol..

[249] Han C., Wang K., Yang P. (2014). Gel-Based Comparative phosphoproteomic analysis on rice embryo during germination. Plant Cell Physiol..

[250] Li M., Yin X., Sakata K., Yang P., Komatsu S. (2015). Proteomic analysis of phosphoproteins in the rice nucleus during the early stage of seed germination. J. Proteome Res..

[251] Wang Y., Tong X., Qiu J., Li Z., Zhao J., Hou Y., Tang L., Zhang J. (2017). A phosphoproteomic landscape of rice (*Oryza sativa*) tissues. Physiol. Plant..

[252] Forzani C., Carreri A., De La Fuente Van Bentem S., Lecourieux D., Lecourieux F., Hirt H. (2011). The *Arabidopsis* protein kinase Pto-interacting 1-4 is a common target of the oxidative signal-inducible 1 and mitogen-activated protein kinases. FEBS J..

[253] Chen Y., Hoehenwarter W. (2019). Rapid and reproducible phosphopeptide enrichment by tandem metal oxide affinity chromatography: Application to boron deficiency induced phosphoproteomics. Plant J..

[254] Chang I.F., Hsu J.L., Hsu P.H., Sheng W.A., Lai S.J., Lee C., Chen C.W., Hsu J.C., Wang S.Y., Wang L.Y. (2012). Comparative phosphoproteomic analysis of microsomal fractions of *Arabidopsis thaliana* and *Oryza sativa* subjected to high salinity. Plant Sci..

[255] Fíla J., Matros A., Radau S., Zahedi R.P., Čapková V., Mock H.-P., Honys D. (2012). Revealing phosphoproteins playing role in tobacco pollen activated in vitro. Proteomics.

[256] Kurdyukov D.A., Chernova E.N., Russkikh Y.V., Eurov D.A., Sokolov V.V., Bykova A.A., Shilovskikh V.V., Keltsieva O.A., Ubyivovk E.V., Anufrikov Y.A. (2017). Ni-functionalized submicron mesoporous silica particles as a sorbent for metal affinity chromatography. J. Chromatogr. A.

[257] Yeh T.-T., Ho M.-Y., Chen W.-Y., Hsu Y.-C., Ku W.-C., Tseng H.-W., Chen S.-T., Chen S.-F. (2019). Comparison of different fractionation strategies for in-depth phosphoproteomics by liquid chromatography tandem mass spectrometry. Anal. Bioanal. Chem..

[258] Vu L.D., Zhu T., Verstraeten I., van de Cotte B., Gevaert K., De Smet I. (2018). Temperature-induced changes in the wheat phosphoproteome reveal temperature-regulated interconversion of phosphoforms. J. Exp. Bot..

[259] Qiu J., Hou Y., Tong X., Wang Y., Lin H., Liu Q., Zhang W., Li Z., Nallamilli B.R., Zhang J. (2016). Quantitative phosphoproteomic analysis of early seed development in rice (*Oryza sativa* L.). Plant Mol. Biol..

[260] Li J., Silva-Sanchez C., Zhang T., Chen S., Li H. (2015). Phosphoproteomics technologies and applications in plant biology research. Front. Plant Sci..

[261] Marcus K. (2012). Quantitative Methods in Proteomics.

[262] Bindschedler L.V., Palmblad M., Cramer R. (2008). Hydroponic isotope labelling of entire plants (HILEP) for quantitative plant proteomics; an oxidative stress case study. Phytochemistry.

[263] Hebeler R., Oeljeklaus S., Reidegeld K.A., Eisenacher M., Stephan C., Sitek B., Stühler K., Meyer H.E., Sturre M.J.G., Dijkwel P.P. (2008). Study of early leaf senescence in arabidopsis thaliana by quantitative proteomics using reciprocal 14 N/ 15 N labeling and difference gel electrophoresis. Mol. Cell. Proteomics.

[264] Minkoff B.B., Stecker K.E., Sussman M.R. (2015). Rapid Phosphoproteomic effects of abscisic acid (ABA) on wild-type and aba receptor-deficient *A. thaliana* mutants. Mol. Cell. Proteomics.

[265] Lewandowska D., ten Have S., Hodge K., Tillemans V., Lamond A.I., Brown J.W.S. (2013). Plant SILAC: Stable-isotope labelling with amino acids of *Arabidopsis* seedlings for quantitative proteomics. PLoS ONE.

[266] Wong M.M., Bhaskara G.B., Wen T.-N., Lin W.-D., Nguyen T.T., Chong G.L., Verslues P.E. (2019). Phosphoproteomics of *Arabidopsis* highly ABA-induced1 identifies AT-Hook–Like10 phosphorylation required for stress growth regulation. Proc. Natl. Acad. Sci. USA.

[267] Li H., Han J., Pan J., Liu T., Parker C.E., Borchers C.H. (2017). Current trends in quantitative proteomics - an update. J. Mass Spectrom..

[268] Fan S., Meng Y., Song M., Pang C., Wei H., Liu J., Zhan X., Lan J., Feng C., Zhang S. (2014). Quantitative Phosphoproteomics analysis of nitric oxide–responsive phosphoproteins in cotton leaf. PLoS ONE.

[269] Zhang G.-L., Zhu Y., Fu W.-D., Wang P., Zhang R.-H., Zhang Y.-L., Song Z., Xia G.-X., Wu J.-H. (2016). iTRAQ Protein profile differential analysis of dormant and germinated grassbur twin seeds reveals that ribosomal synthesis and carbohydrate metabolism promote germination possibly through the PI3K pathway. Plant Cell Physiol..

[270] Moothoo-Padayachie A., Macdonald A., Varghese B., Pammenter N.W., Govender P. (2018). Sershen uncovering the basis of viability loss in desiccation sensitive *Trichilia dregeana* seeds using differential quantitative protein expression profiling by iTRAQ. J. Plant Physiol..

[271] Schoberer J., Strasser R. (2018). Plant glyco-biotechnology. Semin. Cell Dev. Biol..

[272] Aalberse R., Koshte V., Clemens J. (1981). Immunoglobulin E antibodies that crossreact with vegetable foods, pollen, and *Hymenoptera venom*. J. Allergy Clin. Immunol..

[273] La Fuente M.D., López-pedrouso M., Alonso J., Santalla M., De Ron A.M., Álvarez G., Zapata C. (2012). In-depth characterization of the phaseolin protein diversity of common bean (*Phaseolus vulgaris* L.) Based on two-dimensional electrophoresis and mass spectrometry. Food Technol. Biotechnol..

[274] Mehta-D’souza P. (2018). Detection of glycoproteins in polyacrylamide gels using Pro-Q Emerald 300 Dye, a fluorescent periodate schiff-base stain. Protein Gel Detection and Imaging. Methods in Molecular Biology.

[275] Weiss W., Postel W., Görg A. (1992). Qualitative and quantitative changes in barley seed protein patterns during the malting process analyzed by sodium dodecyl sulfate-polyacrylamide gel electrophoresis with respect to malting quality. Electrophoresis.

[276] Weiss W., Postel W., Görg A. (1992). Application of sequential extraction procedures and glycoprotein blotting for the characterization of the 2-D polypeptide patterns of barley seed proteins. Electrophoresis.

[277] Madera M., Mann B., Mechref Y., Novotny M.V. (2008). Efficacy of glycoprotein enrichment by microscale lectin affinity chromatography. J. Sep. Sci..

[278] Ostrowski M., Hetmann A., Jakubowska A. (2015). Indole-3-acetic acid UDP-glucosyltransferase from immature seeds of pea is involved in modification of glycoproteins. Phytochemistry.

[279] Wang T., Hu X.-C., Cai Z.-P., Voglmeir J., Liu L. (2017). Qualitative and quantitative analysis of carbohydrate modification on glycoproteins from seeds of *Ginkgo biloba*. J. Agric. Food Chem..

[280] Rao R.S.P., Thelen J.J., Miernyk J.A. (2014). Is Lys-Ne{open}-acetylation the next big thing in post-translational modifications?. Trends Plant Sci..

[281] Smith-Hammond C.L., Swatek K.N., Johnston M.L., Thelen J.J., Miernyk J.A. (2014). Initial description of the developing soybean seed protein Lys-Nε-acetylome. J. Proteomics.

[282] He D., Wang Q., Li M., Damaris R.N., Yi X., Cheng Z., Yang P. (2016). Global Proteome analyses of lysine acetylation and succinylation reveal the widespread involvement of both modification in metabolism in the embryo of germinating rice seed. J. Proteome Res..

[283] Traverso J.A., Meinnel T., Giglione C. (2008). Expanded impact of protein N-myristoylation in plants. Plant Signal. Behav..

[284] Running M.P. (2014). The role of lipid post–translational modification in plant developmental processes. Front. Plant Sci..

[285] Kalemba E.M., Litkowiec M. (2015). Functional characterization of a dehydrin protein from *Fagus sylvatica* seeds using experimental and in silico approaches. Plant Physiol. Biochem..

[286] Friso G., van Wijk K.J. (2015). Update: Post-translational protein modifications in plant metabolism. Plant Physiol..

[287] Mann M., Højrup P., Roepstorff P. (1993). Use of mass spectrometric molecular weight information to identify proteins in sequence databases. Biol. Mass Spectrom..

[288] Henzel W.J., Watanabe C., Stults J.T. (2003). Protein identification: The origins of peptide mass fingerprinting. J. Am. Soc. Mass Spectrom..

[289] Eng J.K., McCormack A.L., Yates J.R. (1994). An approach to correlate tandem mass spectral data of peptides with amino acid sequences in a protein database. J. Am. Soc. Mass Spectrom..

[290] Cottrell J.S. (2011). Protein identification using MS/MS data. J. Proteomics.

[291] Stein S.E., Scott D.R. (1994). Optimization and testing of mass spectral library search algorithms for compound identification. J. Am. Soc. Mass Spectrom..

[292] (2005). Collisionally induced dissociation of protonated peptide ions and the interpretation of product ion spectra. Protein Sequencing and Identification Using Tandem Mass Spectrometry.

[293] Mann M., Wilm M. (1994). Error-tolerant identification of peptides in sequence databases by peptide sequence tags. Anal. Chem..

[294] Tabb D.L. (2015). The SEQUEST family tree. J. Am. Soc. Mass Spectrom..

[295] Perkins D.N., Pappin D.J.C., Creasy D.M., Cottrell J.S. (1999). Probability-based protein identification by searching sequence databases using mass spectrometry data. Electrophoresis.

[296] Cox J., Neuhauser N., Michalski A., Scheltema R.A., Olsen J.V., Mann M. (2011). Andromeda: A peptide search engine integrated into the MaxQuant environment. J. Proteome Res..

[297] Eng J.K., Searle B.C., Clauser K.R., Tabb D.L. (2011). A Face in the Crowd: Recognizing peptides through database search. Mol. Cell. Proteomics.

[298] Moore R.E., Young M.K., Lee T.D. (2002). Qscore: An algorithm for evaluating SEQUEST database search results. J. Am. Soc. Mass Spectrom..

[299] Peng J., Elias J.E., Thoreen C.C., Licklider L.J., Gygi S.P. (2003). Evaluation of multidimensional chromatography coupled with tandem mass spectrometry (LC/LC−MS/MS) for large-scale protein analysis: The yeast proteome. J. Proteome Res..

[300] Reiter L., Claassen M., Schrimpf S.P., Jovanovic M., Schmidt A., Buhmann J.M., Hengartner M.O., Aebersold R. (2009). Protein identification false discovery rates for very large proteomics data sets generated by tandem mass spectrometry. Mol. Cell. Proteomics.

[301] Savitski M.M., Wilhelm M., Hahne H., Kuster B., Bantscheff M. (2015). A scalable approach for protein false discovery rate estimation in large proteomic data sets. Mol. Cell. Proteomics.

[302] Elias J.E., Gygi S.P. (2007). Target-decoy search strategy for increased confidence in large-scale protein identifications by mass spectrometry. Nat. Methods.

[303] Sinitcyn P., Rudolph J.D., Cox J. (2018). Computational Methods for understanding mass spectrometry–based shotgun proteomics data. Annu. Rev. Biomed. Data Sci..

[304] Angel T.E., Aryal U.K., Hengel S.M., Baker E.S., Kelly R.T., Robinson E.W., Smith R.D. (2012). Mass spectrometry-based proteomics: Existing capabilities and future directions. Chem. Soc. Rev..

[305] Hansen B.T., Davey S.W., Ham A.-J.L., Liebler D.C. (2005). P-Mod: An algorithm and software to map modifications to peptide sequences using tandem MS data. J. Proteome Res..

[306] Kertesz V., Connelly H.M., Erickson B.K., Hettich R.L. (2009). PTMSearchPlus: Software tool for automated protein identification and post-translational modification characterization by integrating accurate intact protein mass and bottom-up mass spectrometric data searches. Anal. Chem..

[307] Na S., Paek E. (2015). Software eyes for protein post-translational modifications. Mass Spectrom. Rev..

[308] Medzihradszky K.F., Chalkley R.J. (2015). Lessons in *de novo* peptide sequencing by tandem mass spectrometry. Mass Spectrom. Rev..

[309] Jeong K., Kim S., Pevzner P.A. (2013). UniNovo: A universal tool for de novo peptide sequencing. Bioinformatics.

[310] Hu J. (2005). The importance of experimental design in proteomic mass spectrometry experiments: Some cautionary tales. Briefings Funct. Genomics Proteomics.

[311] Schulze W.X., Usadel B. (2010). Quantitation in mass-spectrometry-based proteomics. Annu. Rev. Plant Biol..

[312] Oda Y., Huang K., Cross F.R., Cowburn D., Chait B.T. (1999). Accurate quantitation of protein expression and site-specific phosphorylation. Proc. Natl. Acad. Sci. USA.

[313] Nahnsen S., Bielow C., Reinert K., Kohlbacher O. (2013). Tools for label-free peptide quantification. Mol. Cell. Proteomics.

[314] Mueller L.N., Rinner O., Schmidt A., Letarte S., Bodenmiller B., Brusniak M.-Y., Vitek O., Aebersold R., Müller M. (2007). SuperHirn – a novel tool for high resolution LC-MS-based peptide/protein profiling. Proteomics.

[315] Cox J., Mann M. (2008). MaxQuant enables high peptide identification rates, individualized p.p.b.-range mass accuracies and proteome-wide protein quantification. Nat. Biotechnol..

[316] Progenesis QI User Guide: Analysis Workflow Guidelines for DDA Data. http://www.nonlinear.com/progenesis/qi-for-proteomics/v4.1/user-guide/.

[317] Bertsch A., Gröpl C., Reinert K., Kohlbacher O., Hamacher M., Eisenacher M., Stephan C. (2011). OpenMS and TOPP: Open source software for LC-MS data analysis. Data Mining in Proteomics. Methods in Molecular Biology (Methods and Protocols).

[318] https://www.thermofisher.com.

[319] http://tools.proteomecenter.org/software.php.

[320] Ishihama Y., Oda Y., Tabata T., Sato T., Nagasu T., Rappsilber J., Mann M. (2005). Exponentially modified protein abundance index (emPAI) for estimation of absolute protein amount in proteomics by the number of sequenced peptides per protein. Mol. Cell. Proteomics.

[321] Braisted J.C., Kuntumalla S., Vogel C., Marcotte E.M., Rodrigues A.R., Wang R., Huang S.-T., Ferlanti E.S., Saeed A.I., Fleischmann R.D. (2008). The APEX quantitative proteomics tool: Generating protein quantitation estimates from LC-MS/MS proteomics results. BMC Bioinform..

[322] Sun A., Zhang J., Wang C., Yang D., Wei H., Zhu Y., Jiang Y., He F. (2009). Modified spectral count index (mSCI) for Estimation of protein abundance by protein relative identification possibility (RIPpro): A new proteomic technological parameter. J. Proteome Res..

[323] Silva J.C., Gorenstein M.V., Li G.-Z., Vissers J.P.C., Geromanos S.J. (2006). Absolute quantification of proteins by LCMS E. Mol. Cell. Proteomics.

[324] Clough T., Key M., Ott I., Ragg S., Schadow G., Vitek O. (2009). Protein quantification in label-free LC-MS experiments. J. Proteome Res..

[325] Monroe M.E., Tolić N., Jaitly N., Shaw J.L., Adkins J.N., Smith R.D. (2007). VIPER: An advanced software package to support high-throughput LC-MS peptide identification. Bioinformatics.

[326] Pluskal T., Castillo S., Villar-Briones A., Orešič M. (2010). MZmine 2: Modular framework for processing, visualizing, and analyzing mass spectrometry-based molecular profile data. BMC Bioinform..

[327] Bairoch A., Apweiler R. (1999). The SWISS-PROT protein sequence data bank and its supplement TrEMBL in 1999. Nucleic Acids Res..

[328] Simpson J.C., Pepperkok R. (2003). Localizing the proteome. Genome Biol..

[329] Huh W.-K., Falvo J.V., Gerke L.C., Carroll A.S., Howson R.W., Weissman J.S., O’Shea E.K. (2003). Global analysis of protein localization in budding yeast. Nature.

[330] Yu C.-S., Chen Y.-C., Lu C.-H., Hwang J.-K. (2006). Prediction of protein subcellular localization. Proteins Struct. Funct. Bioinforma..

[331] Horton P., Park K.-J., Obayashi T., Fujita N., Harada H., Adams-Collier C.J., Nakai K. (2007). WoLF PSORT: Protein localization predictor. Nucleic Acids Res..

[332] Briesemeister S., Rahnenführer J., Kohlbacher O. (2010). YLoc—An interpretable web server for predicting subcellular localization. Nucleic Acids Res..

[333] Savojardo C., Martelli P.L., Fariselli P., Profiti G., Casadio R. (2018). BUSCA: An integrative web server to predict subcellular localization of proteins. Nucleic Acids Res..

[334] Sperschneider J., Catanzariti A.-M., DeBoer K., Petre B., Gardiner D.M., Singh K.B., Dodds P.N., Taylor J.M. (2017). LOCALIZER: Subcellular localization prediction of both plant and effector proteins in the plant cell. Sci. Rep..

[335] Chou K.-C., Shen H.-B. (2010). Plant-mPLoc: A Top-Down Strategy to Augment the Power for Predicting Plant Protein Subcellular Localization. PLoS ONE.

[336] Käll L., Krogh A., Sonnhammer E.L. (2004). A combined transmembrane topology and signal peptide prediction method. J. Mol. Biol..

[337] Bateman A., Martin M.J., O’Donovan C., Magrane M., Alpi E., Antunes R., Bely B., Bingley M., Bonilla C., Britto R. (2017). UniProt: The universal protein knowledgebase. Nucleic Acids Res..

[338] Kanehisa M., Goto S., Sato Y., Kawashima M., Furumichi M., Tanabe M. (2014). Data, information, knowledge and principle: Back to metabolism in KEGG. Nucleic Acids Res..

[339] Ogata H., Goto S., Sato K., Fujibuchi W., Bono H., Kanehisa M. (1999). KEGG: Kyoto encyclopedia of genes and genomes. Nucleic Acids Res..

[340] Klie S., Nikoloski Z. (2012). The Choice between mapman and gene ontology for automated gene function prediction in plant science. Front. Genet..

[341] Kanehisa M., Sato Y., Morishima K. (2016). BlastKOALA and GhostKOALA: KEGG tools for functional characterization of genome and metagenome sequences. J. Mol. Biol..

[342] Kanehisa M., Sato Y., Kawashima M., Furumichi M., Tanabe M. (2016). KEGG as a reference resource for gene and protein annotation. Nucleic Acids Res..

[343] Mi H., Muruganujan A., Huang X., Ebert D., Mills C., Guo X., Thomas P.D. (2019). Protocol update for large-scale genome and gene function analysis with the PANTHER classification system (v.14.0). Nat. Protoc..

[344] Usadel B., Poree F., Nagel A., Lohse M., Czedik-Eysenberg A., Stitt M. (2009). A guide to using MapMan to visualize and compare omics data in plants: A case study in the crop species, maize. Plant. Cell Environ..

[345] Lohse M., Nagel A., Herter T., May P., Schroda M., Zrenner R., Tohge T., Fernie A.R., Stitt M., Usadel B. (2014). Mercator: A fast and simple web server for genome scale functional annotation of plant sequence data. Plant. Cell Environ..

[346] Saha I., Klingström T., Forsberg S., Wikander J., Zubek J., Kierczak M., Plewczynski D. (2014). Evaluation of machine learning algorithms on protein-protein interactions. Advances in Intelligent Systems and Computing.

[347] Hayashi T., Matsuzaki Y., Yanagisawa K., Ohue M., Akiyama Y. (2018). MEGADOCK-Web: An integrated database of high-throughput structure-based protein-protein interaction predictions. BMC Bioinform..

[348] Liu S., Liu C., Deng L. (2018). Machine Learning approaches for protein–protein interaction hot spot prediction: Progress and comparative assessment. Molecules.

[349] Schrader M. (2018). Origins, technological development, and applications of peptidomics. Methods in Molecular Biology.

[350] Ohyama K., Ogawa M., Matsubayashi Y. (2008). Identification of a biologically active, small, secreted peptide in *Arabidopsis* by in silico gene screening, followed by LC-MS-based structure analysis. Plant J..

[351] Farrokhi N., Whitelegge J.P., Brusslan J.A. (2008). Plant peptides and peptidomics. Plant Biotechnol. J..

[352] Segonzac C., Monaghan J. (2019). Modulation of plant innate immune signaling by small peptides. Curr. Opin. Plant Biol..

[353] Pan S., Agyei D., Jeevanandam J., Danquah M.K. (2019). Bio-active peptides: Role in plant growth and defense. Natural Bio-Active Compounds.

[354] Gancheva M.S., Malovichko Y.V., Poliushkevich L.O., Dodueva I.E., Lutova L.A. (2019). Plant peptide hormones. Russ. J. Plant Physiol..

[355] Stührwohldt N., Schaller A. (2019). Regulation of plant peptide hormones and growth factors by post-translational modification. Plant Biol..

[356] Oh E., Seo P.J., Kim J. (2018). Signaling peptides and receptors coordinating plant root development. Trends Plant Sci..

[357] Pan H., Wang D. (2017). Nodule cysteine-rich peptides maintain a working balance during nitrogen-fixing symbiosis. Nat. Plants.

[358] Ayala-Niño A., Rodríguez-Serrano G.M., González-Olivares L.G., Contreras-López E., Regal-López P., Cepeda-Saez A. (2019). Sequence Identification of bioactive peptides from amaranth seed proteins (*Amaranthus hypochondriacus* spp.). Molecules.

[359] Sharma S., Verma H.N., Sharma N.K. (2014). Cationic bioactive peptide from the seeds of *Benincasa hispida*. Int. J. Pept..

[360] Maruyama N., Mikami B., Utsumi S. (2011). The development of transgenic crops to improve human health by advanced utilization of seed storage proteins. Biosci. Biotechnol. Biochem..

[361] Prak K., Maruyama Y., Maruyama N., Utsumi S. (2006). Design of genetically modified soybean proglycinin A1aB1b with multiple copies of bioactive peptide sequences. Peptides.

[362] Pu C., Tang W. (2017). The antibacterial and antibiofilm efficacies of a liposomal peptide originating from rice bran protein against: Listeria monocytogenes. Food Funct..

[363] Chai T.-T., Tan Y.-N., Ee K.-Y., Xiao J., Wong F.-C. (2019). Seeds, fermented foods, and agricultural by-products as sources of plant-derived antibacterial peptides. Crit. Rev. Food Sci. Nutr..

[364] Yang J., Hu L., Cai T., Chen Q., Ma Q., Yang J., Meng C., Hong J. (2018). Purification and identification of two novel antioxidant peptides from perilla (*Perilla frutescens* L. Britton) seed protein hydrolysates. PLoS ONE.

[365] Ye N., Hu P., Xu S., Chen M., Wang S., Hong J., Chen T., Cai T. (2018). Preparation and characterization of antioxidant peptides from carrot seed protein. J. Food Qual..

[366] Aguilar-Toalá J.E., Liceaga A.M. (2020). Identification of chia seed (*Salvia hispanica* L.) peptides with enzyme inhibition activity towards skin-aging enzymes. Amino Acids.

[367] Dallas D.C., Guerrero A., Parker E.A., Robinson R.C., Gan J., German J.B., Barile D., Lebrilla C.B. (2015). Current peptidomics: Applications, purification, identification, quantification, and functional analysis. Proteomics.

[368] Smith C.A., Want E.J., O’Maille G., Abagyan R., Siuzdak G. (2006). XCMS: Processing mass spectrometry data for metabolite profiling using nonlinear peak alignment, matching, and identification. Anal. Chem..

[369] Meyrand M., Dallas D.C., Caillat H., Bouvier F., Martin P., Barile D. (2013). Comparison of milk oligosaccharides between goats with and without the genetic ability to synthesize αs1-casein. Small Rumin. Res..

[370] Guerrero A., Dallas D.C., Contreras S., Chee S., Parker E.A., Sun X., Dimapasoc L., Barile D., German J.B., Lebrilla C.B. (2014). Mechanistic Peptidomics: Factors that dictate specificity in the formation of endogenous peptides in human milk. Mol. Cell. Proteomics.

[371] Kaur H., Garg A., Raghava G.P.S. (2007). Raghava PEPstr: A *de novo* method for tertiary structure prediction of small bioactive peptides. Protein Pept. Lett..

[372] Thévenet P., Shen Y., Maupetit J., Guyon F., Derreumaux P., Tufféry P. (2012). PEP-FOLD: An updated de novo structure prediction server for both linear and disulfide bonded cyclic peptides. Nucleic Acids Res..

[373] Beaufays J., Lins L., Thomas A., Brasseur R. (2012). *In silico* predictions of 3D structures of linear and cyclic peptides with natural and non-proteinogenic residues. J. Pept. Sci..

[374] Wienkoop S., Staudinger C., Hoehenwarter W., Weckwerth W., Egelhofer V. (2012). ProMEX—A mass spectral reference database for plant proteomics. Front. Plant Sci..

[375] Colgrave M.L., Poth A.G., Kaas Q., Craik D.J. (2010). A new “era” for cyclotide sequencing. Biopolymers.

[376] Fesenko I.A., Arapidi G.P., Skripnikov A.Y., Alexeev D.G., Kostryukova E.S., Manolov A.I., Altukhov I.A., Khazigaleeva R.A., Seredina A.V., Kovalchuk S.I. (2015). Specific pools of endogenous peptides are present in gametophore, protonema, and protoplast cells of the moss Physcomitrella patens. BMC Plant Biol..

[377] Del Río L.A. (2015). ROS and RNS in plant physiology: An overview. J. Exp. Bot..

[378] Turkan I. (2018). ROS and RNS: Key signalling molecules in plants. J. Exp. Bot..

[379] Birben E., Sahiner U.M., Sackesen C., Erzurum S., Kalayci O. (2012). Oxidative stress and antioxidant defense. World Allergy Organ. J..

[380] Mock H.P., Dietz K.J. (2016). Redox proteomics for the assessment of redox-related posttranslational regulation in plants. Biochim. Biophys. Acta Proteins Proteomics.

[381] Dietz K.J. (2011). Peroxiredoxins in plants and cyanobacteria. Antioxid. Redox Signal..

[382] Huang J., Willems P., Wei B., Tian C., Ferreira R.B., Bodra N., Martínez Gache S.A., Wahni K., Liu K., Vertommen D. (2019). Mining for protein S-sulfenylation in *Arabidopsis* uncovers redox-sensitive sites. Proc. Natl. Acad. Sci. USA.

[383] Wang H., Wang S., Lu Y., Alvarez S., Hicks L.M., Ge X., Xia Y. (2012). Proteomic analysis of early-responsive redox-sensitive proteins in *Arabidopsis*. J. Proteome Res..

[384] Akter S., Carpentier S., Van Breusegem F., Messens J. (2017). Identification of dimedone-trapped sulfenylated proteins in plants under stress. Biochem. Biophys. Reports.

[385] De Smet B., Willems P., Fernandez-Fernandez A.D., Alseekh S., Fernie A.R., Messens J., Van Breusegem F. (2019). *In vivo* detection of protein cysteine sulfenylation in plastids. Plant J..

[386] Akter S., Fu L., Jung Y., Lo Conte M., Lawson J.R., Lowther W.T., Sun R., Liu K., Yang J., Carroll K.S. (2018). Chemical proteomics reveals new targets of cysteine sulfinic acid reductase. Nat. Chem. Biol..

[387] Nietzel T., Mostertz J., Ruberti C., Née G., Fuchs P., Wagner S., Moseler A., Müller-Schüssele S.J., Benamar A., Poschet G. (2020). Redox-mediated kick-start of mitochondrial energy metabolism drives resource-efficient seed germination. Proc. Natl. Acad. Sci. USA.

[388] Ratajczak E., Staszak A., Wojciechowska N., Bagniewska-Zadworna A., Dietz K. (2019). Regulation of thiol metabolism as a factor that influences the development and storage capacity of beech seeds. J. Plant Physiol..

[389] Ratajczak E., Małecka A., Ciereszko I., Staszak A. (2019). Mitochondria are important determinants of the aging of seeds. Int. J. Mol. Sci..

[390] Smolikova G., Kreslavski V., Shiroglazova O., Bilova T., Sharova E., Frolov A., Medvedev S. (2018). Photochemical activity changes accompanying the embryogenesis of pea (*Pisum sativum*) with yellow and green cotyledons. Funct. Plant Biol..

[391] Landry E.J., Fuchs S.J., Hu J. (2016). Carbohydrate composition of mature and immature faba bean seeds. J. Food Compos. Anal..

[392] Bailly C., Kraner I., Kermode A.R. (2011). Analyses of ROS and antioxidants in relation to seed longevity and germination. Seed Dormancy:Methods and Protocols.

[393] Oracz K., Bouteau H.E.-M., Farrant J.M., Cooper K., Belghazi M., Job C., Job D., Corbineau F., Bailly C. (2007). ROS production and protein oxidation as a novel mechanism for seed dormancy alleviation. Plant J..

[394] Leymarie J., Vitkauskaité G., Hoang H.H., Gendreau E., Chazoule V., Meimoun P., Corbineau F., El-Maarouf-Bouteau H., Bailly C. (2012). Role of reactive oxygen species in the regulation of *Arabidopsis* seed dormancy. Plant Cell Physiol..

[395] Bailly C. (2004). Active oxygen species and antioxidants in seed biology. Seed Sci. Res..

[396] Wolff I.A. (1966). Seed lipids. Science.

[397] Ponquett R.T., Ross G. (1992). Lipid autoxidation and seed ageing: Putative relationships between seed longevity and lipid stability. Seed Sci. Res..

[398] Ouzouline M., Tahani N., Demandre C., El Amrani E., Benhassaine-Kesri G., Serghini Caid H. (2009). Effects of accelerated aging upon the lipid composition of seeds from two soft wheat varieties from Morocco. Grasas y Aceites.

[399] Murthy U.M.N., Kumar P.P., Sun W.Q., Murthy N.U.M., Kumar P.P., Sun W.Q. (2003). Mechanisms of seed ageing under different storage conditions for *Vigna radiata* (L.) Wilczek: Lipid peroxidation, sugar hydrolysis, Maillard reactions and their relationship to glass state transition. J. Exp. Bot..

[400] Hoekstra F.A., Golovina E.A., Tetteroo F.A., Wolkers W.F. (2001). Induction of desiccation tolerance in plant somatic embryos: How exclusive is the protective role of sugars?. Cryobiology.

[401] Wolff S.P., Dean R.T. (1987). Glucose autoxidation and protein modification. The potential role of ‘autoxidative glycosylation’ in diabetes. Biochem. J..

[402] Ayala A., Muñoz M.F., Argüelles S. (2014). Lipid Peroxidation: Production, metabolism, and signaling mechanisms of malondialdehyde and 4-hydroxy-2-nonenal. Oxid. Med. Cell. Longev..

[403] Vistoli G., De Maddis D., Cipak A., Zarkovic N., Carini M., Aldini G. (2013). Advanced glycoxidation and lipoxidation end products (AGEs and ALEs): An overview of their mechanisms of formation. Free Radic. Res..

[404] Møller I.M., Rogowska-Wrzesinska A., Rao R.S.P. (2011). Protein carbonylation and metal-catalyzed protein oxidation in a cellular perspective. J. Proteomics.

[405] Strelec I., Hlevnjak M. (2008). Accumulation of Amadori and Maillard products in wheat seeds aged under different storage conditions. Croat. Chem. Acta.

[406] Wettlaufer S.H., Leopold A.C. (1991). Relevance of Amadori and Maillard products to seed deterioration. Plant Physiol..

[407] Colville L., Bradley E.L., Lloyd A.S., Pritchard H.W., Castle L., Kranner I. (2012). Volatile fingerprints of seeds of four species indicate the involvement of alcoholic fermentation, lipid peroxidation, and Maillard reactions in seed deterioration during ageing and desiccation stress. J. Exp. Bot..

[408] Greifenhagen U., Nguyen V.D.V.D., Moschner J., Giannis A., Frolov A., Hoffmann R. (2015). Sensitive and site-specific identification of carboxymethylated and carboxyethylated peptides in tryptic digests of proteins and human plasma. J. Proteome Res..

[409] Schmidt R., Böhme D., Singer D., Frolov A. (2015). Specific tandem mass spectrometric detection of AGE-modified arginine residues in peptides. J. Mass Spectrom..

[410] Frolov A., Hoffmann P., Hoffmann R. (2006). Fragmentation behavior of glycated peptides derived fromD-glucose,D-fructose andD-ribose in tandem mass spectrometry. J. Mass Spectrom..

[411] Zhang Q., Frolov A., Tang N., Hoffmann R., van de Goor T., Metz T.O., Smith R.D.R.D. (2007). Application of electron transfer dissociation mass spectrometry in analyses of non-enzymatically glycated peptides. Rapid Commun. Mass Spectrom..

[412] Fedorova M., Frolov A., Hoffmann R. (2010). Fragmentation behavior of Amadori-peptides obtained by non-enzymatic glycosylation of lysine residues with ADP-ribose in tandem mass spectrometry. J. Mass Spectrom..

[413] Ehrlich H., Hanke T., Frolov A., Langrock T., Hoffmann R., Fischer C., Schwarzenbolz U., Henle T., Born R., Worch H. (2009). Modification of collagen in vitro with respect to formation of Nɛ-carboxymethyllysine. Int. J. Biol. Macromol..

[414] Frolov A., Singer D., Hoffmann R. (2006). Sites-specific synthesis of Amadori-modified peptides on solid phase. J. Pept. Sci..

[415] Frolov A., Hoffmann R. (2008). Separation of Amadori peptides from their unmodified analogs by ion-pairing RP-HPLC with heptafluorobutyric acid as ion-pair reagent. Anal. Bioanal. Chem..

[416] Frolov A., Singer D., Hoffmann R. (2007). Solid-phase synthesis of glucose-derived Amadori peptides. J. Pept. Sci..

[417] Greifenhagen U., Frolov A., Hoffmann R. (2015). Oxidative degradation of N ε-fructosylamine-substituted peptides in heated aqueous systems. Amino Acids.

[418] Spiller S., Frolov A., Hoffmann R. (2018). Quantification of specific glycation sites in human serum albumin as prospective type 2 diabetes mellitus biomarkers. Protein Pept. Lett..

[419] Soboleva A., Modzel M., Didio A., Płóciennik H., Kijewska M., Grischina T., Karonova T., Bilova T., Stefanov V., Stefanowicz P. (2017). Quantification of prospective type 2 diabetes mellitus biomarkers by stable isotope dilution with bi-labeled standard glycated peptides. Anal. Methods.

[420] Thornalley P.J., Battah S., Ahmed N., Karachalias N., Agalou S., Babaei-Jadidi R., Dawnay A. (2003). Quantitative screening of advanced glycation endproducts in cellular and extracellular proteins by tandem mass spectrometry. Biochem. J..

[421] Smuda M., Henning C., Raghavan C.T., Johar K., Vasavada A.R., Nagaraj R.H., Glomb M.A. (2015). Comprehensive analysis of maillard protein modifications in human lenses: Effect of age and cataract. Biochemistry.

[422] Rabbani N., Al-Motawa M., Thornalley P.J. (2020). Protein glycation in plants—An under-researched field with much still to discover. Int. J. Mol. Sci..

[423] Leonova T., Popova V., Tsarev A., Henning C., Antonova K., Rogovskaya N., Vikhnina M., Baldensperger T., Soboleva A., Dinastia E. (2020). Does protein glycation impact on the drought-related changes in metabolism and nutritional properties of mature pea (*Pisum sativum* L.) seeds?. Int. J. Mol. Sci..

[424] Dammann P., Sell D.R., Begall S., Strauch C., Monnier V.M. (2012). Advanced Glycation End-Products as Markers of Aging and longevity in the long-lived Ansell’s mole-rat (*Fukomys anselli*). J. Gerontol. Ser. A.

[425] Bilova T., Paudel G., Shilyaev N., Schmidt R., Brauch D., Tarakhovskaya E., Milrud S., Smolikova G., Tissier A., Vogt T. (2017). Global proteomic analysis of advanced glycation end products in the *Arabidopsis* proteome provides evidence for age-related glycation hot spots. J. Biol. Chem..

[426] Vilhena M.B., Franco M.R., Schmidt D., Carvalho G., Azevedo R.A. (2015). Evaluation of protein extraction methods for enhanced proteomic analysis of tomato leaves and roots. An. Acad. Bras. Cienc..

[427] Galland M., He D., Lounifi I., Arc E., Clément G., Balzergue S., Huguet S., Cueff G., Godin B., Collet B. (2017). An integrated “multi-omics” comparison of embryo and endosperm tissue-specific features and their impact on rice seed quality. Front. Plant Sci..

[428] Yin X., He D., Gupta R., Yang P. (2015). Physiological and proteomic analyses on artificially aged *Brassica napus* seed. Front. Plant Sci..

[429] Kołodziejczyk I., Dzitko K., Szewczyk R., Posmyk M.M. (2016). Exogenous melatonin improves corn (*Zea mays* L.) embryo proteome in seeds subjected to chilling stress. J. Plant Physiol..

[430] DeBlasio S.L., Johnson R.S., MacCoss M.J., Gray S.M., Cilia M. (2016). Model system-guided protein interaction mapping for virus isolated from phloem tissue. J. Proteome Res..

[431] Tyanova S., Temu T., Carlson A., Sinitcyn P., Mann M., Cox J. (2015). Visualization of LC-MS/MS proteomics data in MaxQuant. Proteomics.

[432] PEAKS AB Software, Version 2.0. https://www.bioinfor.com/peaks-ab-software/.

[433] Cox B., Kislinger T., Wigle D.A., Kannan A., Brown K., Okubo T., Hogan B., Jurisica I., Frey B., Rossant J. (2007). Integrated proteomic and transcriptomic profiling of mouse lung development and Nmyc target genes. Mol. Syst. Biol..

[434] Röst H.L., Sachsenberg T., Aiche S., Bielow C., Weisser H., Aicheler F., Andreotti S., Ehrlich H.-C., Gutenbrunner P., Kenar E. (2016). OpenMS: A flexible open-source software platform for mass spectrometry data analysis. Nat. Methods.

[435] Proteome Discoverer. User Guide. Software Version 2.2; Thermo Fisher Scientific Inc. 2017. https://assets.thermofisher.com/TFS-Assets/CMD/manuals/Man-XCALI-97808-Proteome-Discoverer-User-ManXCALI97808-EN.pdf.

[436] Park S.K., Yates J.R. (2010). Census for proteome quantification. Current Protocols in Bioinformatics.

[437] Chang C., Zhang J., Han M., Ma J., Zhang W., Wu S., Liu K., Xie H., He F., Zhu Y. (2014). SILVER: An efficient tool for stable isotope labeling LC-MS data quantitative analysis with quality control methods. Bioinformatics.

[438] Bellew M., Coram M., Fitzgibbon M., Igra M., Randolph T., Wang P., May D., Eng J., Fang R., Lin C. (2006). A suite of algorithms for the comprehensive analysis of complex protein mixtures using high-resolution LC-MS. Bioinformatics.

[439] Valot B., Langella O., Nano E., Zivy M. (2011). MassChroQ: A versatile tool for mass spectrometry quantification. Proteomics.

[440] MacLean B., Tomazela D.M., Shulman N., Chambers M., Finney G.L., Frewen B., Kern R., Tabb D.L., Liebler D.C., MacCoss M.J. (2010). Skyline: An open source document editor for creating and analyzing targeted proteomics experiments. Bioinformatics.

[441] Tsou C.-C., Avtonomov D., Larsen B., Tucholska M., Choi H., Gingras A.-C., Nesvizhskii A.I. (2015). DIA-Umpire: Comprehensive computational framework for data-independent acquisition proteomics. Nat. Methods.

[442] Deutsch E.W., Mendoza L., Shteynberg D., Farrah T., Lam H., Tasman N., Sun Z., Nilsson E., Pratt B., Prazen B. (2010). A guided tour of the Trans-Proteomic Pipeline. Proteomics.

[443] Argentini A., Goeminne L.J.E., Verheggen K., Hulstaert N., Staes A., Clement L., Martens L. (2016). moFF: A robust and automated approach to extract peptide ion intensities. Nat. Methods.

[444] An introduction to Mascot Distiller. http://www.matrixscience.com/distiller.html.

[445] Brusniak M.-Y., Bodenmiller B., Campbell D., Cooke K., Eddes J., Garbutt A., Lau H., Letarte S., Mueller L.N., Sharma V. (2008). Corra: Computational framework and tools for LC-MS discovery and targeted mass spectrometry-based proteomics. BMC Bioinform..

[446] Millikin R.J., Solntsev S.K., Shortreed M.R., Smith L.M. (2018). Ultrafast peptide label-free quantification with FlashLFQ. J. Proteome Res..

[447] ProSightPC 4.0 Quick Start Guide. Revision A XCALI-97800; Thermo Fisher Scientific Inc. 2016. https://assets.thermofisher.com/TFS-Assets/CMD/Product-Guides/QS-XCALI-97800-ProSightPC-QSXCALI97800-EN.pdf.

[448] Sanford H., Harnos S. Agilent MassHunter Qualitative Data Analysis; Software B.07.00. 2017. https://www.agilent.com/cs/library/eseminars/public/Session_3_Qualitative_Analysis_Basics.pdf.

[449] Schwacke R., Ponce-Soto G.Y., Krause K., Bolger A.M., Arsova B., Hallab A., Gruden K., Stitt M., Bolger M.E., Usadel B. (2019). MapMan4: A Refined protein classification and annotation framework applicable to multi-omics data analysis. Mol. Plant.

[450] Huerta-Cepas J., Forslund K., Coelho L.P., Szklarczyk D., Jensen L.J., von Mering C., Bork P. (2017). Fast Genome-wide functional annotation through orthology assignment by eggNOG-mapper. Mol. Biol. Evol..

[451] Szklarczyk D., Gable A.L., Lyon D., Junge A., Wyder S., Huerta-Cepas J., Simonovic M., Doncheva N.T., Morris J.H., Bork P. (2019). STRING v11: Protein-protein association networks with increased coverage, supporting functional discovery in genome-wide experimental datasets. Nucleic Acids Res..

